# RNF4 sustains Myc-driven tumorigenesis by facilitating DNA replication

**DOI:** 10.1172/JCI167419

**Published:** 2024-03-26

**Authors:** Joonyoung Her, Haiyan Zheng, Samuel F. Bunting

**Affiliations:** 1 Department of Molecular Biology and Biochemistry and; 2 Biological Mass Spectrometry Facility, Rutgers, The State University of New Jersey, Piscataway, New Jersey, USA.

**Keywords:** Genetics, Oncology, Cell stress, Lymphomas, Ubiquitin-proteosome system

## Abstract

The mammalian SUMO-targeted E3 ubiquitin ligase *Rnf4* has been reported to act as a regulator of DNA repair, but the importance of RNF4 as a tumor suppressor has not been tested. Using a conditional-knockout mouse model, we deleted *Rnf4* in the B cell lineage to test the importance of RNF4 for growth of somatic cells. Although *Rnf4*–conditional-knockout B cells exhibited substantial genomic instability, *Rnf4* deletion caused no increase in tumor susceptibility. In contrast, *Rnf4* deletion extended the healthy lifespan of mice expressing an oncogenic c-*myc* transgene. *Rnf4* activity is essential for normal DNA replication, and in its absence, there was a failure in ATR-CHK1 signaling of replication stress. Factors that normally mediate replication fork stability, including members of the Fanconi anemia gene family and the helicases PIF1 and RECQL5, showed reduced accumulation at replication forks in the absence of RNF4. RNF4 deficiency also resulted in an accumulation of hyper-SUMOylated proteins in chromatin, including members of the SMC5/6 complex, which contributes to replication failure by a mechanism dependent on RAD51. These findings indicate that RNF4, which shows increased expression in multiple human tumor types, is a potential target for anticancer therapy, especially in tumors expressing c-*myc*.

## Abstract 2

The RNF4 protein, which regulates the stability of other proteins, is required for normal cellular proliferation and contributes to the growth of cancer cells.

## Graphical abstract

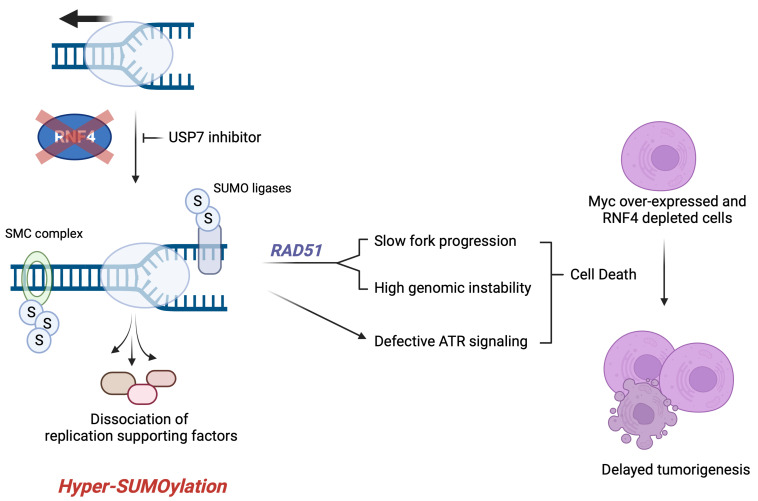

## Introduction

Healthy cell growth requires posttranslational modification of proteins by addition of ubiquitin or the small ubiquitin-like modifier (SUMO) (1, 2). Ubiquitination and SUMOylation regulate almost every aspect of cellular physiology by modifying the function and stability of cellular proteins. DNA replication and repair are examples of the importance of SUMOylation and ubiquitination, which act in coordination to ensure productive outcomes in each case (3, 4).

In mammalian cells, RING finger protein 4 (RNF4) is a key SUMO-targeted E3 ubiquitin ligase (STUbL) that has a central importance in linking protein regulation by SUMOylation and ubiquitination. RNF4 contains four SUMO-interacting motifs (SIMs), which target the protein to SUMOylated substrates. The E3 ubiquitin ligase activity of homodimeric RNF4 subsequently transfers ubiquitin from one of a number of different E2 conjugating enzymes to the substrate (5). RNF4 is known to target the PML-RARA fusion protein that is frequently found in acute promyelocytic leukemia (6). In response to arsenic trioxide treatment, PML undergoes increased SUMOylation, making it an improved target for RNF4 STUbL activity, leading to degradation of the PML-RARA fusion protein and successful treatment outcomes even without use of conventional cytotoxic chemotherapy (7).

In addition to regulation of PML stability, RNF4 is implicated in a range of cellular processes. Several studies, using siRNA knockdown, mutant cell lines, or DT40 cells, reported defects in the cellular response to DNA damage after loss of RNF4 activity (8–12). In particular, efficiency of the homologous recombination (HR) pathway was reported to be reduced in si*RNF4* cells, as a consequence of a failure to load RPA and RAD51 at resected DNA double-strand breaks (13, 14). RNF4 also promotes normal chromosome segregation, based on its ability to remove toxic DNA-protein cross-links that interfere with mitosis (15). Mutations affecting other components of the HR machinery, such as *BRCA1* or *BRCA2*, increase the rate of genetic mutations, leading to substantially increased tumor risk (16). Around 6% of primary cancer cases are estimated to be affected by HR deficiency (17), and identifying these cases is of substantial importance for treatment, because HR-deficient cancers show greatly increased sensitivity to poly(ADP-ribose) polymerase (PARP) inhibitors and platinum-based cross-linking agents (18). Human *RNF4* mutations that might predispose affected individuals to cancer have not yet been reported, however, and the importance of *RNF4* as a tumor suppressor is unknown.

A challenge to evaluating the importance of RNF4 as a tumor suppressor is the lack of suitable animal models to test whether loss of RNF4 increases the risk of malignant cell growth. Homozygous deletion of the mouse *Rnf4* gene causes embryonic lethality prior to E15 (19). We now report the use of a conditional-knockout *Rnf4* mouse model to test the importance of *Rnf4* for DNA repair, and to evaluate how *Rnf4* deficiency contributes to tumor susceptibility. We find that deletion of *Rnf4* causes genomic instability and reduced cellular viability. These phenotypes of *Rnf4* deficiency are an outcome of defective DNA replication, and can be partially rescued by inhibition of the ubiquitin isopeptidase USP7. The increased genomic instability of *Rnf4*-deficient cells does not lead to a higher frequency of malignant neoplasms, however, even on a p53-deficient background. *Rnf4* deficiency instead significantly delayed the rate of tumor formation in a mouse model of c-*myc* overexpression, indicating that *Rnf4* contributes to the growth of incipient tumors. These results demonstrate that RNF4 is an essential factor for DNA replication and a potential novel target for anticancer therapy.

## Results

### Rnf4 deletion causes genomic instability and cell death in primary B cells.

**Figure 1:**
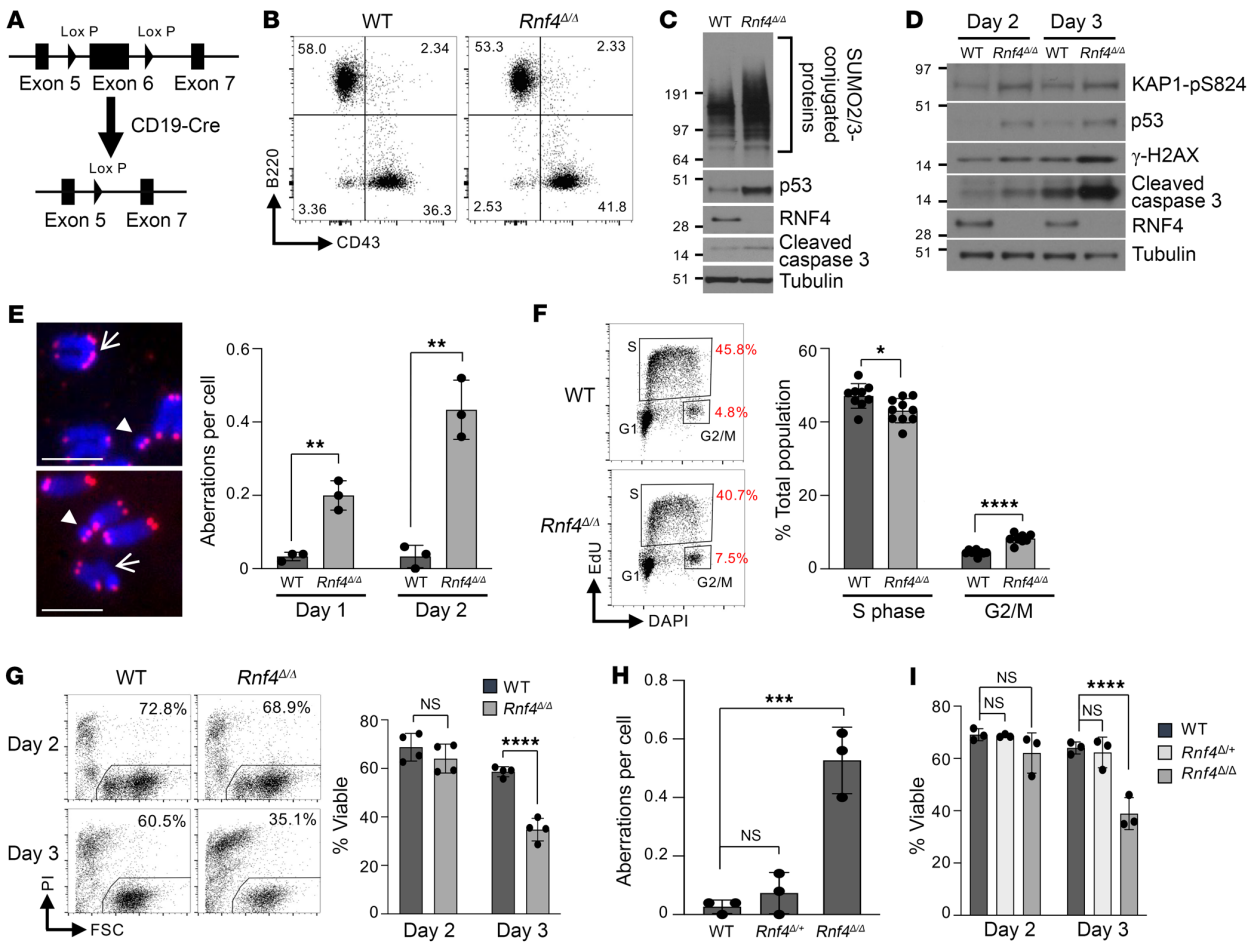
Genomic instability and apoptosis in RNF4-conditional-knockout B cells. (**A**) Schematic representation of Cre-induced deletion of *Rnf4* exon 6. (**B**) Flow cytometry analysis of cell populations in the spleens of WT and *Rnf4*-conditional-knockout (*Rnf4^Δ/Δ^*) mice. (**C**) Western blot analysis of protein expression in *Rnf4^Δ/Δ^* B cells after 2 days of activation in vitro. (**D**) Western blot to measure induction of markers of DNA damage signaling in activated WT and *Rnf4^Δ/Δ^* B cells after 2 days and 3 days of in vitro culture. (**E**) Analysis of chromosome aberrations in metaphase spreads prepared from WT and *Rnf4^Δ/Δ^* B cells after 1 or 2 days of in vitro culture. Arrows show examples of chromosome rearrangements, and arrowheads show examples of chromosome breaks. (**F**) Cell cycle analysis of fixed B cells after 48 hours of activation in vitro. (**G**) Flow cytometry analysis of cell death in splenic B cells cultured for 2 or 3 days in vitro. Left: Figures indicate the percentage of cells that remained viable, based on propidium iodide exclusion. (**H**) Chromosome aberrations in metaphase spreads from WT, *Rnf4^Δ/+^*, and *Rnf4^Δ/Δ^* B cells. (**I**) Viability of B cells after 2 and 3 days of in vitro culture, measured by DAPI exclusion. Error bars indicate SD of the mean. *P* values were calculated with unpaired 2-tailed *t* test (**E**–**G**), 1-way ANOVA with Dunnett’s multiple-comparison test (**H**), and 2-way ANOVA with Dunnett’s multiple-comparison test (**I**). *P < 0.05; **P < 0.01; ***P < 0.001; ****P < 0.0001.

To test the importance of RNF4 for growth of somatic cells, we first attempted to rescue embryonic lethality in *Rnf4^–/–^* mice by crossing to *Trp53^–/–^* or *Trp53bp1^–/–^* mouse lines. Co-deletion of p53 or 53BP1 in these lines rescues embryonic lethality in *Brca1^Δ11/Δ11^* mice (20), but we were not able to obtain any viable *Rnf4^–/–^* pups by this approach, indicating that *Rnf4* has an indispensable role in normal embryonic development (Supplemental Table 1; supplemental material available online with this article; https://doi.org/10.1172/JCI167419DS1). We therefore generated a novel, conditional *Rnf4^fl^* allele by introducing *loxP* sites around *Rnf4* exon 6 (Figure 1A). *Rnf4^fl/fl^* mice were crossed to *CD19-Cre* animals to delete *Rnf4* selectively in the B lymphocyte lineage (21). The spleens of these *Rnf4*–conditional-KO (*Rnf4^Δ/Δ^*) mice showed a near-normal population of mature B220^+^ B cells, but RNF4 was undetectable in protein lysates from these cells (Figure 1, B and C). *Rnf4^Δ/Δ^* B cells showed a substantially increased proportion of high–molecular weight SUMOylated species, consistent with the known role of RNF4 in ensuring turnover of SUMOylated proteins (6).

B cells lacking RNF4 showed clear signs of cell stress after 48 hours of in vitro culture, with stabilization of p53, and increased cleavage of caspase-3 (Figure 1C). Phosphorylation of KAP1 and H2AX was also increased (Figure 1D), indicating activation of ATM and ATR after DNA damage (22). Markers of DNA damage and apoptosis became increasingly apparent after 3 days of in vitro B cell culture, indicating an underlying stress that became more acute after a longer period of B cell activation. Analysis of metaphase chromosomes revealed a significant increase in chromosome aberrations in *Rnf4^Δ/Δ^* B cells (Figure 1E). This increase was apparent after 24 hours of in vitro culture, and reached an even greater level after 48 hours of culture. There was additionally an increase in the frequency of sister chromatid exchanges in *Rnf4^Δ/Δ^* cells, indicating increased use of HR-mediated DNA repair (Supplemental Figure 1A). *Rnf4^Δ/Δ^* B cells also showed a disrupted cell cycle, with a slightly reduced proportion of S phase cells, and an increase in the proportion of cells in G_2_ phase (Figure 1F).

Consistent with the increased induction of apoptotic markers, the majority of *Rnf4^Δ/Δ^* B cells became nonviable after 3 days in culture (Figure 1G). Cell death and increased genomic instability were only observed in homozygous *Rnf4^Δ/Δ^* cells, and not in heterozygotes (Figure 1, H and I). Although p53 is clearly stabilized in *Rnf4^Δ/Δ^* cells (Figure 1C), deletion of *Trp53* only partially rescued cell viability and had no significant impact on chromosome instability (Supplemental Figure 1, B and C). RNF4 therefore plays an essential role for ensuring the viability of proliferating primary B cells, and in its absence, cells undergo apoptosis by a combination of p53-mediated and p53-independent processes.

### Rnf4-knockout cells show defects in DNA replication.

**Figure 2:**
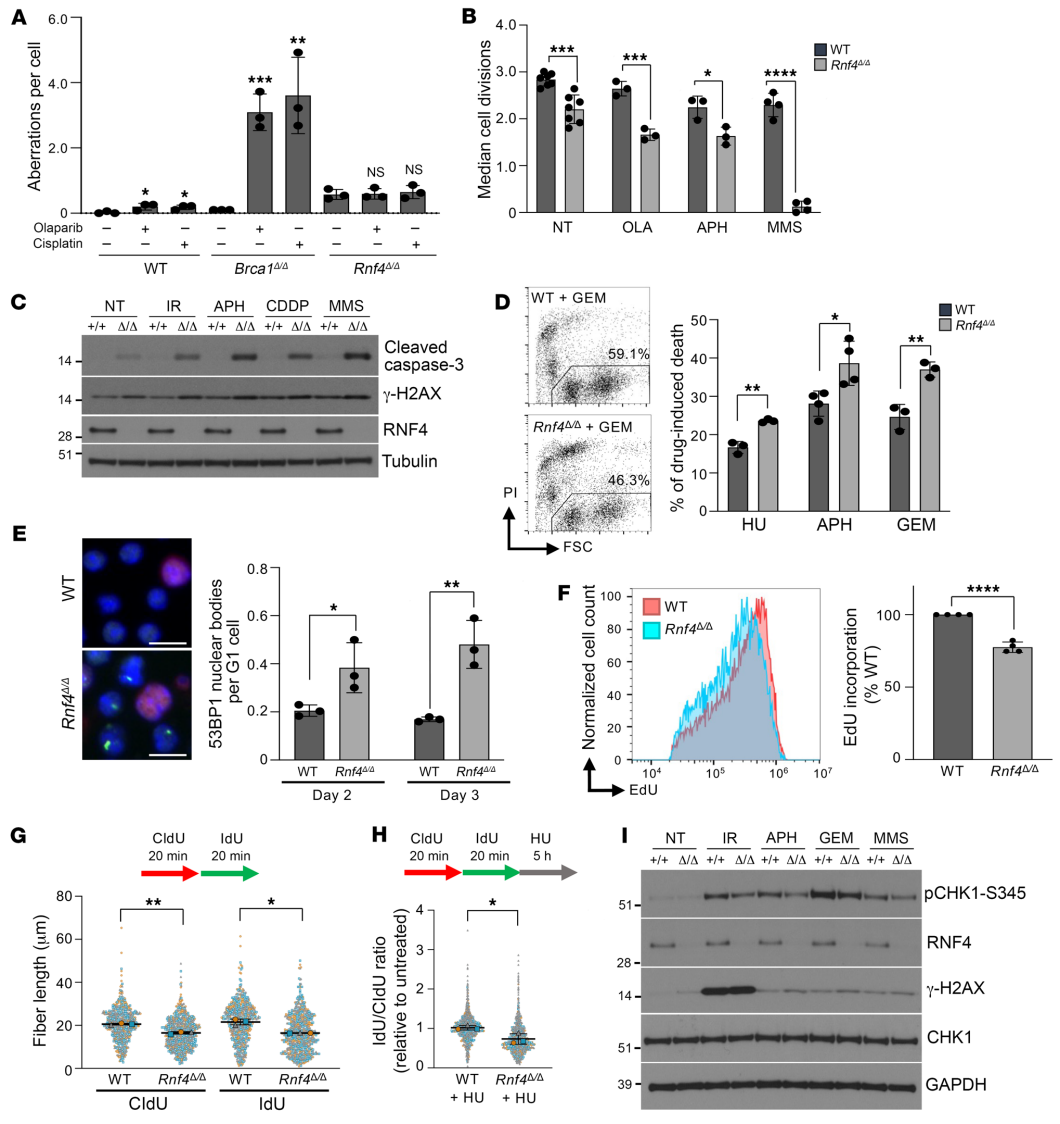
*Rnf4^Δ/Δ^* B cells show defects in DNA replication. (**A**) Analysis of chromosome aberrations in B lymphocytes treated overnight with or without 2 μM olaparib or 1 μM cisplatin. Statistical differences between the means of the treated and nontreated samples are shown. (**B**) Flow cytometry analysis of CFSE dilution to measure B cell growth over 72 hours. Chart shows quantification of cell doublings based on CFSE fluorescence. (**C**) Western blot analysis of caspase-3 cleavage and γ-H2AX after no treatment (NT) or after overnight recovery from treatment with 2 Gy of ionizing radiation (IR), 0.4 μM aphidicolin (APH), 2.5 μM cisplatin (CDDP), or 100 μM MMS. (**D**) Sample flow cytometry data quantifying cell viability after treatment with gemcitabine (GEM). Gated population shows the viable, propidium iodide–negative population. Graph shows proportion of cells that became nonviable 24 hours after treatment with either hydroxyurea (HU) (4 mM, 3 hours), APH (40 μM, 2 hours), or GEM (250 nM, 2 hours). (**E**) Immunofluorescent detection of 53BP1 G_1_ nuclear bodies (green) in B cells after in vitro culture. Cyclin A staining (red) reveals S/G_2_-phase cells. Scale bars: 10 μm. (**F**) EdU uptake measured by flow cytometry. (**G**) Analysis of nascent DNA tract length in WT and *Rnf4^Δ/Δ^* splenic B cells by DNA combing. Mean ± SD of *n* = 3 experiments shown. (**H**) Stability of nascent DNA measured by fiber analysis after 4 mM HU treatment. Mean ± SD of *n* = 3 experiments shown. (**I**) Western blot showing induction of p-CHK1 in WT and *Rnf4^Δ/Δ^* cells after IR (30 Gy, 2 hours recovery), APH (0.4 μM, overnight), GEM (100 nM, 2 hours), or MMS (200 μM, 3 hours). Error bars in **A**, **B**, and **D**–**F** show SD of the mean, with *P* values calculated by unpaired 2-tailed *t* test. *P* values in **G** and **H** were calculated by paired, 2-tailed *t* test. *P < 0.05; **P < 0.01; ***P < 0.001; ****P < 0.0001.

To evaluate the importance of RNF4 for DNA repair, we challenged *Rnf4^Δ/Δ^* cells with a range of compounds that cause DNA damage of various kinds (Supplemental Figure 2A). These agents included olaparib, a PARP inhibitor that produces DNA double-strand breaks in replicating cells; ionizing radiation, which produces DNA double-strand breaks in all phases of the cell cycle; the DNA cross-linking agents mitomycin C and cisplatin (CDDP); and methyl methanesulfonate (MMS), a DNA alkylating agent. In each case, *Rnf4^Δ/Δ^* cells showed an increase in chromosome instability after treatment, but the increase in *Rnf4^Δ/Δ^* cells was not significantly greater than that observed in WT cells after subtraction of the rate of chromosome breaks and rearrangements in the untreated condition (Supplemental Figure 2B). A high frequency of chromosome aberrations was observed after olaparib or cisplatin treatment of *Brca1^Δ11/Δ11^* cells, which express a hypomorphic form of BRCA1 and are HR deficient (23) (Figure 2A). In contrast, *Rnf4^Δ/Δ^* cells showed only a very mild increase in the frequency of chromosome breaks and rearrangements after treatment with olaparib or cisplatin, despite having a higher rate of chromosome instability in the absence of drug treatment. The relatively mild sensitivity of *Rnf4^Δ/Δ^* cells after treatments to induce DNA damage suggests that defects in additional cellular processes may contribute to the genomic instability and poor growth of these cells.

The proliferation and viability of *Rnf4^Δ/Δ^* cells were significantly impacted by treatment with either MMS or the DNA polymerase inhibitor aphidicolin (Figure 2B and Supplemental Figure 2, C and D). These agents also induced strong caspase-3 cleavage in *Rnf4^Δ/Δ^* cells (Figure 2C). MMS produces cytotoxicity through production of single-stranded DNA breaks and by inhibition of DNA replication (24–26). Although we did not detect an increase in single-strand breaks in *Rnf4^Δ/Δ^* cells (Supplemental Figure 2E), we observed that *Rnf4^Δ/Δ^* cells were hypersensitive to the replication inhibitors hydroxyurea, aphidicolin, and gemcitabine (Figure 2D). *Rnf4^Δ/Δ^* cells also showed an increase in 53BP1 G_1_ nuclear bodies, a hallmark of cells that are encountering problems with DNA replication (27, 28) (Figure 2E). *Rnf4^Δ/Δ^* cells consistently incorporated lower levels of the deoxythymidine analog EdU, indicating a lower rate of DNA replication (Figure 2F). To directly measure the activity of individual replication forks, we performed a DNA combing analysis after pulsing cells with CldU and IdU, which revealed that *Rnf4^Δ/Δ^* cells showed a significant reduction in replication fork velocity (Figure 2G). *Rnf4^Δ/Δ^* cells also showed reduced stability of newly replicated DNA after hydroxyurea treatment (Figure 2H). ATR-mediated CHK1 phosphorylation is essential for signaling the presence of replication stress (29), but phosphorylation of CHK1 was defective after induction of replication stress in the absence of RNF4 (Figure 2I). Although replication forks in *Rnf4^Δ/Δ^* cells appeared to restart normally after hydroxyurea treatment (Supplemental Figure 2F), and showed only marginal levels of fork asymmetry (Supplemental Figure 2G), the reduction in replication fork progression induced by MMS treatment in WT cells was not observed in RNF4-conditional-knockout cells (Supplemental Figure 2H). A failure to adequately signal replication stress therefore correlates with reduced replication efficiency in *Rnf4^Δ/Δ^* cells.

### Replication defects and cell death in Rnf4^Δ/Δ^ cells are a consequence of deregulated SUMOylation.

**Figure 3:**
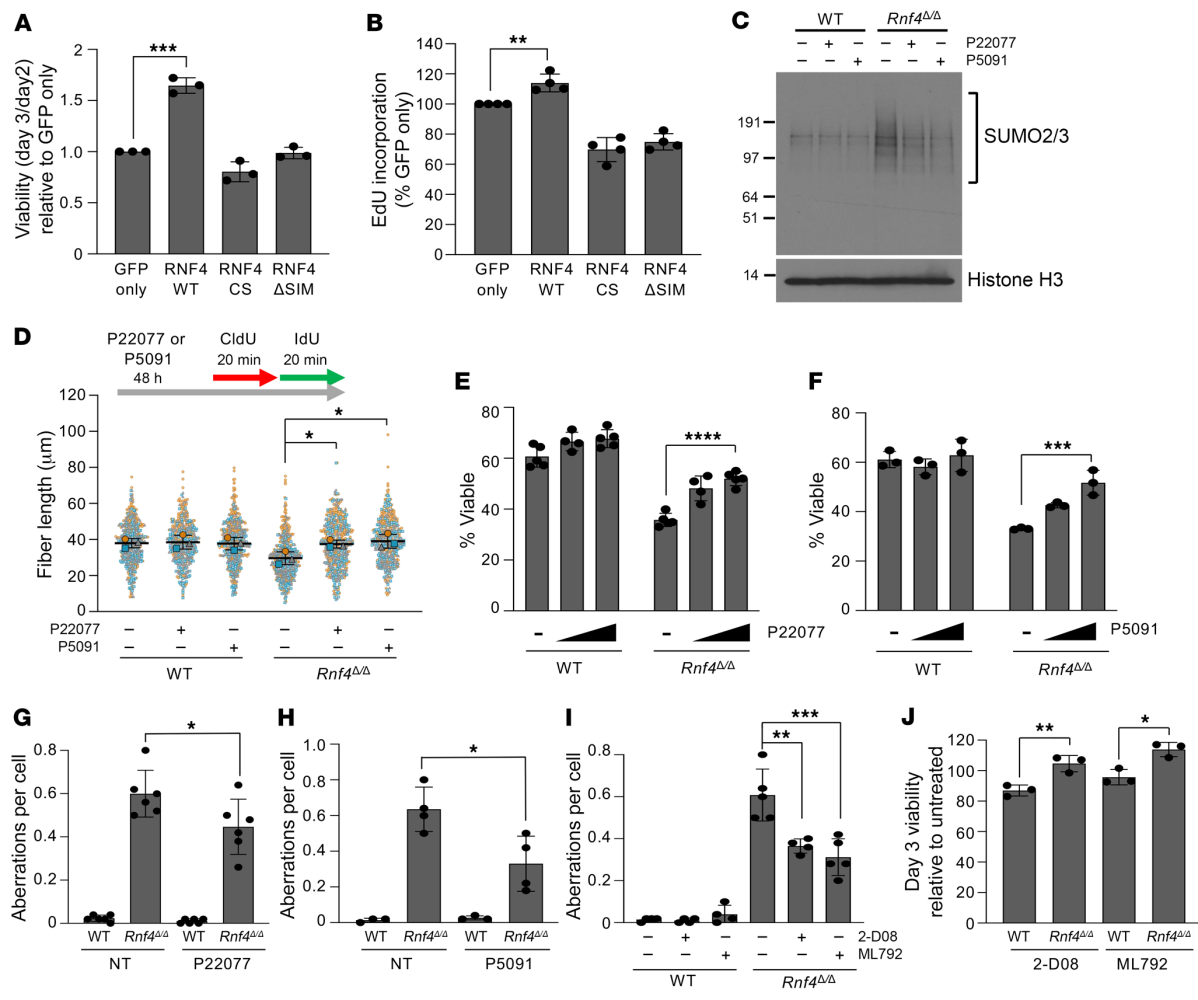
Phenotypes of *Rnf4^Δ/Δ^* cells are caused by accumulation of SUMOylated proteins. (**A**) Analysis of viability of *Rnf4^Δ/Δ^* cells transduced with constructs expressing GFP, WT RNF4, a catalytic mutant of RNF4 (RNF4-CS), or RNF4 defective for SUMO binding (RNF4-ΔSIM). (**B**) Quantification of EdU uptake by *Rnf4^Δ/Δ^* cells transduced with GFP or RNF4 cDNAs. (**C**) Western blot of cells grown in vitro for 2 days with or without continual treatment with USP7 inhibitor P22077 (2 μM) or P5091 (1 μM). (**D**) Analysis of nascent DNA tract length in WT and *Rnf4^Δ/Δ^* B cells. Cells were cultured in vitro for 48 hours with or without P22077 (2 μM) or P5091 (1 μM), then incubated with CldU for 20 minutes, followed by IdU for 20 minutes. CldU and IdU tract lengths were analyzed after DNA combing. Mean ± SD of *n* = 3 experiments shown. (**E**) Cell viability after 72 hours of culture with or without P22077 (1 μM, 2 μM). Viable cells were identified based on ability to exclude DAPI. (**F**) As in **E**, using P5091 (0.5 μM, 1 μM). (**G**) Analysis of chromosome aberrations in B cells cultured 48 hours with or without P22077 treatment (2 μM). (**H**) As in **G**, using P5091 (1 μM). (**I**) Analysis of chromosome aberrations in B cells cultured 48 hours with or without continuous treatment with SUMO inhibitor 2-D08 (40 μM) or ML-792 (20 nM). (**J**) Cell viability measured by quantification of DAPI-negative cells after 72 hours of culture with or without 2-D08 (40 μM) or ML-792 (20 nM). Error bars in **A**, **B**, and **E**–**J** show SD of the mean. *P* values were calculated with unpaired 2-tailed *t* test (**A**, **B**, and **J**), 1-way ANOVA with Dunnett’s multiple-comparison test (**D** and **I**), 2-way ANOVA with Dunnett’s multiple-comparison test (**E** and **F**), and 1-way ANOVA with Tukey’s multiple-comparison test (**G** and **H**). *P < 0.05; **P < 0.01; ***P < 0.001; ****P < 0.0001.

Transduction of *Rnf4^Δ/Δ^* B cells with a construct expressing WT RNF4 resulted in a rescue of cell viability and EdU uptake (Figure 3, A and B). This effect was not seen with RNF4 constructs containing mutations in the E3 ubiquitin ligase active site, or in the SUMO-interaction motifs. The STUbL activity of RNF4 is therefore essential for normal cell viability. To test whether the defective DNA replication, genomic instability, and cell death observed in *Rnf4^Δ/Δ^* cells are specifically caused by the accumulation of high–molecular weight SUMOylated proteins, we used inhibitors of ubiquitin-specific protease 7 (USP7), a ubiquitin isopeptidase that removes ubiquitin from SUMOylated substrates (30). Short-term treatment with USP7 inhibitor produced an increase in the amount of ubiquitination of SUMOylated proteins (Supplemental Figure 3), indicating that other cellular E3 ubiquitin ligases target SUMOylated proteins at some level in the absence of RNF4. Continuous treatment with the USP7 inhibitor P22077 or P5091 substantially reversed the accumulation of SUMOylated proteins in *Rnf4^Δ/Δ^* cells, presumably by stabilizing ubiquitin chains on those proteins to enable proteasomal targeting (Figure 3C). This reduction in the quantity of SUMOylated proteins was associated with a rescue in the speed of replication forks in *Rnf4^Δ/Δ^* cells (Figure 3D), reduced cell death (Figure 3, E and F), and a reduction in the frequency of chromosome aberrations (Figure 3, G and H). These results suggest that the slow replication forks, genomic instability, and increased cell death observed in *Rnf4^Δ/Δ^* cells arise because of a failure to properly degrade SUMOylated proteins. Inhibition of SUMOylation by 2-D08, which blocks transfer of SUMO from UBC9 to potential substrates (31), or by ML-792, which inhibits the SUMO-activating enzyme SAE1/2 (32), also produced a partial rescue of chromosome instability and cell death in *Rnf4^Δ/Δ^* cells (Figure 3, I and J), showing that accumulation of poly-SUMOylated proteins in *Rnf4^Δ/Δ^* cells is causative of reduced cell viability.

### Rnf4 deletion leads to defective recruitment of proteins required for replication stress responses.

**Figure 4:**
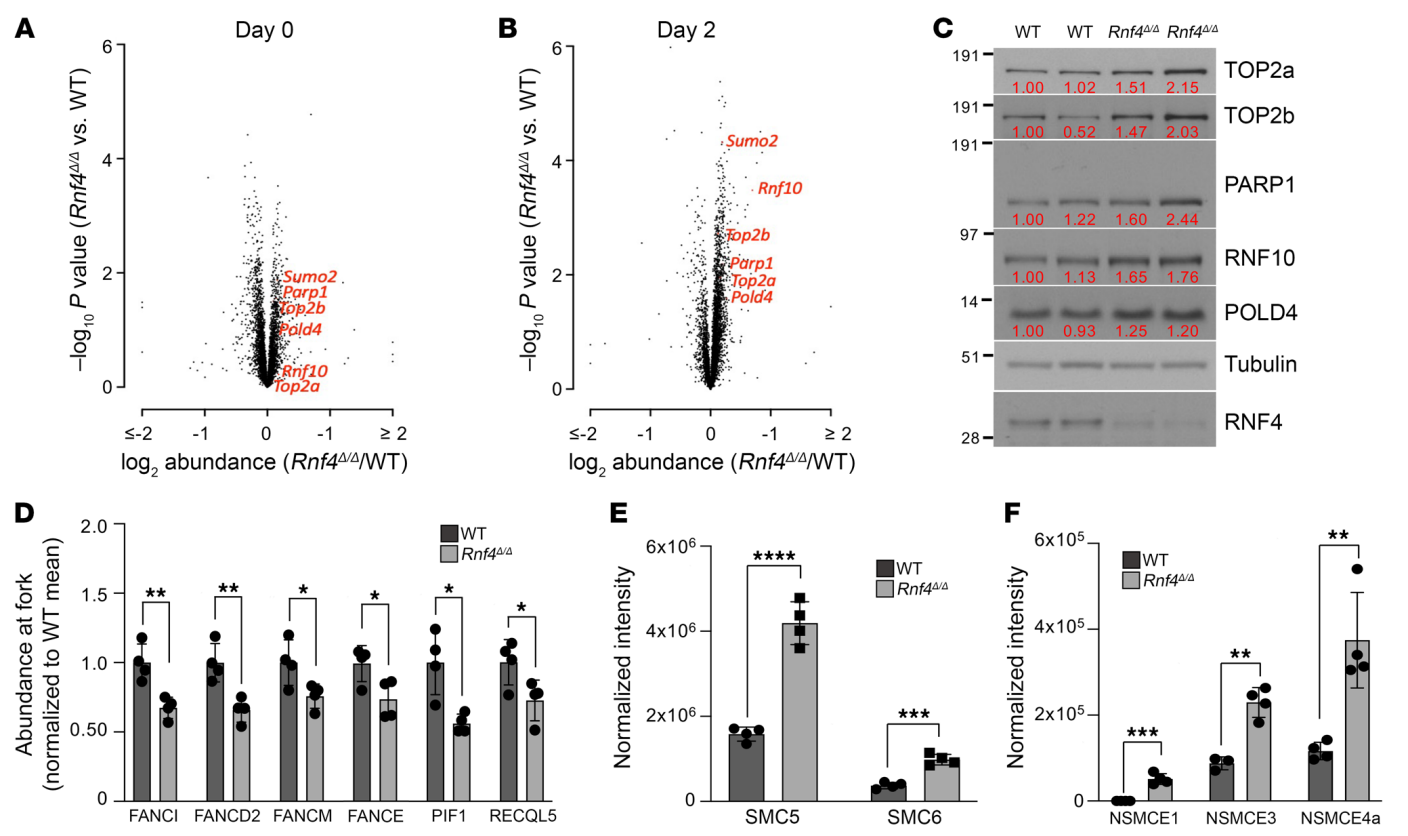
RNF4 deficiency leads to reduced accumulation of proteins needed for replication stress responses at replication forks. (**A**) Volcano plot showing protein abundance in resting *Rnf4^Δ/Δ^* B cells relative to WT controls. Each data point represents the average of 4 samples. Six proteins that showed increased abundance after B cell activation are labeled. (**B**) Volcano plot of B cells after 48 hours of in vitro culture with cell proliferation induced by addition of LPS. (**C**) Western blot showing expression of proteins in WT and *Rnf4^Δ/Δ^* B cells after 48 hours of culture. Two samples are shown in each case. Figures in red indicate band intensities measured with ImageJ. (**D**) Abundance of proteins required for replication stress responses at replication forks, measured by iPOND-MS, in WT and *Rnf4^Δ/Δ^* B cells. (**E**) Increase in abundance of SUMOylated SMC5 and SMC6 in *Rnf4^Δ/Δ^* cells, measured by mass spectrometry after SUMO2/3 immunoprecipitation of chromatin from WT and *Rnf4^Δ/Δ^* cells. (**F**) Increase in abundance of SUMOylated non-SMC element (NSMCE) subunits of the SMC5/6 complex in chromatin of *Rnf4^Δ/Δ^* cells. Error bars in **D**–**F** show SD of the mean of *n* = 4 replicates. *P* values were calculated by unpaired 2-tailed *t* test. *P* < 0.05 was considered significant. *P < 0.05; **P < 0.01; ***P < 0.001; ****P < 0.0001.

To identify changes in cellular protein expression that might account for the genomic instability and reduced viability of *Rnf4^Δ/Δ^* cells, we performed quantitative isobaric labeling mass spectrometry with resting B cells (day 0) and cells that were stimulated to divide in vitro for 48 hours (day 2). In resting B cells, there was no substantial difference in protein expression between WT and *Rnf4^Δ/Δ^* cells (Figure 4A and Supplemental Table 2). After activation of B cells and the initiation of cell division, there were substantial changes in protein abundance in both the WT and *Rnf4^Δ/Δ^* samples (Figure 4B). Activated *Rnf4^Δ/Δ^* B cells showed a modest but statistically significant increase in the abundance of 455 proteins encompassing a broad range of protein classes (Supplemental Table 3 and Supplemental Figure 4, A and B). Increases in the abundance of several of these proteins were validated by Western blotting (Figure 4C). The broad changes in protein expression in *Rnf4^Δ/Δ^* B cells are consistent with previous proteomic studies, which revealed that RNF4 targets a wide range of substrates (33, 34).

As *Rnf4^Δ/Δ^* cells appear to have specific problems with DNA replication, we next used isolation of proteins on nascent DNA (iPOND) (35) to identify proteins with altered abundance at replication forks (Supplemental Table 4). Protein enrichment analysis on candidate proteins that showed substantially altered abundance at replication forks in *Rnf4^Δ/Δ^* cells revealed a cluster of proteins around FANCD2 (Supplemental Figure 4C), including Fanconi anemia family proteins with known functions in ensuring stable replication (36). Several of these proteins showed reduced abundance at replication forks in *Rnf4^Δ/Δ^* cells (Figure 4D), potentially accounting for the reduced replication efficiency seen in the absence of RNF4. The helicases PIF1 and RECQL5, which are required for DNA replication through difficult substrates (37, 38), also showed diminished abundance in iPOND samples from RNF4-deficient cells. These results demonstrate that *Rnf4^Δ/Δ^* cells show a deficiency in recruitment of proteins necessary for replication fork stability and progression.

*Rnf4^Δ/Δ^* cells accumulate poly-SUMOylated proteins in chromatin (Supplemental Figure 4D). We performed immunoprecipitation of SUMO2/3 on chromatin samples from WT and *Rnf4^Δ/Δ^* cells, to attempt to identify hyper-SUMOylated chromatin proteins in *Rnf4^Δ/Δ^* samples, which might contribute to defective replication fork progression. Mass spectrometry revealed a set of proteins with 2 clusters including proteins involved in regulation of SUMOylation, which are known targets of RNF4 (34), and several members of the SMC5/6 complex (Supplemental Table 5 and Supplemental Figure 4, E and F). The SMC5/6 complex is regulated by SUMOylation, and contributes to replication fork stability and regulation of recombination (39). SUMOylated SMC5 and SMC6 were significantly enriched in chromatin from *Rnf4^Δ/Δ^* cells relative to WT (Figure 4E). Three non-SMC element (NSMCE) subunits of the SMC5/6 complex were additionally present at increased abundance in SUMO2/3-immunoprecipitated proteins (Figure 4F). The SMC5/6 complex therefore shows significant hyper-SUMOylation in chromatin of *Rnf4^Δ/Δ^* cells, which may contribute to deficiencies in responding to replication stress.

### Cell death and genomic instability in RNF4-deficient cells are dependent on RAD51.

**Figure 5:**
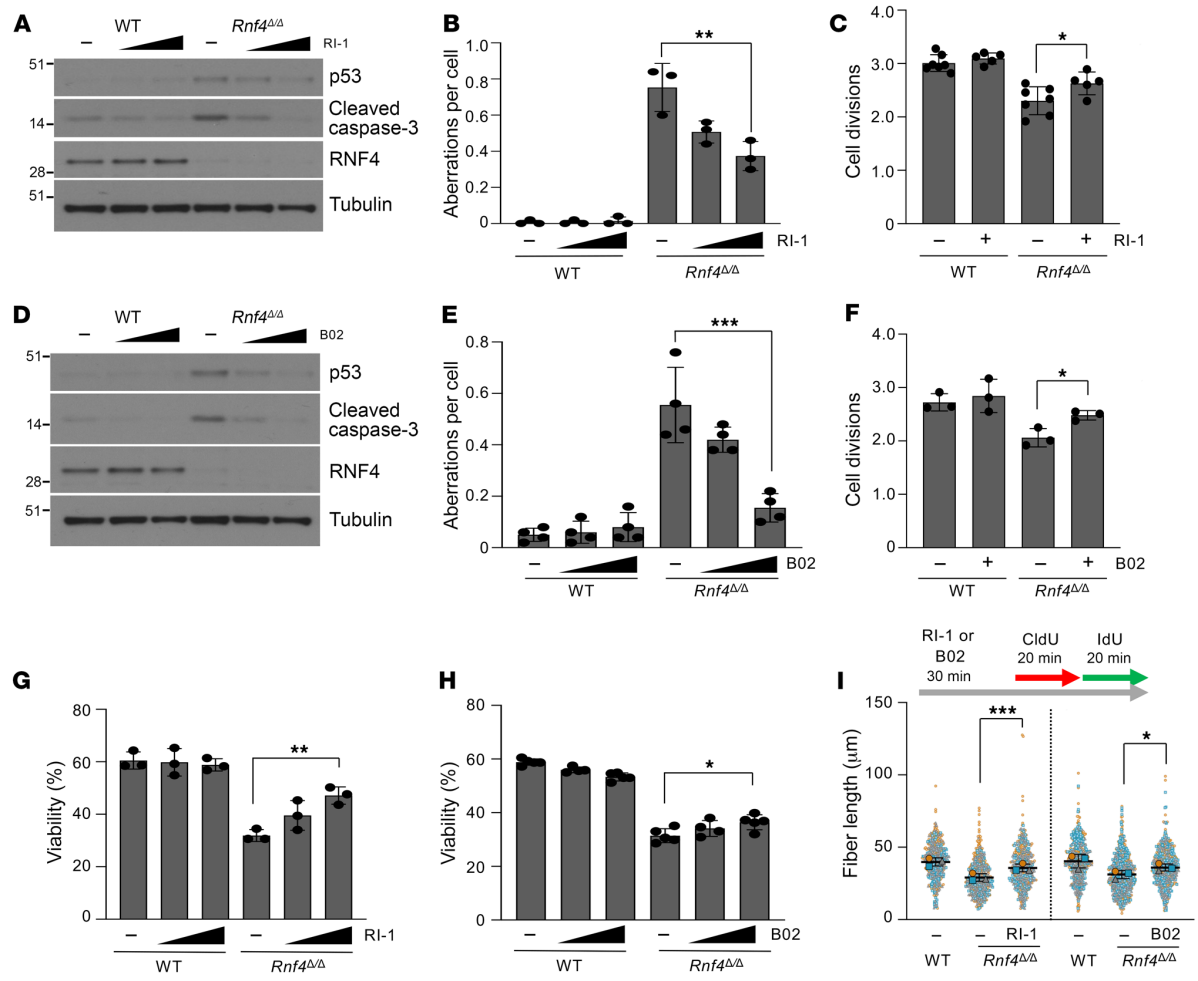
Genomic instability and cell death in RNF4-deficient cells are dependent on RAD51. (**A**) Western blot analysis of WT and *Rnf4^Δ/Δ^* B cells cultured in vitro for 72 hours with mock treatment (–) or with the RAD51 inhibitor RI-1 (5 μM, 10 μM). (**B**) Analysis of chromosome aberrations in day 2 cultured B cells with or without RI-1 treatment (5 μM, 10 μM). (**C**) Analysis of CFSE FACS to measure cell proliferation during 72 hours of growth with or without RI-1 treatment (5 μM). (**D**) As in **A**, with the RAD51 inhibitor B02 (5 μM, 10 μM). (**E**) As in **B**, with B02 (5 μM, 10 μM). (**F**) As in **C**, with B02 (5 μM). (**G**) Viability of B cells, measured by DAPI exclusion, after treatment with RI-1 (5 μM, 10 μM) on day 3. (**H**) As in **G**, with B02 (2.5 μM, 5 μM). (**I**) Measurement of newly replicated DNA by DNA combing after pulsing of cells with CldU and IdU with or without RI-1 or B02 (10 μM in each case). Mean ± SD of *n* = 3 experiments shown. Error bars in **B**, **C**, and **E**–**H** represent SD of the mean. *P* values were calculated by 1-way ANOVA with Dunnett’s multiple-comparison test (**B**, **E**, **G**, and **H**), unpaired 2-tailed *t* test (**C** and **F**), and 1-way ANOVA with Tukey’s multiple-comparison test (**I**). *P* < 0.05 was considered significant. *P < 0.05; **P < 0.01; ***P < 0.001

The SMC5/6 complex is known to prevent the accumulation of toxic recombination intermediates that appear at stalled replication forks (39, 40). We observed that knockdown of *RNF4* using multiple different siRNAs in U2OS EJ-DR reporter cells caused an increase in the rate of HR-mediated repair of DNA double-strand breaks (Supplemental Figure 5A). Ionizing radiation–induced foci of RAD51 formed normally in the nuclei of *Rnf4^Δ/Δ^* B cells (Supplemental Figure 5B). To test whether a potential increase in DNA recombination in RNF4-deficient cells might contribute to apoptosis and defective replication, we used the RAD51 inhibitors RI-1 and B02 (41, 42). At high concentrations, these agents can block formation of ionizing radiation–induced RAD51 foci, and reduce HR efficiency in reporter assays (Supplemental Figure 5, C and D). Treatment with lower doses did not have a measurable impact on the cell cycle of WT cells (Supplemental Figure 5E), but substantially improved the growth of RNF4-deficient B cells (Figure 5, A–H). RAD51 inhibition significantly reduced the p53 activation and caspase-3 cleavage normally observed in *Rnf4^Δ/Δ^* cells, reduced the frequency of chromosome aberrations, and improved cell proliferation and viability. Improved growth of *Rnf4^Δ/Δ^* cells after RAD51 inhibition was associated with a rescue of replication fork velocity (Figure 5I). RAD51 did not show an increased abundance at replication forks in iPOND data sets from *Rnf4^Δ/Δ^* cells, however, nor was the overall abundance or level of ubiquitination of RAD51 substantially changed (Supplemental Figure 5, F–H). RAD51-mediated toxicity may arise because of dysregulation of some other RNF4 target, as USP7 inhibition also reduced the hyper-recombinogenic phenotype of siRNF4-treated cells (Supplemental Figure 5I). Targeting other proteins involved in HR did not achieve a similar rescue of the phenotypes of RNF4 deficiency as was achieved with RAD51 inhibition. In particular, deletion of either *Brca1* or *Brca2* did not result in an improvement in the proliferation, viability, or genomic integrity of *Rnf4^Δ/Δ^* cells (Supplemental Figure 6, A–F).

### Deletion of Rnf4 delays tumor formation in a c-myc cancer model.

**Figure 6:**
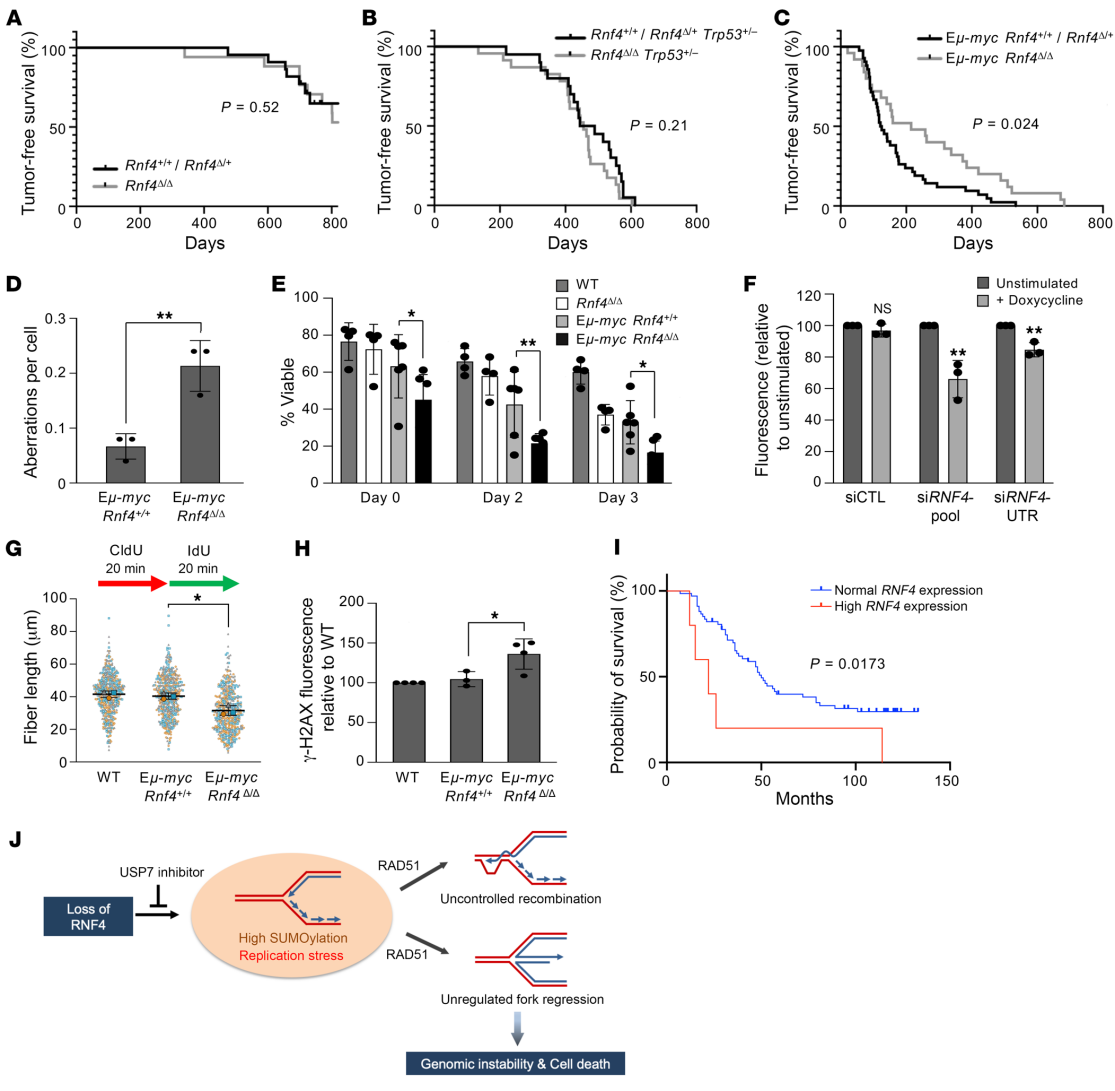
RNF4 deficiency extends tumor-free lifespan of E*μ-myc–*transgenic mice. (**A**) Tumor-free survival of *Rnf4^+/+^* *CD19-Cre* or *Rnf4^fl/+^* *CD19-Cre* (*n* = 38), and *Rnf4^fl/fl^* *CD19-Cre* (*n* = 44). (**B**) Tumor-free survival of *Rnf4^+/+^* *Trp53^+/–^* *CD19-Cre* or *Rnf4^fl/+^* *Trp53^+/–^* *CD19-Cre* (*n* = 30), and *Rnf4^fl/fl^* *Trp53^+/–^* *CD19-Cre* (*n* = 30). (**C**) Tumor-free survival of E*μ-myc* *Rnf4^+/+^ CD19-Cre* or E*μ-myc* *Rnf4^fl/+^* *CD19-Cre* (*n* = 47), and E*μ-myc* *Rnf4^fl/fl^* *CD19-Cre* (*n* = 27). (**D**) Analysis of chromosome aberrations of B cells of the indicated genotypes after 2 days in culture. (**E**) Analysis of cell viability of B cells of the indicated genotypes during 72 hours of in vitro growth. (**F**) Growth of U2OS-iMYC cells after doxycycline-induced expression of c-*myc*, measured by quantification of CellTiter-Glo fluorescence. *P* values show difference between the means of unstimulated and stimulated cells. (**G**) Analysis of replication fork velocity measured by DNA combing. Total CldU plus IdU tract length is shown. Mean ± SD of *n* = 3 experiments shown. (**H**) Intensity of γ-H2AX staining in S-phase (EdU^+^) cells. (**I**) Survival of pediatric B cell acute lymphoblastic leukemia patients with tumors expressing either normal or increased levels of *RNF4*. (**J**) Model for steps leading to cell death in RNF4-deficient cells. Absence of RNF4 activity causes increased abundance of SUMOylated proteins in chromatin, leading to replication stress and accumulation of nonproductive intermediates, dependent on RAD51, which prevents successful completion of DNA replication and further cell proliferation. Error bars in **D**–**F** and **H** show SD of the mean. *P* values were calculated by log-rank test (**A**–**C** and **I**), unpaired 2-tailed *t* test (**D** and **F**), 2-way ANOVA with Dunnett’s multiple-comparison test (**E**), and 1-way ANOVA with Tukey’s multiple-comparison test (**G** and **H**). *P* < 0.05 was considered statistically significant. *P < 0.05; **P < 0.01.

As B cells from *Rnf4*-conditional-knockout mice showed substantial genomic instability (Figure 1E), we performed a longitudinal study to determine whether *Rnf4*-conditional-knockout mice are at increased risk of cancer. Despite elevated genomic instability in *Rnf4^Δ/Δ^* B cells grown in culture, there was no difference in tumor susceptibility caused by deletion of *Rnf4* in the B cell lineage (Figure 6A). The overall survival of conditional-knockout and control mice also showed no significant difference (Supplemental Figure 7A). Tumor susceptibility in *Brca1*- and *Brca2-*deficient mice is more apparent in a p53-hemizygous background (43–45). We therefore crossed *Rnf4*-conditional-knockout mice to a *Trp53^+/–^* background, to test whether loss of RNF4 causes tumor outgrowth in cells lacking normal expression of p53. We observed that *Rnf4* deficiency did not accelerate tumor formation, even on a *Trp53^+/–^* background (Figure 6B), or affect the overall survival rate of *Trp53^+/–^* mice (Supplemental Figure 7B). As in the control group, *Rnf4^Δ/Δ^* *Trp53^+/–^* animals developed tumors characteristic of p53 loss of function, with a large proportion of thymomas (46). Finally, we tested whether *Rnf4* deficiency causes accelerated tumor formation in E*μ-myc* mice, a transgenic model for overexpression of the oncogene c-*myc* in B cells (47). As expected, mice carrying the E*μ-myc* transgene had a very high incidence of B cell lymphoma with a median survival rate of 123 days (Figure 6C). In contrast, mice expressing E*μ-myc* with conditional deletion of *Rnf4* in the B cell lineage showed a significant delay in tumor formation, with a median tumor-free survival of 215 days. The overall lifespan of E*μ-myc Rnf4^Δ/Δ^* mice showed an equivalent extension (Supplemental Figure 7C). These studies show that *Rnf4* deletion does not predispose mice to tumor formation, and loss of *Rnf4* delays the onset of tumors in cells with c*-myc* overexpression.

We considered whether defects in DNA replication might limit the growth of incipient *myc*-dependent malignancies in mice carrying the E*μ-myc* transgene. Consistent with the well-known ability of c-*myc* to induce cellular proliferation, we observed EdU uptake by freshly isolated E*μ-myc* *Rnf4^+/+^* and E*μ-myc Rnf4^Δ/Δ^* B cells, whereas WT and *Rnf4^Δ/Δ^* B cells showed almost no EdU incorporation (Supplemental Figure 7D). A proportion of E*μ-myc* splenic B cells are therefore dividing, even in the absence of any exogenous mitogenic stimulus. As was the case in *Rnf4^Δ/Δ^* cells, we measured an increased frequency of chromosome aberrations in E*μ-myc Rnf4^Δ/Δ^* B cells relative to E*μ-myc* *Rnf4^+/+^* cells (Figure 6D). E*μ-myc Rnf4^Δ/Δ^* B cells also showed a significantly reduced ability to proliferate in culture as compared with E*μ-myc* *Rnf4^+/+^* cells (Figure 6E). We tested the effect of knocking down RNF4 on the growth of U2OS cells with a stably integrated, doxycycline-inducible c-*myc* construct (48). After knockdown of *RNF4*, these U2OS-iMYC cells showed significantly reduced proliferation compared with cells transfected with control siRNA (Figure 6F). In E*μ-myc*–transgenic cells, deletion of *Rnf4* also led to reduced replication fork velocity, higher levels of γ-H2AX in S phase, and an increased number of 53BP1 G_1_ nuclear bodies (Figure 6, G and H, and Supplemental Figure 7E). These results are consistent with the concept that the increased propensity of *myc*-transgenic cells to proliferate makes them acutely sensitive to loss of RNF4, which is required for normal DNA replication.

To consider whether *RNF4* expression might impact the growth of tumors in humans, we analyzed survival of B cell acute lymphoblastic leukemia patients studied as part of the National Cancer Institute TARGET Phase II initiative. Patients with tumors expressing high levels of *RNF4* had significantly worse survival outcomes than those with tumors expressing normal levels of *RNF4* (Figure 6I). Elevated *RNF4* expression is a feature of several tumor types in The Cancer Genome Atlas (TCGA) data sets (Supplemental Figure 7F), and high *RNF4* expression correlates with poor survival in multiple tumor types, including breast adenocarcinoma and lung adenocarcinoma (Supplemental Figure 7, G and H). These findings suggest that RNF4 may have a general role in supporting tumor outgrowth, by facilitating DNA replication in malignant cells.

## Discussion

Our observation that deletion of *Rnf4* does not lead to malignancy in B lymphocytes, even in the absence of p53, is potentially surprising considering the high rate of genomic instability observed in RNF4-deficient cells. Mice with deletions in known DNA repair genes, such as *Brca1* or *Brca2*, show tumor susceptibility, especially on a p53-deficient background (43, 45). The rate of chromosome instability in untreated *Rnf4^Δ/Δ^* B cells is significantly higher than that in *Brca1^Δ11/Δ11^* B cells (Figure 2A), and yet no tumor susceptibility was observed in *Rnf4*-conditional-knockout mice. This outcome is potentially similar to the situation with cells lacking RAD51, the central mediator of HR (49). Repression of *RAD51* expression leads to accumulation of chromatid and chromosome breaks (50), but *RAD51* mutations do not correlate strongly with cancer incidence (51). Mouse knockout studies have shown that loss of RAD51 causes a complete block in cellular proliferation (52, 53); therefore it is likely that cancer cells cannot survive in the absence of RAD51. Based on our results, we propose that loss of RNF4 has a similar effect, in that knockout cells show chromosome instability but are also unable to replicate and divide normally, leading to cell death instead of malignancy.

We find that the block on cell growth caused by conditional inactivation of *Rnf4* is so strong as to limit the outgrowth of c-*myc*–dependent lymphomas. Tumors driven by *myc* activity are dependent on SUMOylation, and SUMO regulators are upregulated by *myc* overexpression (54). Our work further underscores the importance of SUMO regulation for tumor cell growth, and reveals the importance of RNF4 in this pathway. The dependence of incipient tumor cells from E*μ-myc* mice on RNF4 for survival and tumor outgrowth is an example of “non-oncogene addiction” (55). Increased proliferative signaling arising from *myc* overexpression leads to rapid DNA replication, which can only be sustained if RNF4 is available to ensure appropriate ubiquitination in replicating chromatin domains. A similar effect is seen with loss of ATR and CHK1, which signal the presence of DNA replication stress, including that induced by oncogene expression (29). CHK1 inhibition compromises the growth of tumors overexpressing oncogenic Ras or Myc, and tumor formation is significantly reduced in E*μ-myc* mice expressing mutant ATR (56, 57).

Several studies in mammalian cells have supported a role for RNF4 in regulation of DNA replication. In ATR-deficient cells, RNF4 promotes SLX4-mediated cleavage of replication forks, leading to fork collapse and the appearance of DNA double-strand breaks (58). RNF4 knockdown also reduces the ability of replication forks to restart after persistent replication stress, based on the ability of RNF4 to ubiquitinate BLM helicase (59). Another recent study identified RNF4 as the factor responsible for removal of the ZAPP-TOP2a-PICH complex from replication forks (33, 60). This finding is consistent with earlier studies, which showed that RNF4 targets trapped TOP1- and TOP2-cleavage complexes for proteasomal degradation (61, 62). Several studies have identified PARP1 as a target of the E3 ubiquitin ligase activity of RNF4 (63, 64). PARP1 is active at replication forks, and the cytotoxic effect of PARP inhibitors is partially dependent on their ability to trap PARP1 on chromatin, creating a barrier to replication fork progression (65). Although we observed modest increases in the abundance of PARP1, TOP2a, and TOP2b in whole-cell extracts from *Rnf4^Δ/Δ^* cells (Figure 4, B and C), we did not observe significant changes in these factors in iPOND–mass spectrometry (MS) studies of proteins at active replication forks. We did find reductions in the abundance of FANCD2, FANCI, and FANCM at replication forks in RNF4-deficient cells (Figure 4D). Each of these factors is known to function in ensuring replication fork progression and stability (66–68). The helicases PIF1 and RECQL5, which are required for replication through challenging substrates such as G quadruplexes and transcription-replication conflict sites, are also present at reduced abundance at replication forks of *Rnf4^Δ/Δ^* cells (37, 38).

Work from the Fernandez-Capetillo laboratory has supported a model in which chromatin around active DNA replication forks is marked by high levels of SUMOylation and low levels of ubiquitination (69). According to this model, chromatin outside of active replicating regions is marked by lower levels of SUMOylation, and high levels of ubiquitination. Disruption of the normal balance of ubiquitin- and SUMO-conjugated proteins in replicating chromatin domains leads to a reduction in replication fork progression (30). We find that SUMOylation is broadly increased in chromatin in *Rnf4^Δ/Δ^* cells (Supplemental Figure 4D) and this increase correlates with reduced replication fork velocity and cell survival. In particular, we identified 5 of 8 components of the mammalian SMC5/6 complex as hyper-SUMOylated chromatin constituents in *Rnf4^Δ/Δ^* cells (Figure 4, E and F). The SMC5/6 complex is a multiprotein assembly that encircles DNA and facilitates loop extrusion (70). The SMC5/6 complex facilitates DNA replication by helping to relieve chromosome-wide superhelical tension that arises around active replisomes (71). This process involves the prevention of intertwining of sister chromatid molecules behind the replication fork. Depletion of SMC5/6 in yeast causes a block in replication at the ribosomal DNA array (39). The SMC5/6 complex furthermore physically interacts with FANCI and FANCD2 in mammalian cells (72); therefore defects in the regulation of SMC5/6 may account for the reduced accumulation of FANCI and FANCD2 at replication forks in *Rnf4^Δ/Δ^* cells. The processivity of replication forks in budding yeast is dependent on the SMC5/6 complex, which limits MPH1-mediated fork regression, and promotes error-free bypass of DNA lesions (73, 74). The ability of SMC5 to promote lesion bypass is dependent on SUMOylation; therefore it is an attractive candidate for regulation by RNF4.

Toxic recombination intermediates accumulate in cells with deficiencies in the SMC5/6 complex (39, 40, 75). The cytotoxic effects of RNF4 deletion are notably rescued by RAD51 inhibitors, indicating that RAD51-dependent recombination or fork regression is causative of cell death in the absence of RNF4. It is possible that toxic RAD51-dependent structures arise because of a failure to adequately regulate or localize the SMC5/6 complex in RNF4-deficient cells. Mutations in *slx5*, the yeast homolog of RNF4, are known to produce gross chromosomal rearrangements caused by aberrant recombination between homeologous sequences (76). Although the importance and details of specific mechanisms for RNF4-dependent regulation of DNA replication remain to be resolved, it is clear that RNF4 is required for efficient replication, and may therefore represent an attractive target for cancer cases driven by *myc*, or other oncogenes that drive rapid cellular proliferation.

## Methods

### Sex as a biological variable.

Sex was not considered as a biological variable in this study. Both male and female mice were used for all studies.

### Generation of Rnf4-conditional-knockout mice.

Cas9:sgRNA ribonucleoprotein complexes were prepared by incubation of eSpCas9 (MilliporeSigma) with sgRNA and then addition of single-stranded oligodeoxynucleotides, which contained homology arms flanking *loxP* sequences. This mixture was microinjected into C57BL/6J embryos. The 5′ *loxP* sgRNA spacer sequence was CAATCACTTGCCTTATAAGA (MilliporeSigma), and the 5′ *loxP* oligonucleotide donor sequence was 5′-CAAGATCTTAGGCAGGAAGATGGATGCCTTTGGTCAGACATGTTGGTTTTACTGAAAATCTCTGCCATTGTACCATGGGCTGAGCCTTCT**ATAACTTCGTATAGCATACATTATACGAAGTTAT**TATAAGGCAAGTGATTGGCCTCAGTGCCTGGACAGGACCTTCTGAG-3′ (*loxP* in bold). For the 3′ *loxP*, the sgRNA spacer sequence was TTGGGTTCAGCTGTCTGCTC, and the 3′ *loxP* donor oligonucleotide sequence was 5′-ACAAGTATCTTCCAAGCAGCTAGGTAGGCATATTTGGAATACAGGGTTGGGTTCAGCTGTCTGa**ATAACTTCGTATAGCATACATTATACGAAGTTAT**CTCAGGATAGACCAATTGACAGACAGACAGGGCCATACATACTGACCAGGGTGTGCTTTTCGCT-3′ (*loxP* in bold, the base in lowercase was added to create an XmnI restriction site for screening). Three founders were determined to have both *loxP* sites correctly inserted flanking *Rnf4* exon 6. A single line used for the study was backcrossed 4 times to C56BL/6.

### Cell culture.

Primary B cells were isolated from mouse spleen and activated for in vitro culture with lipopolysaccharide and interleukin-4 (IL-4) as previously described (77). U2OS doxycycline-inducible Myc-GFP (U2OS-iMYC) cells (48) were seeded in triplicate with or without 0.5 μg/mL doxycycline treatment in a 24-well plate after siRNA transfection. Eight days after seeding, proliferation was assayed using CellTiter-Glo Luminescent Cell Viability assay (G7572, Promega) according to the manufacturer’s instructions. PLAT-GP cells (Cell Biolabs) were cultured in DMEM containing 10% FBS and 0.5% penicillin-streptomycin. For the in vivo SUMOylation assay, FLAG-RAD51 plasmid was transfected into siRNA-treated U2OS cells using Lipofectamine 2000. Cells were processed 24 hours after transfection as previously described (78).

### Antibodies, chemicals, and plasmids.

Custom anti-RNF4 antibody was generated as previously described (79). Commercial antibodies used in this study included: SUMO2/3 (ab3742, Abcam), p53 (2524, Cell Signaling Technology [CST]), cleaved caspase-3 (9661, CST), tubulin (T8238, Sigma-Aldrich), KAP1–p-S824 (A300-767A, Bethyl), CHK1–p-S345 (2341, CST), CHK1 (sc-8408, Santa Cruz Biotechnology), γ-H2AX (05-636, Millipore), γ-H2AX–Alexa Fluor 488 (20304S, CST), histone H3 (ab1791, Abcam), TOP2a (sc-365916, Santa Cruz Biotechnology), TOP2b (MAB6348, Novus), PARP1 (9542, CST), RNF10 (16936-1-AP, Proteintech). Antibody specific for mouse POLD4 was provided by Marietta Lee (New York Medical College, Valhalla, New York, USA). The following chemicals were used: olaparib (S1060, Selleckchem), hydroxyurea (H8627, Sigma-Aldrich), aphidicolin (A4487, Sigma-Aldrich), cisplatin (S1166, Selleckchem), MMS (129925, Sigma-Aldrich), gemcitabine (G6423, Sigma-Aldrich), P22077 (662142, Sigma-Aldrich), P5091 (2277, Biovision), 2-D08 (SML1052, Sigma-Aldrich), ML-792 (407886, MedKoo), RI-1 (SML1294, Sigma-Aldrich), and B02 (SML0364, Sigma-Aldrich). For plasmids, human *RAD51* cDNA was inserted into pRK5-FLAG, and human *RNF4* cDNA was inserted into pMX-PIE-IRES-EGFP. Amino acid substitutions for *RNF4*-CS and *RNF4*-ΔSIM were performed as previously described (78, 80).

### Chromatin fractionation and immunoprecipitation.

For chromatin fractionation, harvested cells were gently resuspended in buffer A (10 mM HEPES [pH 7.9], 1.5 mM MgCl_2_, 10 mM KCl, 0.05% NP-40, 0.5 mM DTT, 10 mM *N*-ethylmaleimide [NEM], 50 mM glycerol-2-phosphate, supplemented with protease inhibitor cocktail [Roche, catalog 11836153001]) and incubated for 10 minutes on ice. Cell nuclei were collected by centrifugation (5 minutes, 1,300*g*, 4°C) and incubated in buffer B (3 mM EDTA, 0.2 mM EGTA, 0.5 mM DTT, 10 mM NEM, 50 mM glycerol-2-phosphate, and protease inhibitor cocktail) for 30 minutes. The chromatin component was collected by centrifugation (4 minutes, 1,700*g*, 4°C) and lysed with buffer C (20 mM HEPES [pH 7.9], 500 mM NaCl, 1.5 mM MgCl_2_, 0.2 mM EDTA, 0.5 mM DTT, 125 U/mL benzonase, 10 mM NEM, 50 mM glycerol-2-phosphate, and protease inhibitor cocktail). Lysates were incubated for 5 minutes at 37°C on a shaking heat block followed by 20 minutes of incubation on ice. After centrifugation at 16,000*g* for 20 minutes, supernatant containing chromatin-bound protein was collected. Anti-SUMO2/3 immunoprecipitation of chromatin fractions was performed after 10-fold dilution of chromatin samples in dilution buffer (50 mM Tris-HCl [pH 7.5], 150 mM NaCl, 0.5% NP-40, 10 mM NEM, 50 mM glycerol-2-phosphate, and protease inhibitor cocktail), with protein G–agarose used for collection.

### Double-strand break repair reporter assay.

The EJ-DR assay was performed as previously described (81). To induce incorporated I-Sce1, Shield1 (632189, Clontech) and triamcinolone (T6510, Sigma-Aldrich) ligands were added to the media for 24 hours. Where necessary, RAD51 inhibitors were added together with the Shield1 and triamcinolone. Nonhomologous end-joining and HR repair activity was assessed by quantification of DsRed- and GFP-positive cells on a BD FACSCalibur system and analyzed on FlowJo (Tree Star) 48 hours after induction.

### siRNA depletion.

Cells were transfected with siRNA using Lipofectamine RNAiMAX (13778, Invitrogen). The following siRNA oligonucleotides were used in this study: MISSION siRNA Universal Negative Control #1 (SIC001, Sigma-Aldrich), siGENOME Human RNF4 siRNA set of 4 (M-006557-03, Dharmacon), siRNF4-1 (D-006557-03, Dharmacon), siRNF4-2 (D-006557-04, Dharmacon), siRNF4-3 (D-006557-05, Dharmacon), siRNF-UTR (GGGCAUGAAAGGUUGAGAA), siGENOME Human BRCA1 siRNA set of 4 (M-003461-02, Dharmacon), siGENOME Human BRCA2 siRNA set of 4 (M-003462-01, Dharmacon).

### Flow cytometry.

For the CFSE proliferation assay, purified B cells were resuspended at 1 × 10^7^ cells/mL in RPMI medium, labeled with 5 μM CFSE for 10 minutes at 37°C, then cultured in RPMI medium with or without IL-4 and LPS for 72 hours. The CFSE fluorescence of unstimulated cells was taken as 100%, and the number of additional cell divisions was calculated according to reduced CFSE fluorescence as the cells divided. Number of cell divisions was calculated as log_2_(median intensity of unstimulated cells / median intensity of indicated cells). For EdU incorporation, B cells were pulsed with 30 μM EdU for 30 minutes, and fixed with ice-cold methanol for 20 minutes. EdU was detected using the Click-iT EdU Alexa Fluor 647 imaging kit. Cells were stained with DAPI to measure DNA content. For flow cytometry analysis of splenocyte populations, mouse splenocytes were treated with ACK lysis buffer for 5 minutes, then blocked with anti-CD16/CD32 antibody for 10 minutes at room temperature and labeled with B220–Alexa Fluor 647 (557683, BD Biosciences) and CD43-PE (12-0431-82, Invitrogen) for 1 hour at 4°C. Cytometry was performed using a BD FACSCalibur or Cytek Aurora with analysis in FlowJo.

### Alkaline comet assay.

Alkaline comet assays were performed using a CometAssay kit (Trevigen) according to the manufacturer’s protocol. Splenic B cells were cultured for 48 hours in vitro and then treated with mock or 100 μM MMS treatment for 3 hours. Images were acquired by Nikon Eclipse E800 epifluorescence microscope and analyzed with OpenComet software.

### DNA combing and DNA fiber analysis.

To measure fork speed, cells were incubated with 20 μM 5-chlorodeoxyuridine (CldU; C6891, Sigma-Aldrich) for 20 minutes, followed by 20 minutes of incubation with 100 μM 5-iododeoxyuridine (IdU; I7125, Sigma-Aldrich). Genomic DNA purification and combing on glass coverslips were performed using materials and a protocol from Genomic Vision. DNA fiber analysis was performed as previously described (82). The DNA was stained with anti-CldU antibody (ab6326, Abcam) and anti-IdU antibody (347580, BD Biosciences) for 1 hour, followed by incubation with secondary antibodies for 45 minutes. Images were acquired by Nikon Eclipse E800 epifluorescence microscope using a ×40 objective and analyzed with ImageJ software (NIH).

### iPOND.

iPOND was performed as previously described (35).

### Retroviral transduction.

To express the human *RNF4* gene in B cells, retroviral vectors were transfected into PLAT-GP cells using Lipofectamine 2000. B cells were cultured in vitro for 36 hours and spinoculated with retroviral supernatants at 1,150*g* for 1.5 hours in the presence of 10 μg/mL Polybrene. After 4 hours at 37°C, supernatants were replaced with normal medium. The percentage of GFP-positive and DAPI-negative B cells was measured with flow cytometry on day 2 and day 3 to check the infection rates and viability.

### Preparation of metaphase spreads and FISH.

Telomere DNA FISH analysis was performed and analyzed as previously described (77). Images were acquired by an AxioImager.Z2 microscope (Zeiss) using a ×63 objective with MetaSystems automatic stage. At least 50 metaphases were analyzed for each experiment.

### Mass spectrometry.

Thirty micrograms of each sample was digested in-gel with trypsin (1:50 ratio, sequencing grade; catalog 90058, Thermo Fisher Scientific) and peptides extracted as described previously (83, 84). Peptides were labeled with Thermo TMTpro 16 Plex (lot WC320807) and prefractionated by high-pH reversed-phase liquid chromatography. MS was conducted using synchronous precursor selection (SPS) MS3 to measure reporter ion intensities on an Orbitrap Eclipse Tribrid Mass Spectrometer (Thermo Fisher Scientific). Instrument settings were: for MS1: Orbitrap resolution 120,000, scan range from 350 to 1,600 *m/z*, automatic gain control (AGC) target 1 × 10^6^, maximum injection time 100 milliseconds; for MS2: top 5 (3 seconds) duty cycle, quadrupole ion trap, AGC 2 × 10^4^, collision energy 35, maximum injection time 35 milliseconds, isolation window set at 0.7; and for MS3: 10 MS2 fragment ions captured as MS3 precursors using isolation waveforms with multiple frequency notches, Orbitrap AGC 1.5 × 10^5^, maximum injection time 54 milliseconds in the higher-energy collisional dissociation (HCD) cell and fragmented with HCD with collision energy of 55% and scanned in the Orbitrap with scan range 100–500 *m/z*. Peak lists were generated by Thermo Proteome Discoverer (v2.1) as MASCOT Generic Format (MGF) files and combined MS2–MS3 peak lists searched against the UniProt mouse database using an in-house version of X! Tandem (GPM Fury) (85). TMT MS3 reporter ion intensities were extracted with correction for isotope channel crossover using custom in-house software and exported to Excel. For each protein, the sum of TMT MS3 intensities for all corresponding peptides was first normalized to the sum of the TMT intensities for each channel to account for differences in labeling efficiency or amount of protein labeled for each channel. The mean of the 4 replicates for each genotype/time point was used to generate ratios for selected comparisons. Proteins were filtered for those with 2 or more assigned spectra. Proteins showing an increase of more than 10% in *Rnf4^Δ/Δ^* cells relative to WT cells with a *P* value less than 0.05 (2-tailed *t* test) were considered for further analysis.

For data-independent acquisition, instrument settings were: MS scan range set to 400–1,200 *m*/*z*, with resolution 12,000, AGC 3 × 10^6^, and ion time set as auto. Eight *m*/*z* windows were used to sequentially isolate (AGC 4 × 10^5^ and ion time auto) and fragment ions in the C-trap using a relative collision energy of 30. MS/MS spectra were recorded with a resolution of 30,000. Data were analyzed using DIA-NN 1.8.1 (86) with default settings for FASTA digest for library-free search/library generation and deep learning–based spectra and retention time prediction using UniProt reference proteome UP000000589 (mouse). Data were normalized using the MaxLFQ algorithm (87), and total normalized precursor intensities for each protein group were filtered for both PEP (an estimate of the posterior error probability for the precursor identification, based on scoring with neural networks) of less than 0.01 and PG.Q (protein group *q* value) of less than 0.01. The *t* tests were calculated as above.

### Statistics.

Statistical tests were performed as described. All *t* tests were 2-tailed. ANOVA tests were 1- or 2-way as specified in figure legends. A *P* value of less than 0.05 was considered statistically significant. Data points on graphs represent results from a minimum of 3 biological replicates. For bee-swarm plots, small data points represent individual values from 3 experiments, separately colored, and large data points indicate the mean of each experiment.

### Analysis of genomic data sets.

Functional categorization and measurements of statistical overrepresentation of protein classes were performed using PANTHER (PantherDB.org). STRING analysis was performed using tools at https://string-db.org cBioPortal (https://www.cbioportal.org) was used to access data on 72 pediatric acute lymphoblastic leukemia patients studied as part of the Therapeutically Applicable Research to Generate Effective Treatments initiative, phs000218 (TARGET Phase II). The data used for this analysis are available at https://portal.gdc.cancer.gov/ Overall survival of patients with tumors with high *RNF4* mRNA expression (RNA-Seq *z* score vs. RSEM ≥ 2.0) was compared with that of patients with *RNF4* mRNA in the unaltered range. cBioPortal was additionally used to retrieve data on survival of patients with above-median and below-median *RNF4* expression in tumor biopsies, based on samples from 1,082 breast adenocarcinoma patients and 501 lung adenocarcinoma patients studied as part of the TCGA PanCancer Atlas project (88). Analysis of RNF4 expression in multiple tumor types was based on TCGA data accessed with the University of Alabama Cancer Data analysis portal (89).

### Study approval.

Vertebrate animal studies were carried out under an animal protocol approved by the Rutgers University IACUC.

### Data availability.

Proteomic data sets were deposited to the MassIVE database with accession numbers MSV000090142, MSV000093660, and MSV000093661. All data used for figures are available in the Supporting Data Values file.

## Author contributions

Experiments were conceived by JH and SFB and performed by JH, except mass spectrometry, which was performed by HZ. The paper was written by SFB.

## Supplementary Material

Supplemental data

Unedited blot and gel images

Supplemental table 1

Supplemental table 2

Supplemental table 3

Supplemental table 4

Supplemental table 5

Supporting data values

## Version 1 03/26/2024

In-Press Preview

## Version 2 05/15/2024

Electronic publication
