# Supplementary material for: RNF4 sustains Myc-driven tumorigenesis by facilitating DNA replication
Source: J Clin Invest. 2024 Mar 26;134(10):e167419. doi: 10.1172/JCI167419 (PMC11093604; doi:10.1172/JCI167419)
Supplement: Supplemental data [file jci-134-167419-s133.pdf]

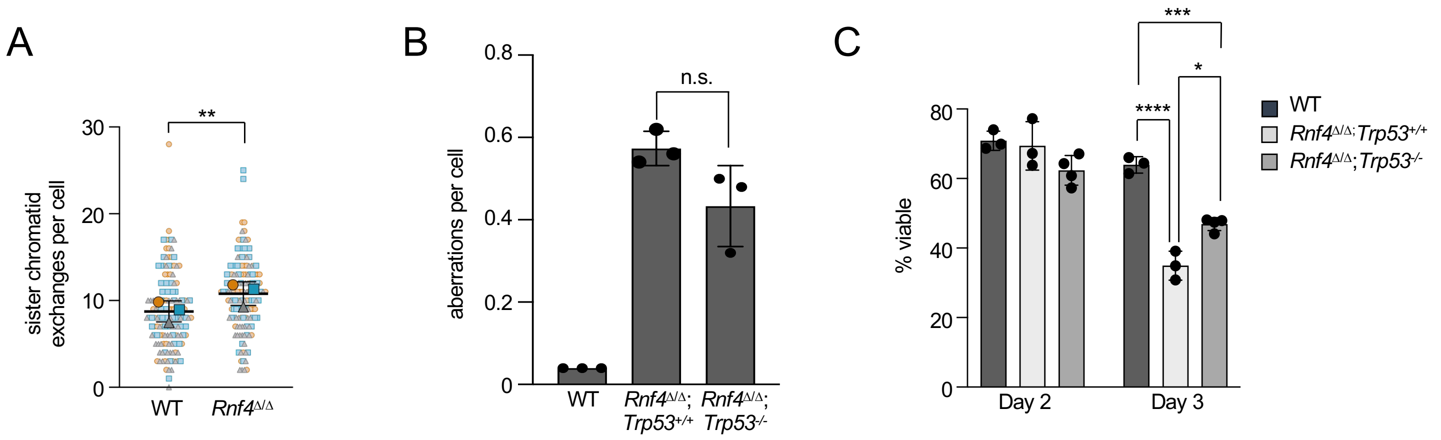

Supplemental Figure 1: (**A**) Quantification of sister chromatid exchanges in WT and *Rnf4*$^{\Delta/\Delta}$ B cells after 48 hrs growth in vitro. Mean ± S.D. of n=3 experiments shown. (**B**) Quantification of metaphase chromosome aberrations from B cells of the indicated genotypes. Error bars show S.D. of the mean. (**C**) Viability of B cells of the indicated genotypes after two or three days in culture. Viable cells were identified by FACS on the basis of PI exclusion. P values were calculated by paired t-test (**A**), unpaired t-test (**B**), and two-way ANOVA with Tukey's multiple comparison test (**C**). $P < 0.05$ was considered significant.

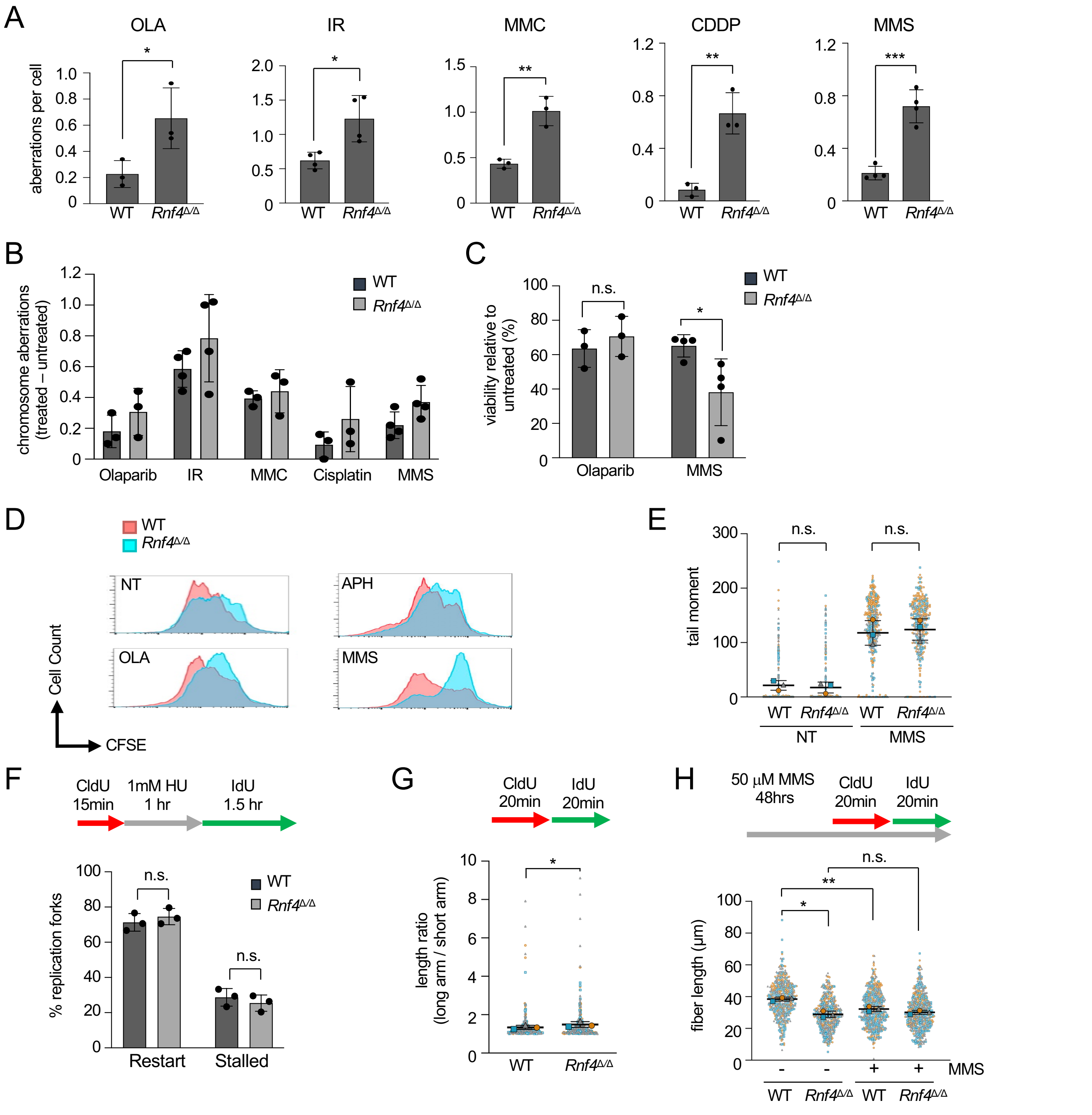

Supplemental Figure 2: (**A**) Chromosome aberrations in WT and *Rnf4*$^{\Delta/\Delta}$ B cells cultured for 36 hrs in vitro followed by overnight treatment with either olaparib (2 µM), ionizing radiation (2 Gy), mitomycin C (MMC, 250 nM), cisplatin (1 µM), or methyl methanesulfonate (MMS, 100 µM). (**B**) Total chromosome aberrations induced by treatments as shown in (A), after subtracting the number of chromosome aberrations present in untreated WT and *Rnf4*$^{\Delta/\Delta}$ B cells. The differences between the means did not meet the threshold for statistical significance ($P < 0.05$) in any case. (**C**) B cell viability after induction of DNA damage. B cells were grown for 72 hrs in culture ± olaparib (1 µM) or MMS (100 µM). Viability was measured by flow cytometry based on DAPI exclusion. (**D**) Representative data for Figure 2B, flow cytometry analysis of CFSE dilution to measure B cell growth in vitro over 72 hrs. (**E**) Alkaline comet assay to measure single-strand DNA breaks in cells of the indicated genotypes after 2 days in culture. MMS treatment was 100 µM, 3 hrs. Mean ± S.D. of n=3 experiments shown. (**F**) DNA fiber analysis to measure fork stalling and restart after hydroxyurea treatment. (**G**) Measurement of asymmetry of replication from single replication origins, measured by DNA fiber analysis. The ratio of the longer replication tract compared to the shorter tract was quantified for each origin. Mean ± S.D. of n=3 experiments shown. (**H**) Measurement of replication fork velocity by DNA combing in untreated cells and after treatment with MMS. Mean ± S.D. of n=3 experiments shown. For parts **A**-**C** and **F**, error bars represent the S.D. of the means. P values were calculated by unpaired t-test (**A**-**C**, **F**), paired t-test (**E**, **G**), and one-way ANOVA with Tukey's multiple comparison test (**H**). P <0.05 was considered statistically significant.

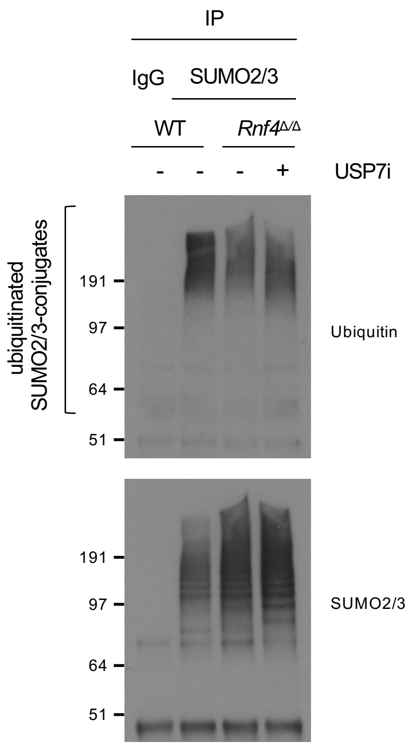

Supplemental Figure 3: Western blot to measure levels of ubiquitination of SUMO-conjugated proteins on chromatin in *Rnf4*$^{\Delta/\Delta}$ B cells after short-term treatment with USP7 inhibitor. B cells cultured in vitro for 48 hrs were either untreated (-) or treated with 50 μM P22077 for 4 hrs. SUMO2/3-conjugates were immunoprecipitated from the chromatin fraction of B cells using an anti-SUMO2/3 antibody, and subsequently used for detection of ubiquitin.

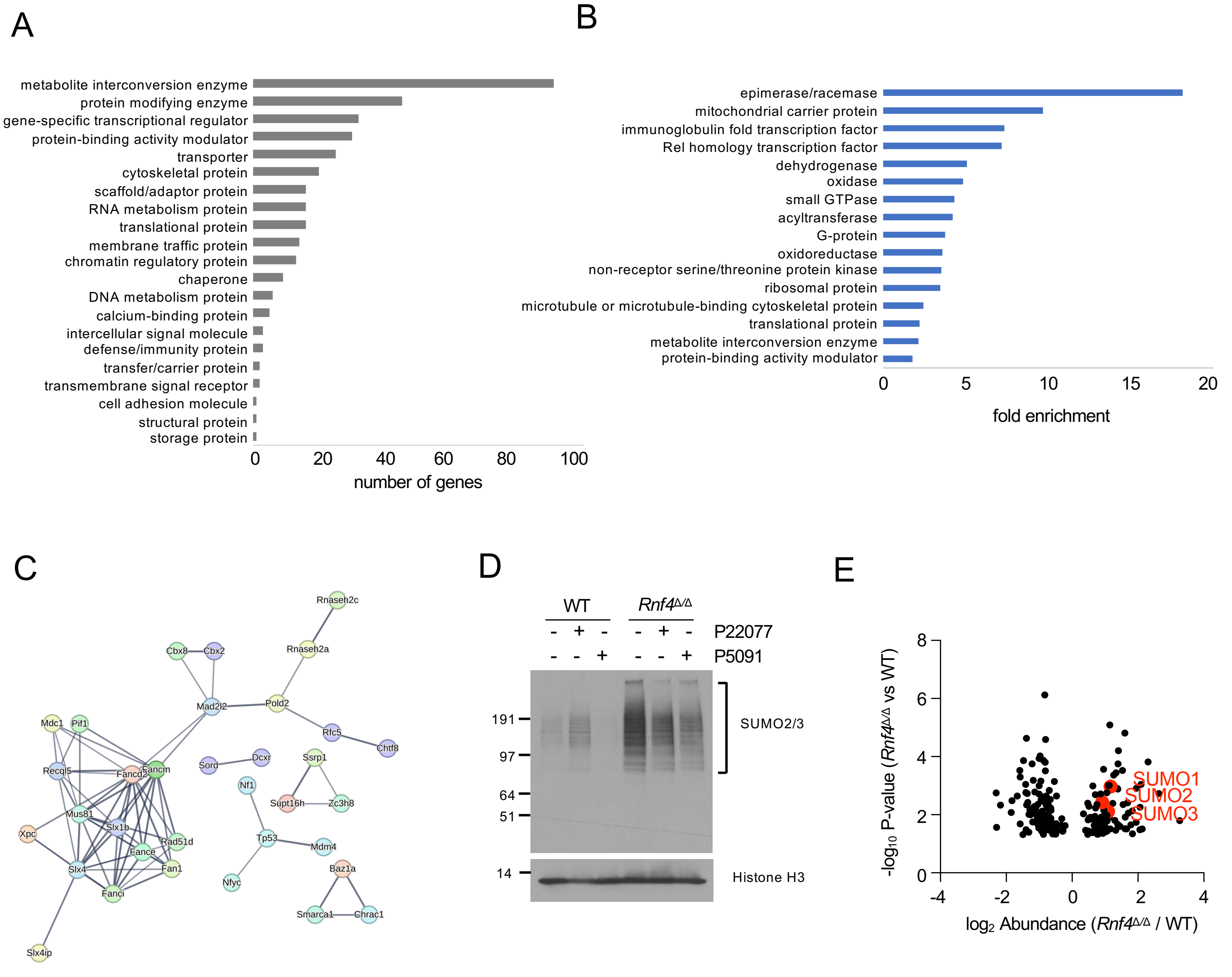

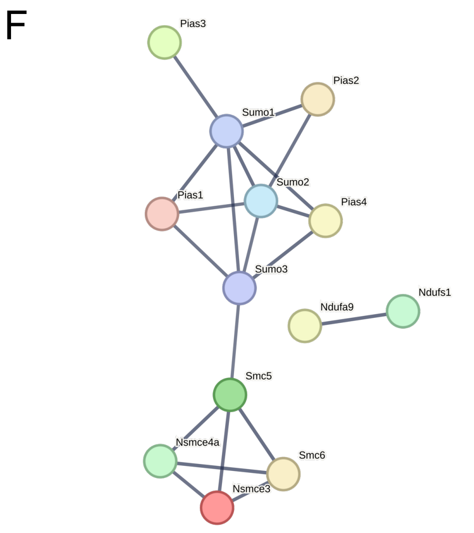

Supplemental Figure 4: (**A**) PANTHER protein class analysis of proteins showing increased abundance in *Rnf4*$^{\Delta/\Delta}$ B cells relative to WT after 48 hrs in vitro culture. (**B**) PANTHER over-representation test showing enrichment of protein classes among 455 proteins with increased abundance in *Rnf4*$^{\Delta/\Delta}$ B cells cultured in vitro for 48 hrs. (**C**) STRING functional enrichment analysis of proteins showing significant changes in abundance at replication forks, as measured by iPOND using B cells from WT and *Rnf4*$^{\Delta/\Delta}$ B cells. (**D**) Western blot of chromatin extracts from cells grown in vitro for two days ± continual treatment with USP7 inhibitors, P22077 (2 μM) or P5091 (1 μM). (**E**) Volcano plot showing protein abundance in chromatin samples from WT and *Rnf4*$^{\Delta/\Delta}$ B cells after immunoprecipitation of SUMO2/3. Each data point represents the average of four samples. (**F**) STRING functional enrichment analysis of proteins showing significant changes in abundance in chromatin samples from WT and *Rnf4*$^{\Delta/\Delta}$ B cells after immunoprecipitation of SUMO2/3.

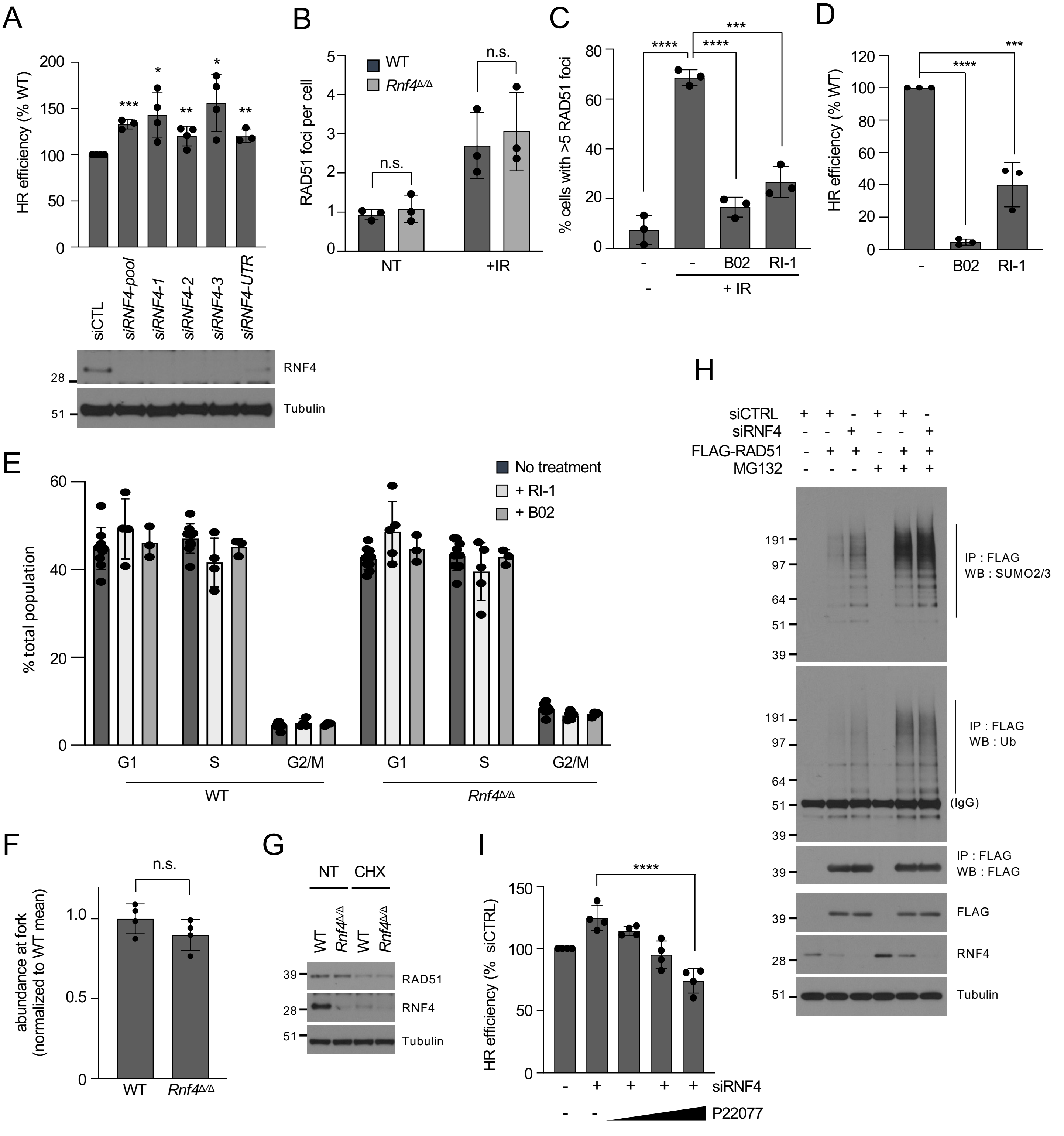

Supplemental Figure 5: (**A**) Measurement of homologous recombination frequency by DR-GFP assay after transfection with either control siRNA (siCTRL) or siRNA oligonucleotides targeting *RNF4*. Efficiency of knockdown is shown by Western blot in the lower panel. (**B**) Quantification of RAD51 foci in cells after no treatment (NT), or after 4 hrs recovery from treatment with 10 Gy of ionizing radiation (IR). (**C**) Analysis of proportion of WT cells showing >5 ionizing radiation-induced nuclear foci after either no treatment (-) or with the RAD51 inhibitors, B02 and RI-1 (1 hr, 50 μM). (**D**) Homologous recombination efficiency measured by DR-GFP assay in cells that were either untreated (-) or treated for 1 hr with B02 (20 μM) or RI-1 (50 μM). (**E**) Measurement of cell cycle by quantification of EdU incorporation and DAPI binding in splenic B cells after 48 hrs growth in vitro ± 10 μM RI-1 or 5 μM B02. (**F**) Abundance of RAD51 at active replication forks, measured by iPOND-MS in n=4 replicate samples. (**G**) RAD51 abundance measured by Western blot in cells that were either not treated (NT) or treated with 25 μg/ml cycloheximide (CHX) for 4 hrs. (**H**) Analysis of ubiquitin present in FLAG-RAD51 pulldown samples in cells treated with either control siRNA (siCTRL) or siRNA targeting RNF4 (siRNF4). Some samples were treated with the proteasome inhibitor, MG132 (10 μM, 4 hrs). (**I**) Homologous recombination efficiency measured by DR-GFP in cells that were treated ± siRNF4 and /or the USP7 inhibitor, P22077 (2, 5, 10μM). Error bars in **A**-**F**, and **I** represent the S.D. of the mean. P values were calculated by unpaired t-test (**A**, **B**, **F**), one-way ANOVA with Tukey's multiple comparison test (**C**, **I**), and one-way ANOVA with Dunnett's multiple comparison test (**D**). P < 0.05 was considered significant.

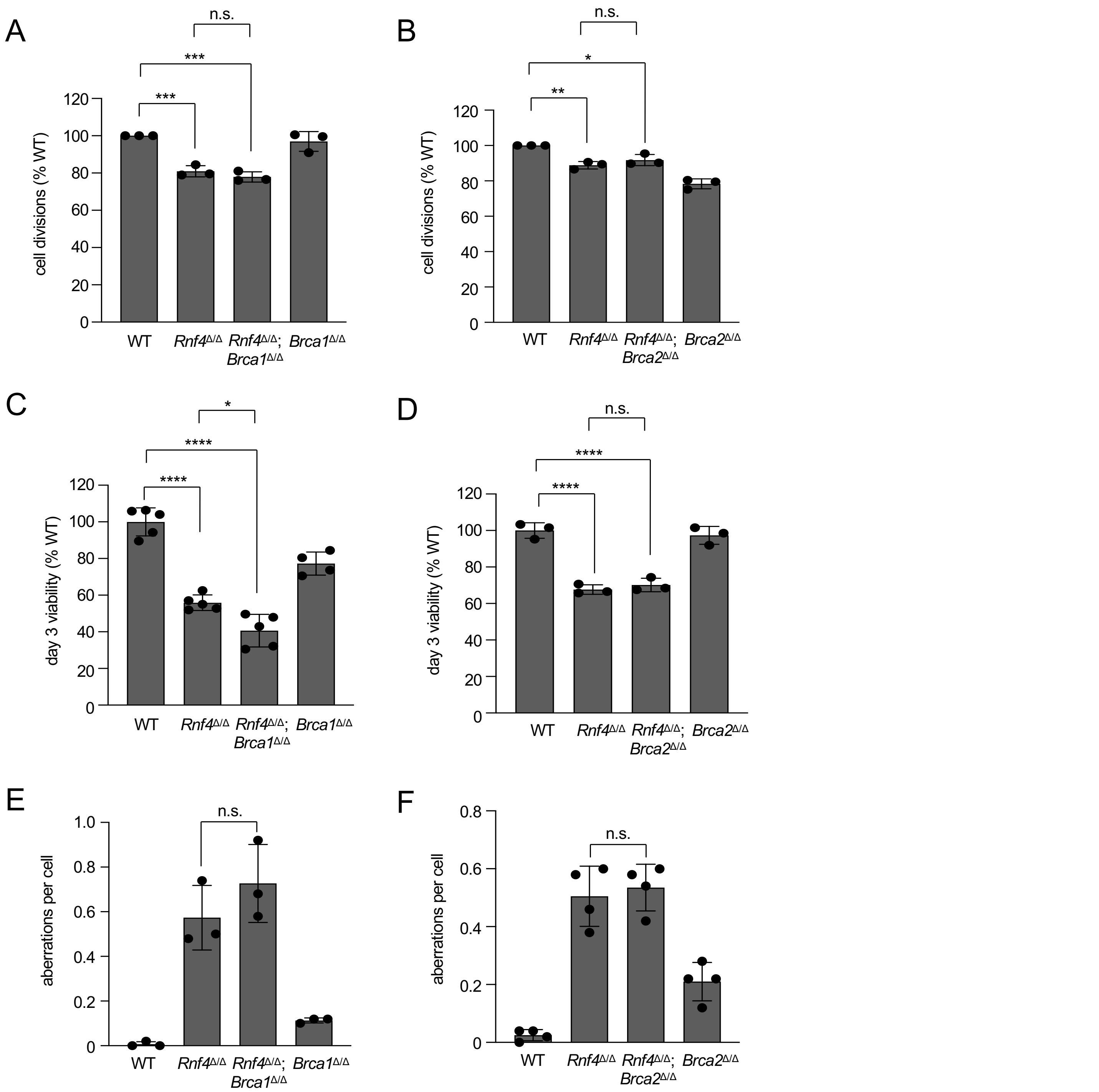

Supplemental Figure 6: (**A**) Quantification of proliferation of B cells growing in vitro for 72 hrs, measured by flow cytometry analysis of CFSE dilution. *Rnf4* and *Brca1* were deleted either singly or in combination in the B cell lineage by breeding to a CD19-Cre transgenic line. (**B**) as (**A**) but with conditional deletion of *Brca2* on a WT or $Rnf4^{\Delta/\Delta}$ background. (**C**) Quantification of cell viability of cells of the indicated genotypes, after three days of in vitro B cell culture. Cell viability was measured by DAPI exclusion, as detected by flow cytometry. (**D**) As (**C**) but with deletion of *Brca2*. (**E**) Quantification of chromosome aberrations of B cells of the indicated genotypes. (**F**) As (**E**) but with conditional deletion of *Brca2.* Error bars show the S.D. of the mean in each case, with P values calculated by one-way ANOVA with Tukey's multiple comparison test. A P value < 0.05 was considered statistically significant.

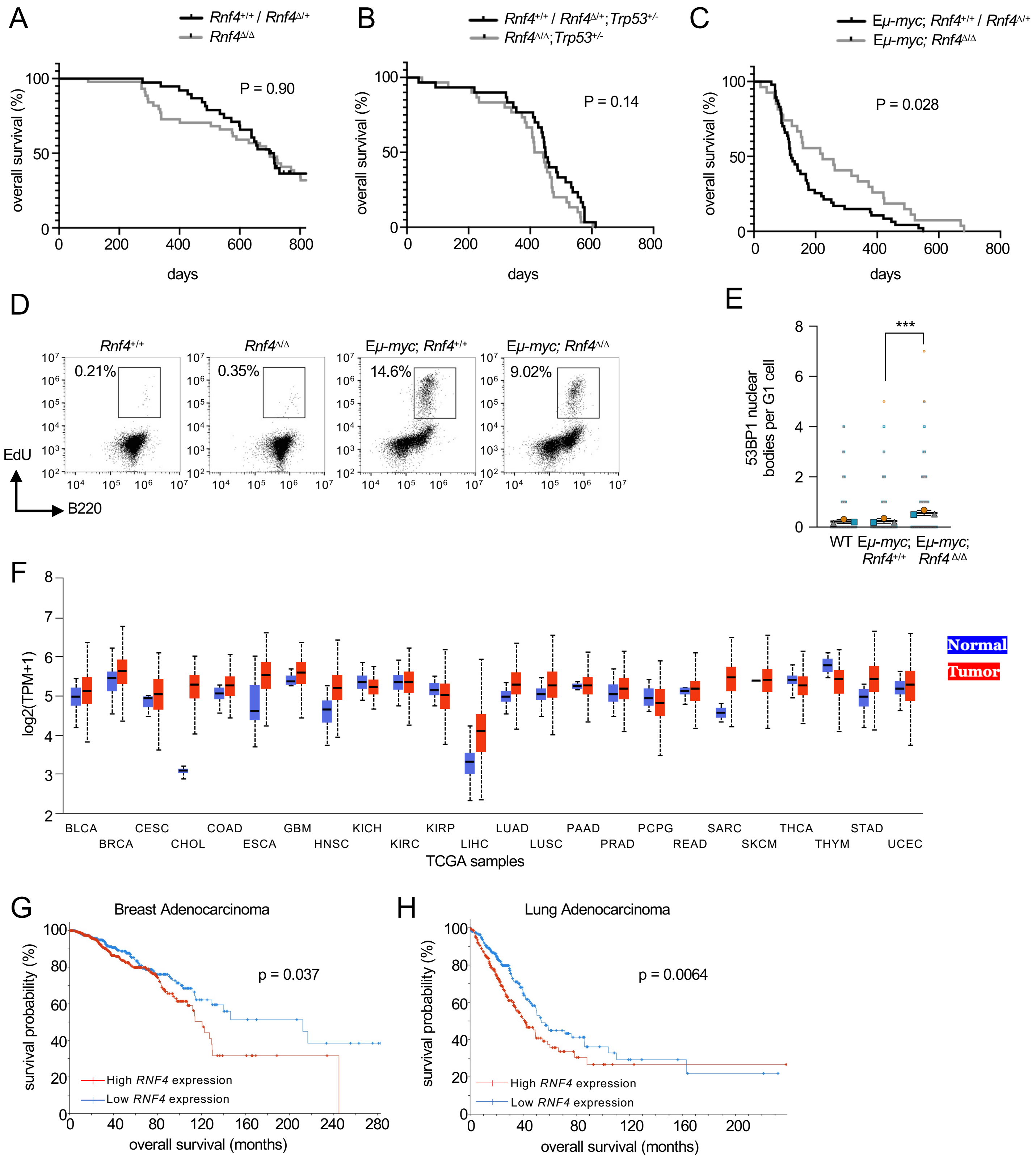

Supplemental Figure 7: (**A**) Kaplan-Meier analysis showing overall survival of *Rnf4*$^{+/+}$;*CD19-Cre* or *Rnf4*$^{f/+}$;*CD19-Cre* (n = 38), and *Rnf4*$^{f/f}$;*CD19-Cre* (n = 44). (**B**) Kaplan-Meier analysis of overall survival of *Rnf4*$^{+/+}$;*Trp53*$^{+/-}$;*CD19-Cre* or *Rnf4*$^{f/+}$;*Trp53*$^{+/-}$;*CD19-Cre* (n = 30), and *Rnf4*$^{f/f}$;*Trp53*$^{+/-}$;*CD19-Cre* (n = 30). (**C**) Kaplan-Meier analysis of overall survival of E*μ-myc*;*Rnf4*$^{+/+}$;*CD19-Cre* or E*μ-myc*;*Rnf4*$^{f/+}$;*CD19-Cre* (n = 47), and E*μ-myc*;*Rnf4*$^{f/f}$;*CD19-Cre* (n = 27). (**D**) Flow cytometry analysis showing EdU uptake by B220^{+} resting B cells from the spleens of WT, *Rnf4*$^{\Delta/\Delta}$, and E*μ-myc* transgenic mice. (**E**) Quantification of 53BP1 G1 nuclear bodies, measured by anti-53BP1 immunofluorescence in G1 cells of the indicated genotypes after two days of in vitro culture. Mean ± S.D. of n=3 experiments shown. (**F**) Expression of *RNF4* in different tumor types according to TCGA data sets. (**G**) Survival of breast adenocarcinoma patients with tumors expressing either above-median (n = 541) or below-median (n = 541) levels of RNF4. Data from TCGA PanCancer Atlas retrieved through cBioPortal. (**H**) Survival of patients with lung adenocarcinoma expressing either above-median (n = 249) or below-median (n = 252) levels of RNF4. P values in **A**-**C** and **G**-**H** calculated by log-rank test. P value in **E** calculated by paired t-test. P < 0.05 was considered significant in each case.
