# Supplementary material for: RNF4 sustains Myc-driven tumorigenesis by facilitating DNA replication
Source: J Clin Invest. 2024 Mar 26;134(10):e167419. doi: 10.1172/JCI167419 (PMC11093604; doi:10.1172/JCI167419)

Full unedited bolt for Figure 1C

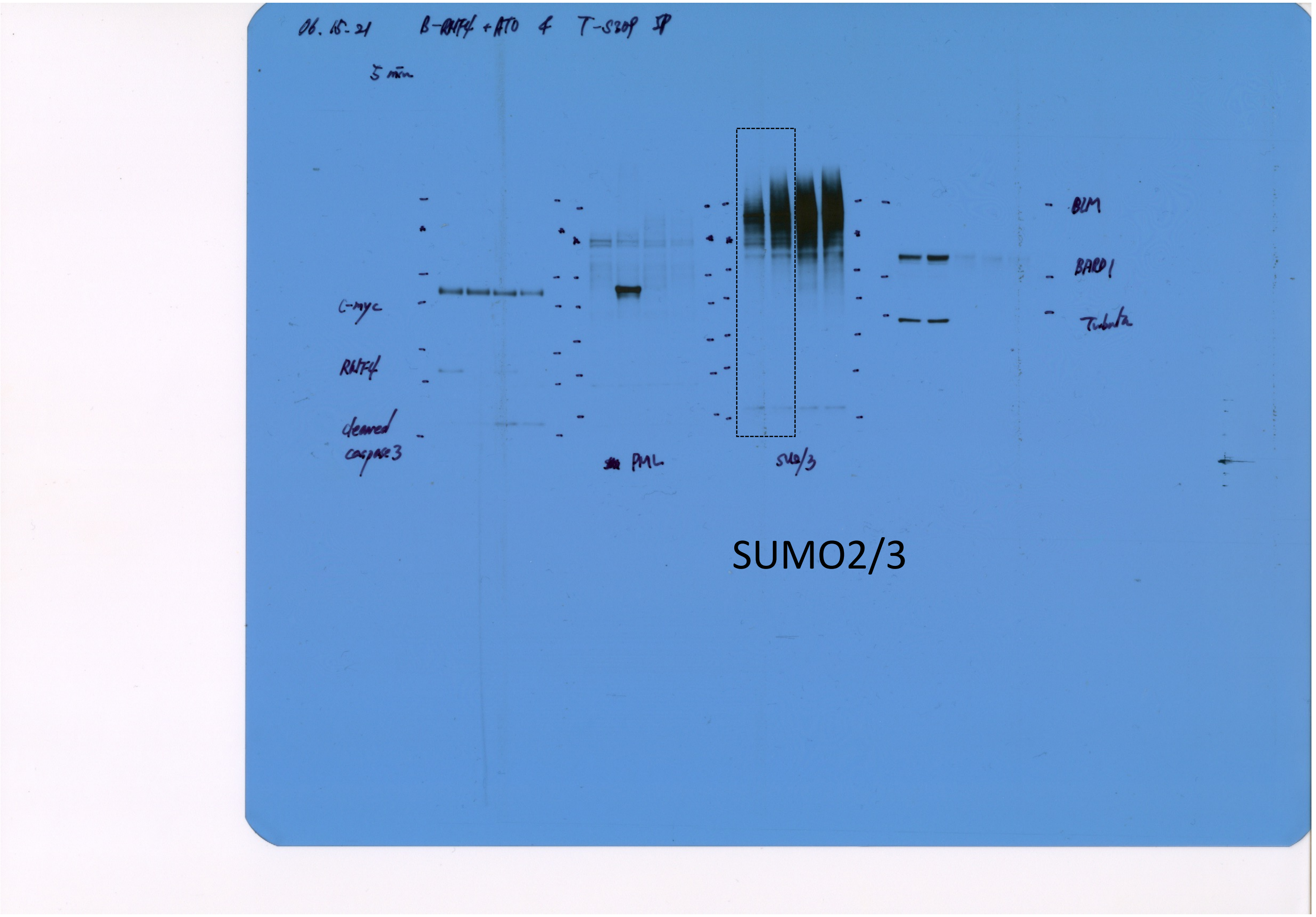

Full unedited bolt for Figure 1C

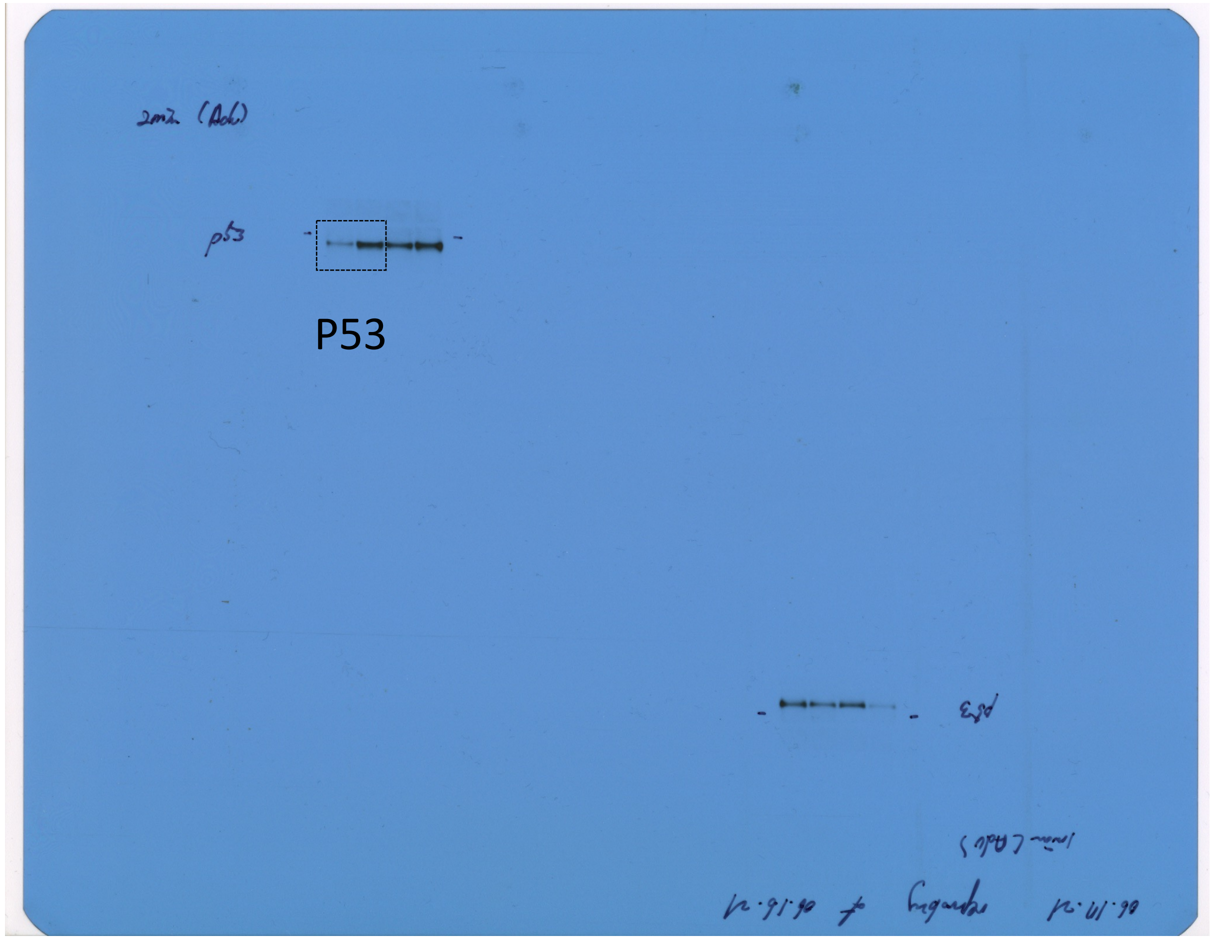

Full unedited bolt for Figure 1C

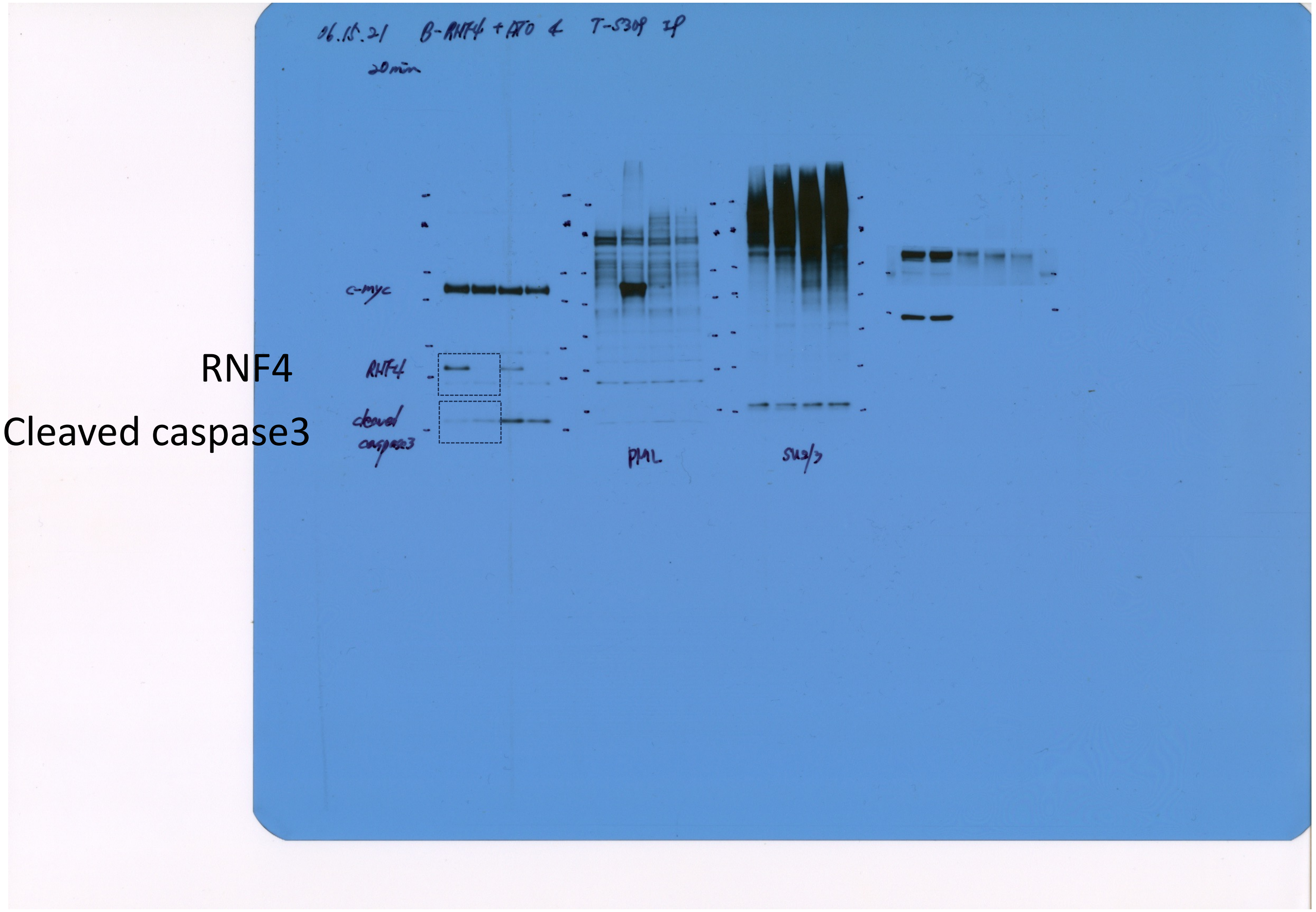

Full unedited bolt for Figure 1C

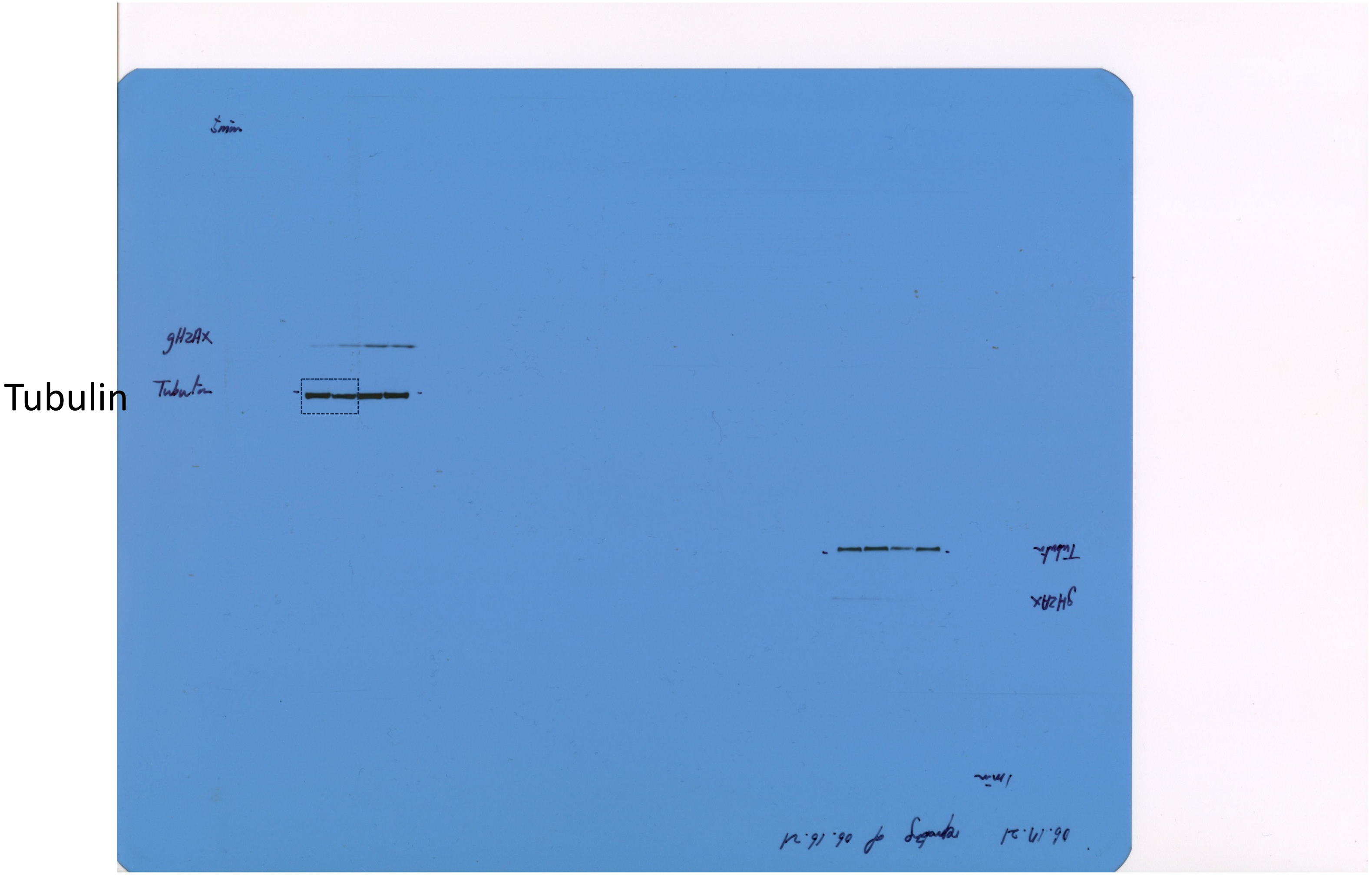

Full unedited bolt for Figure 1D

KAP1-p824

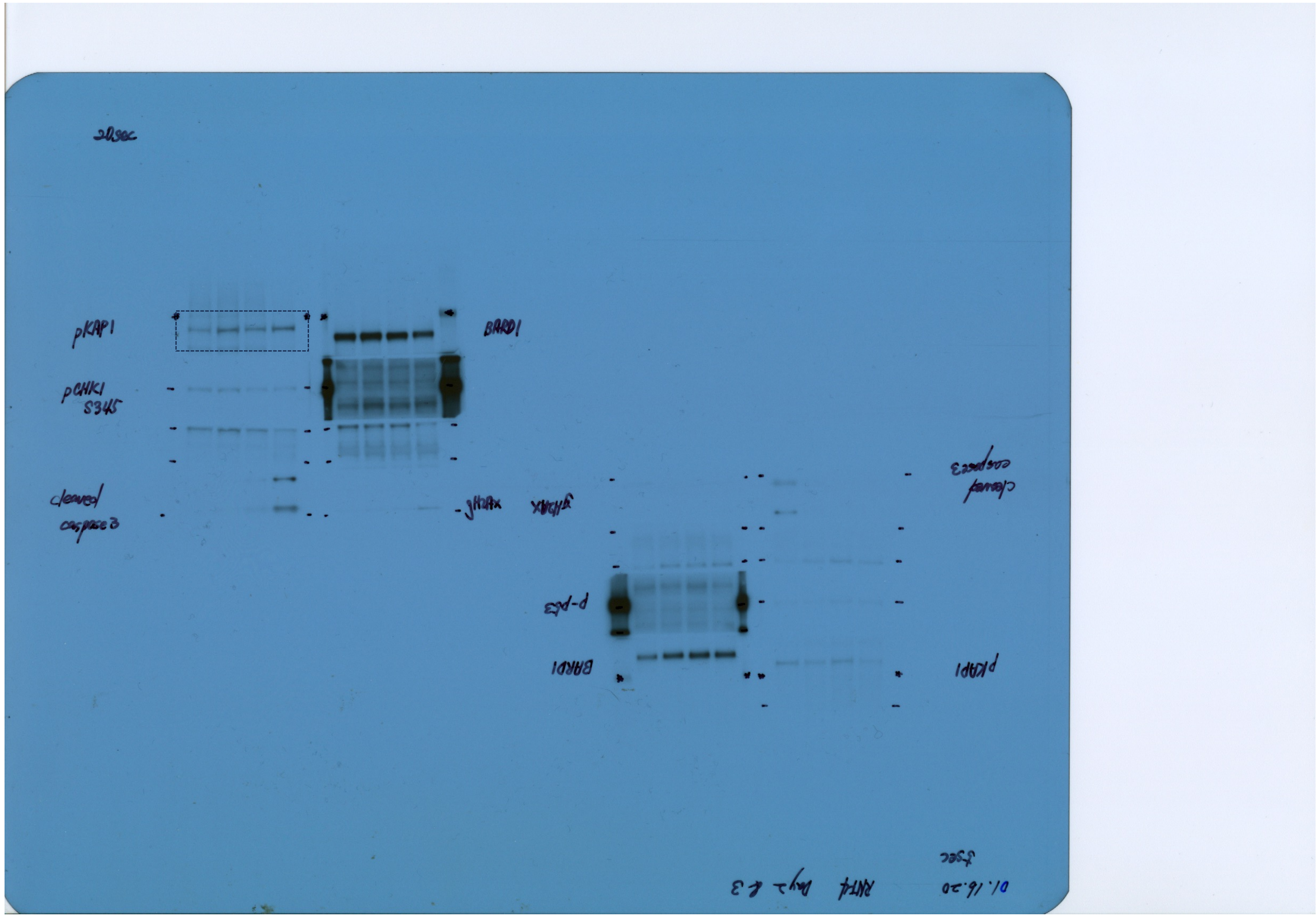

Full unedited bolt for Figure 1D

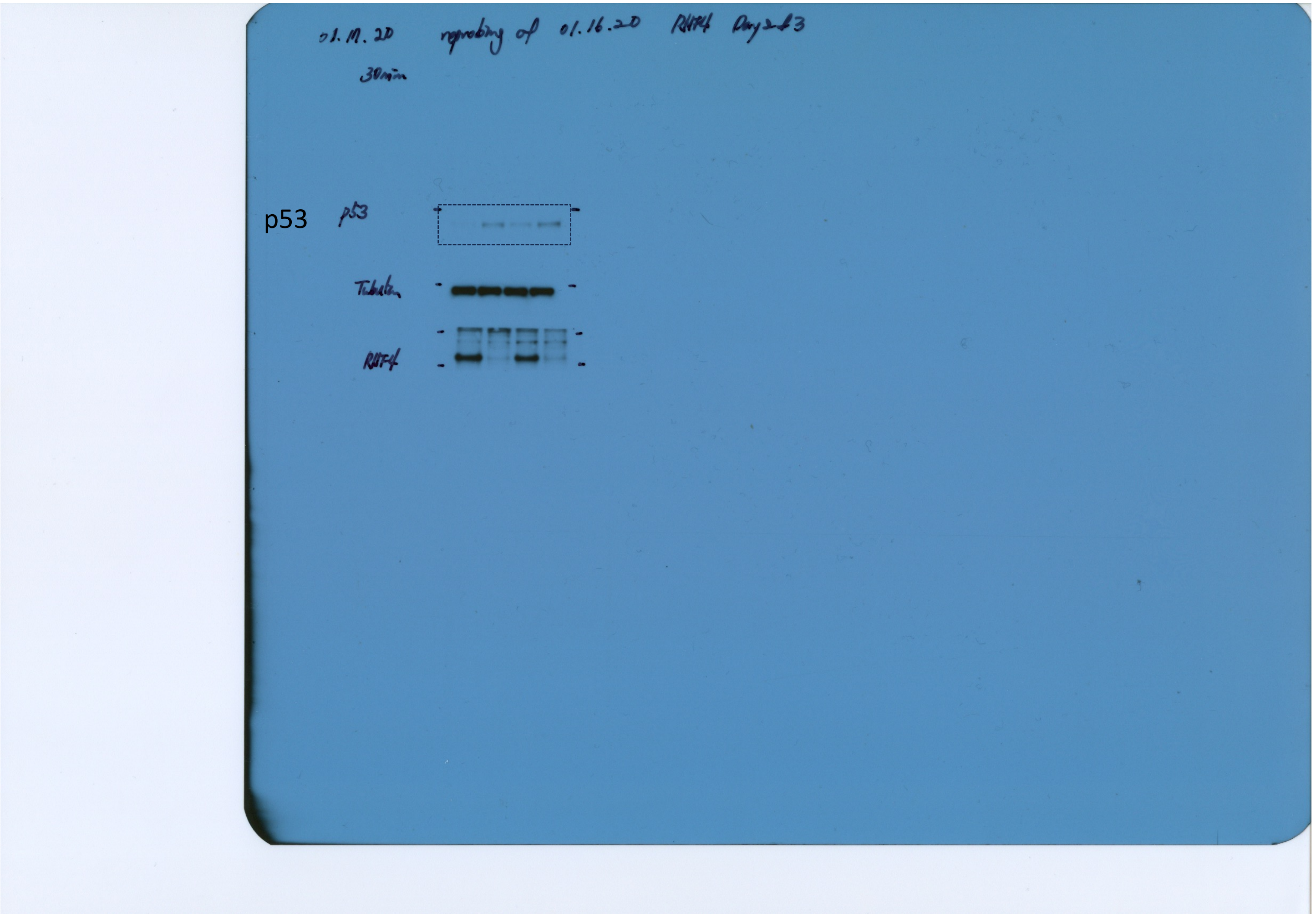

Full unedited bolt for Figure 1D

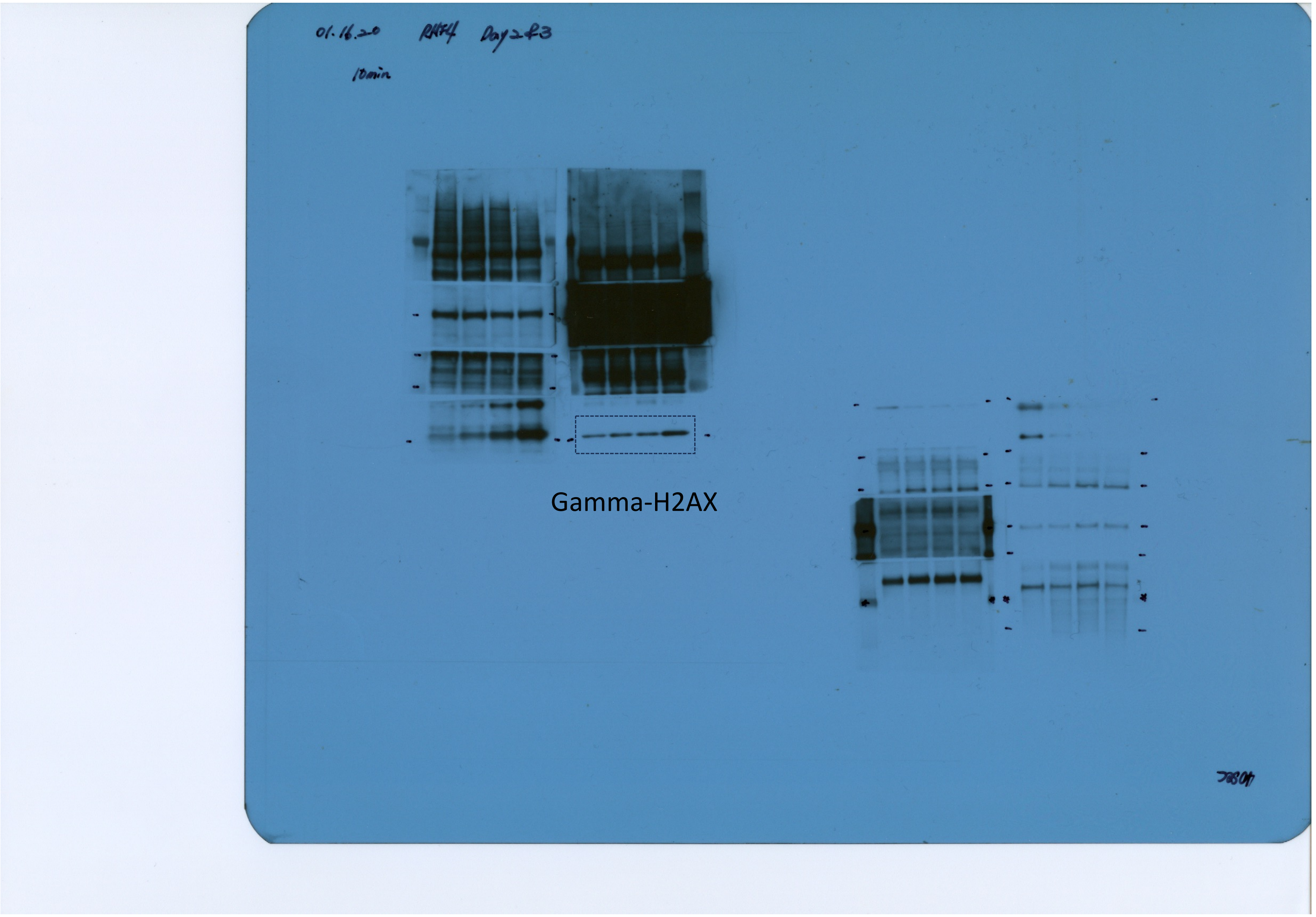

Full unedited bolt for Figure 1D

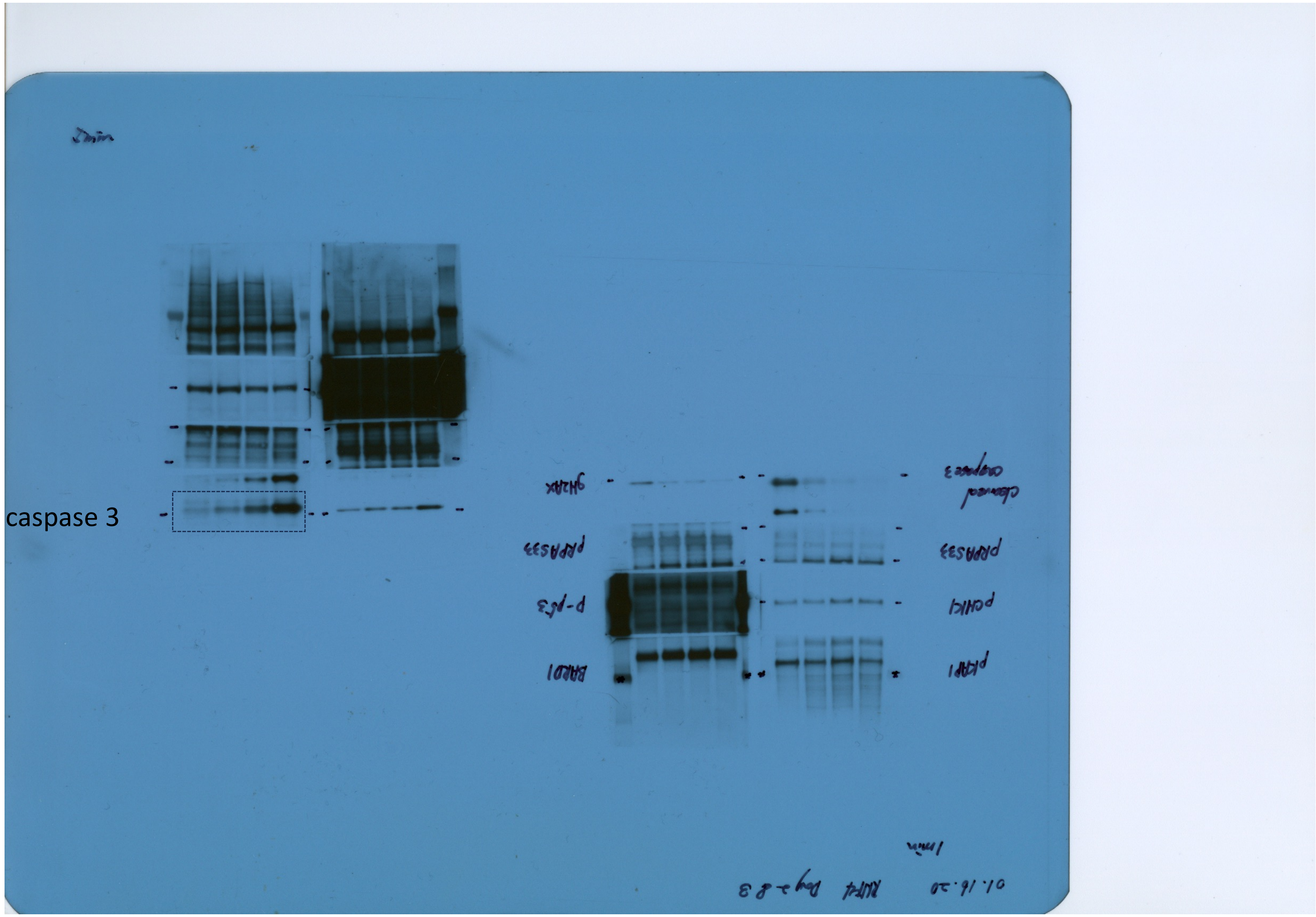

Cleaved caspase 3

Full unedited bolt for Figure 1D

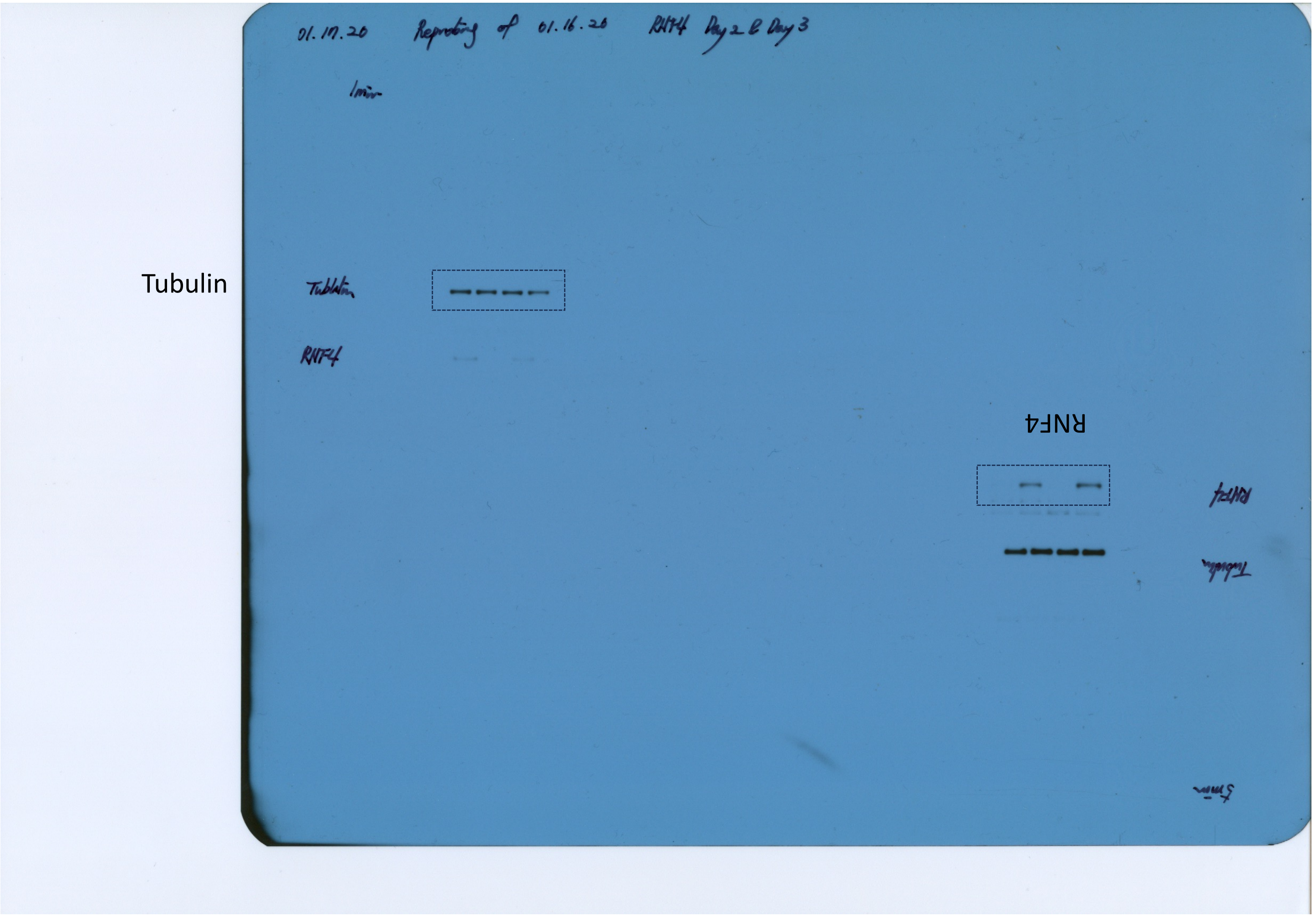

Full unedited bolt for Figure 2C

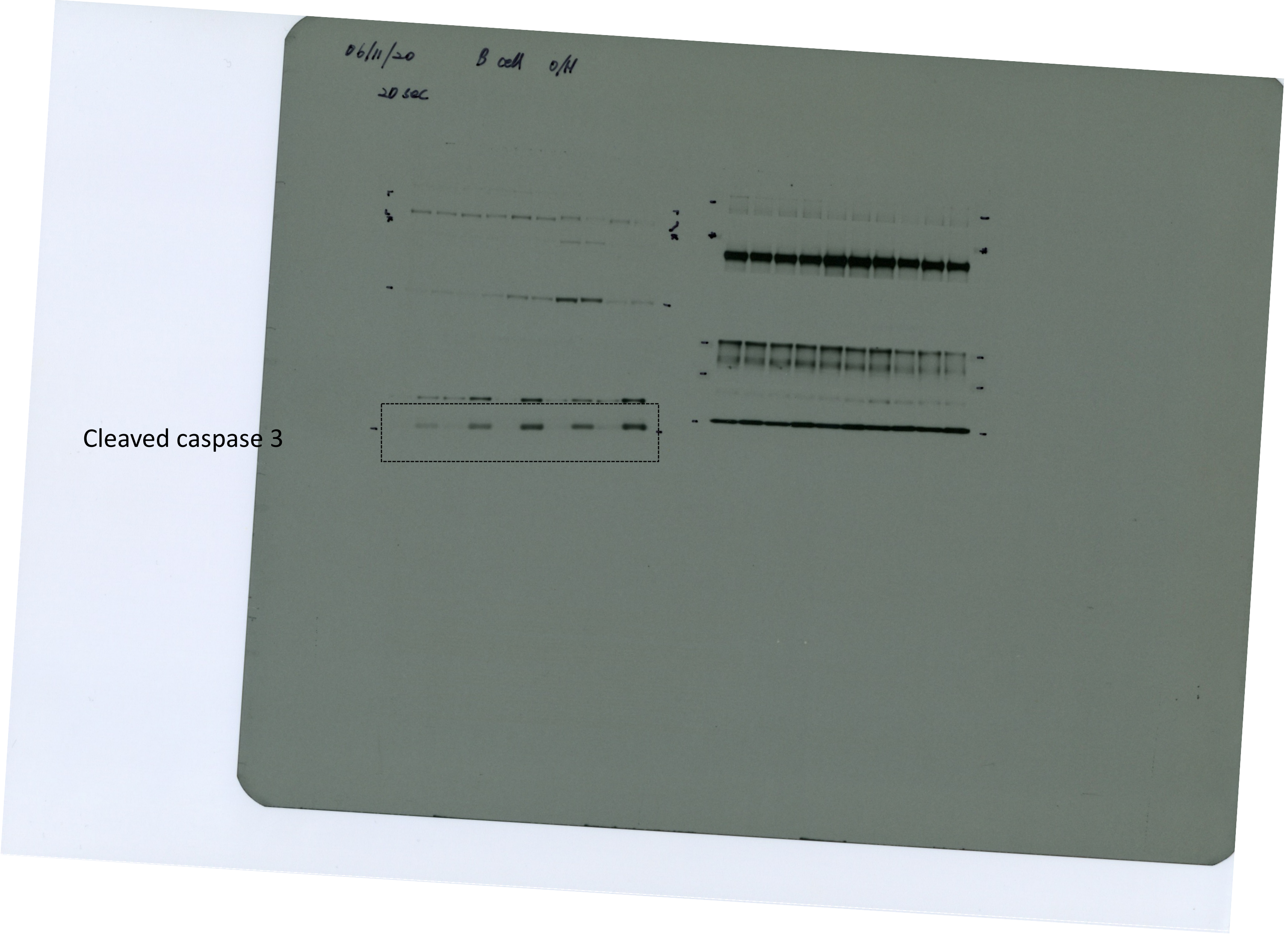

Full unedited bolt for Figure 2C

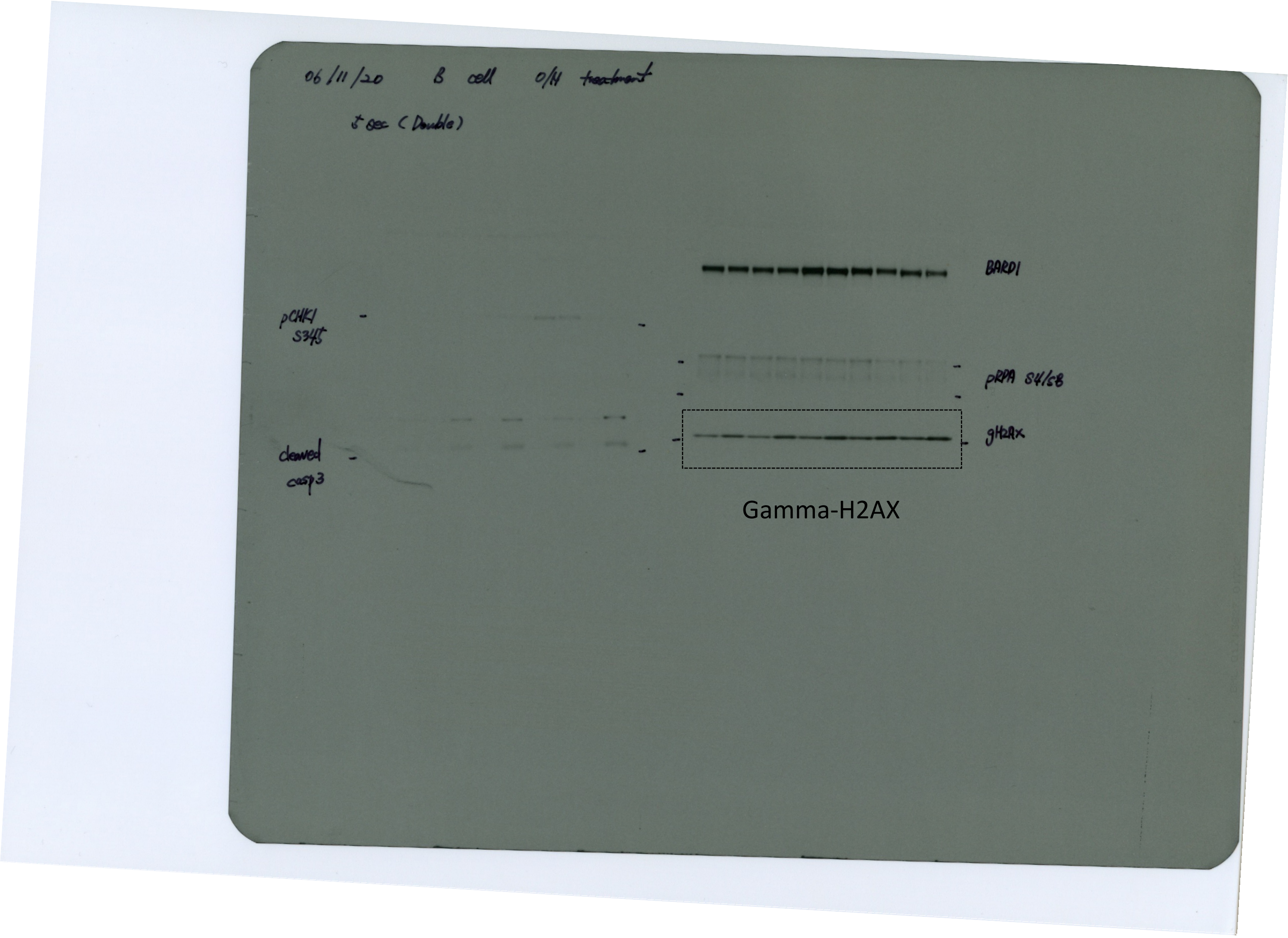

Full unedited bolt for Figure 2C

RNF4

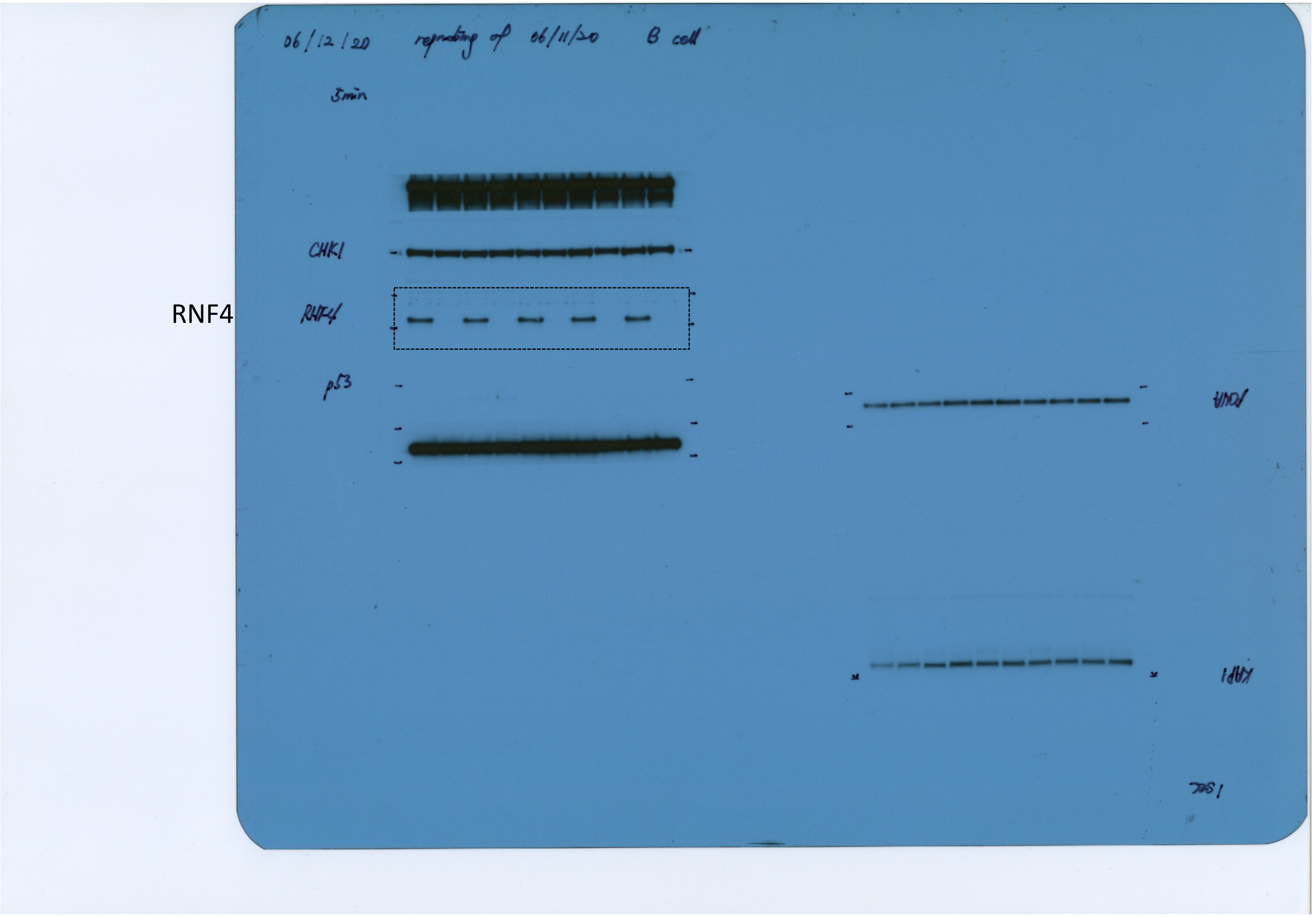

Full unedited bolt for Figure 2C

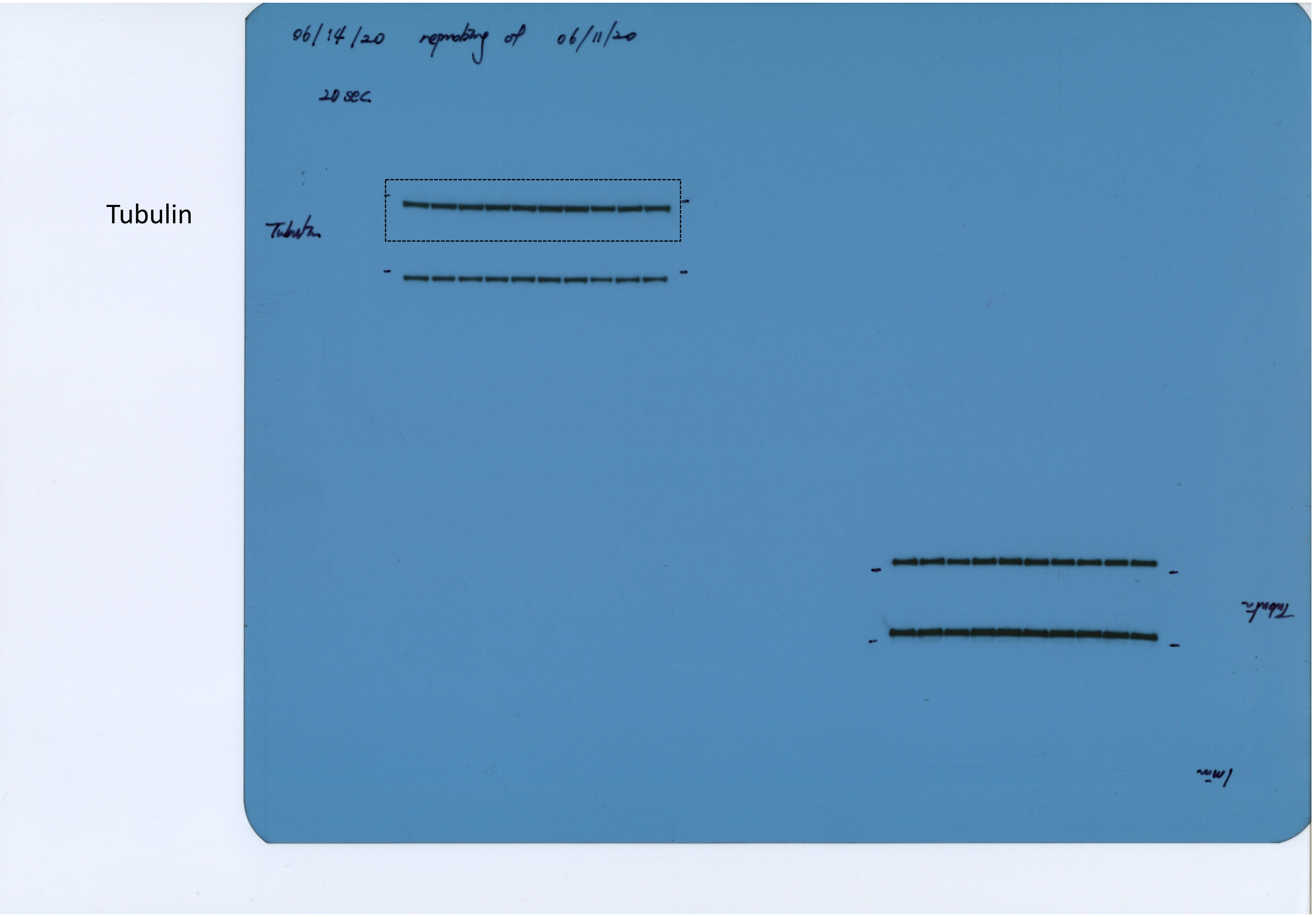

Full unedited bolt for Figure 2I

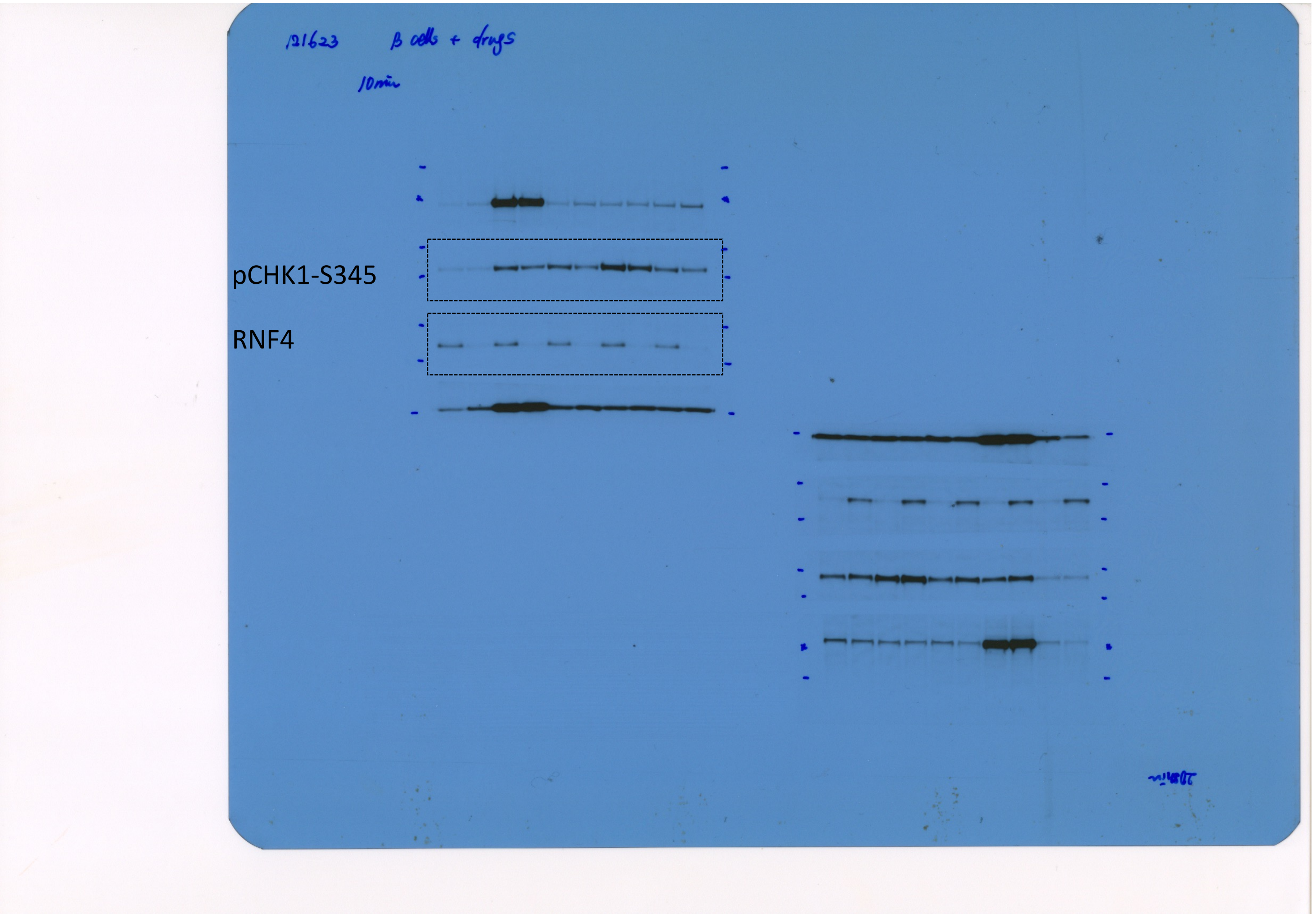

Full unedited bolt for Figure 2I

Gamma-H2AX

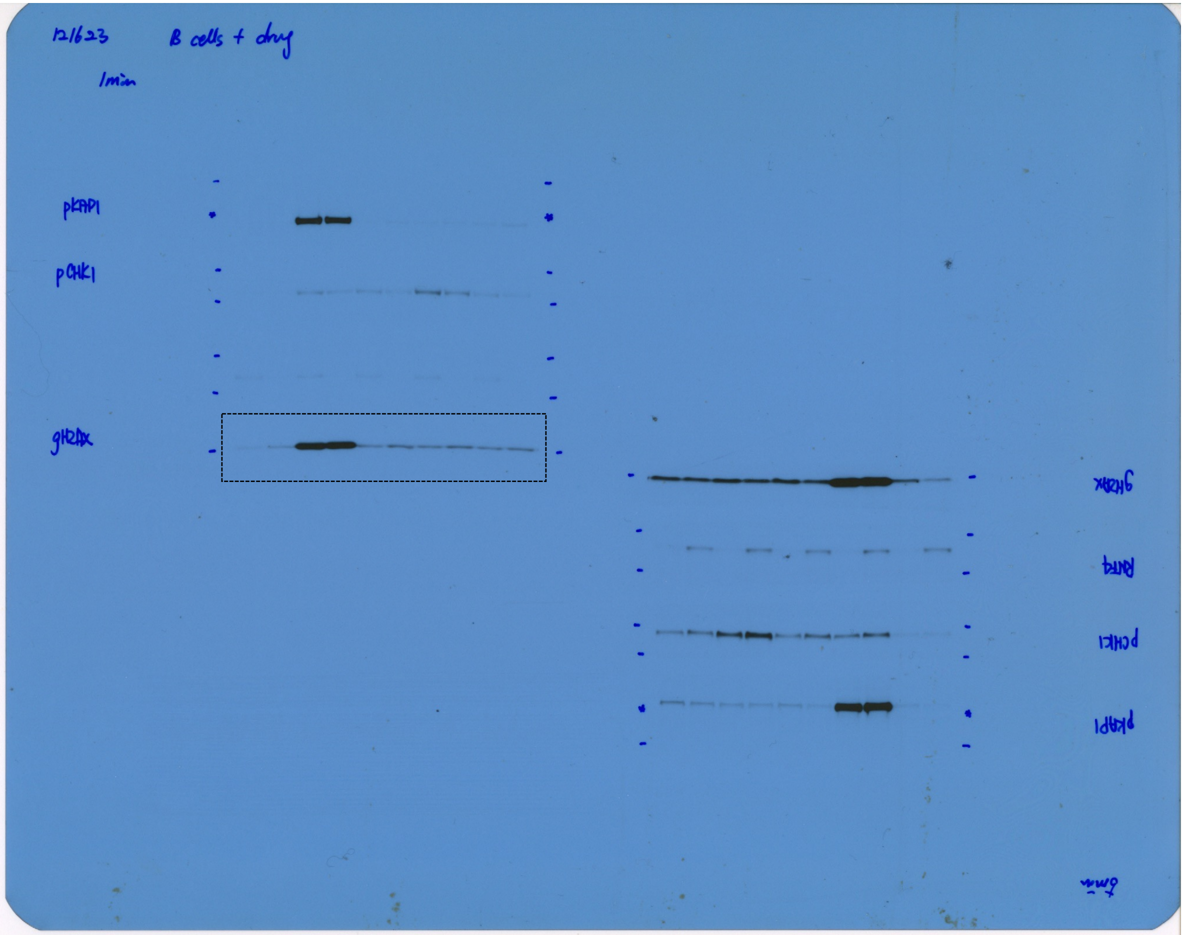

Full unedited bolt for Figure 2I

CHK1

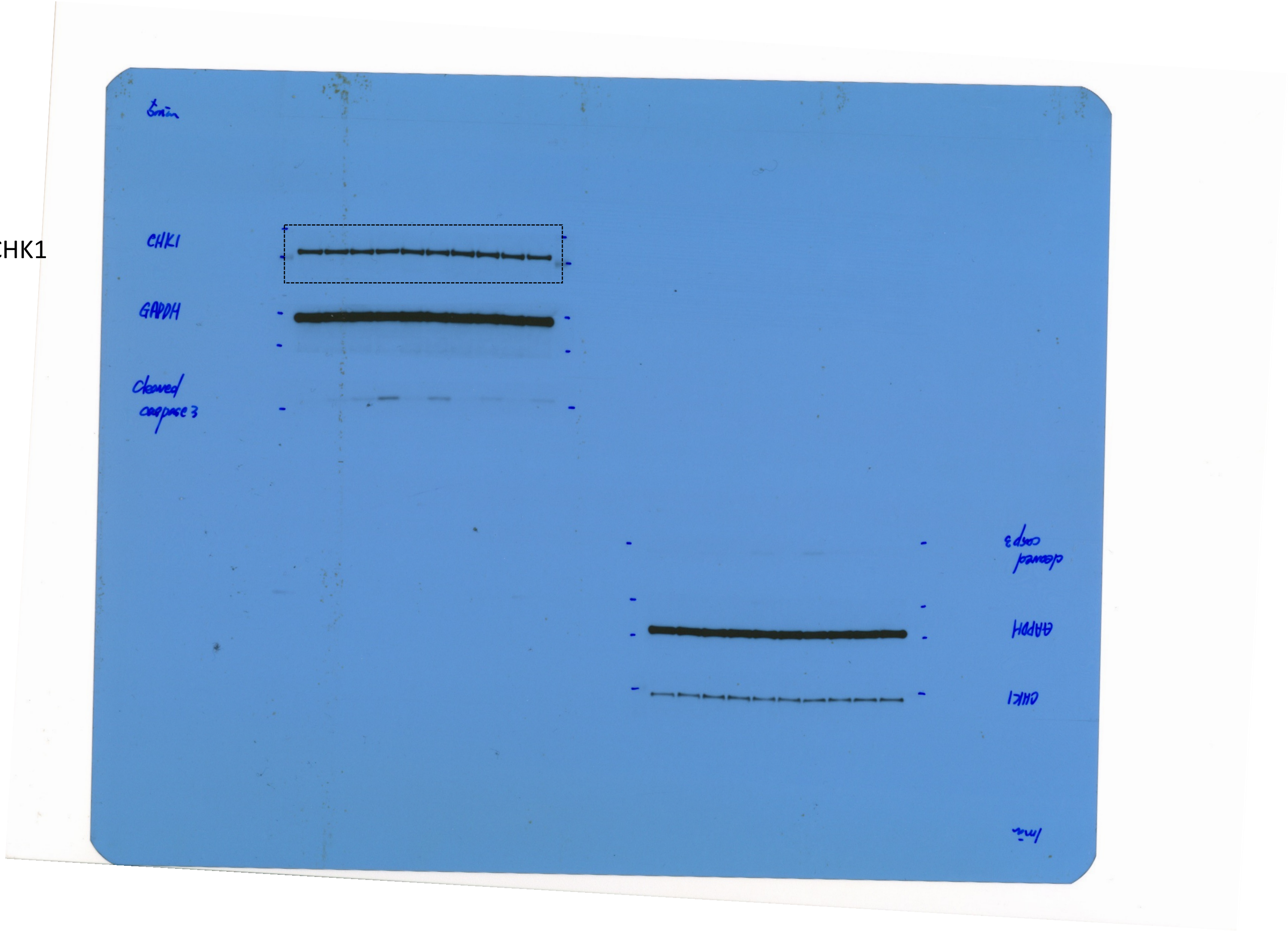

Full unedited bolt for Figure 2I

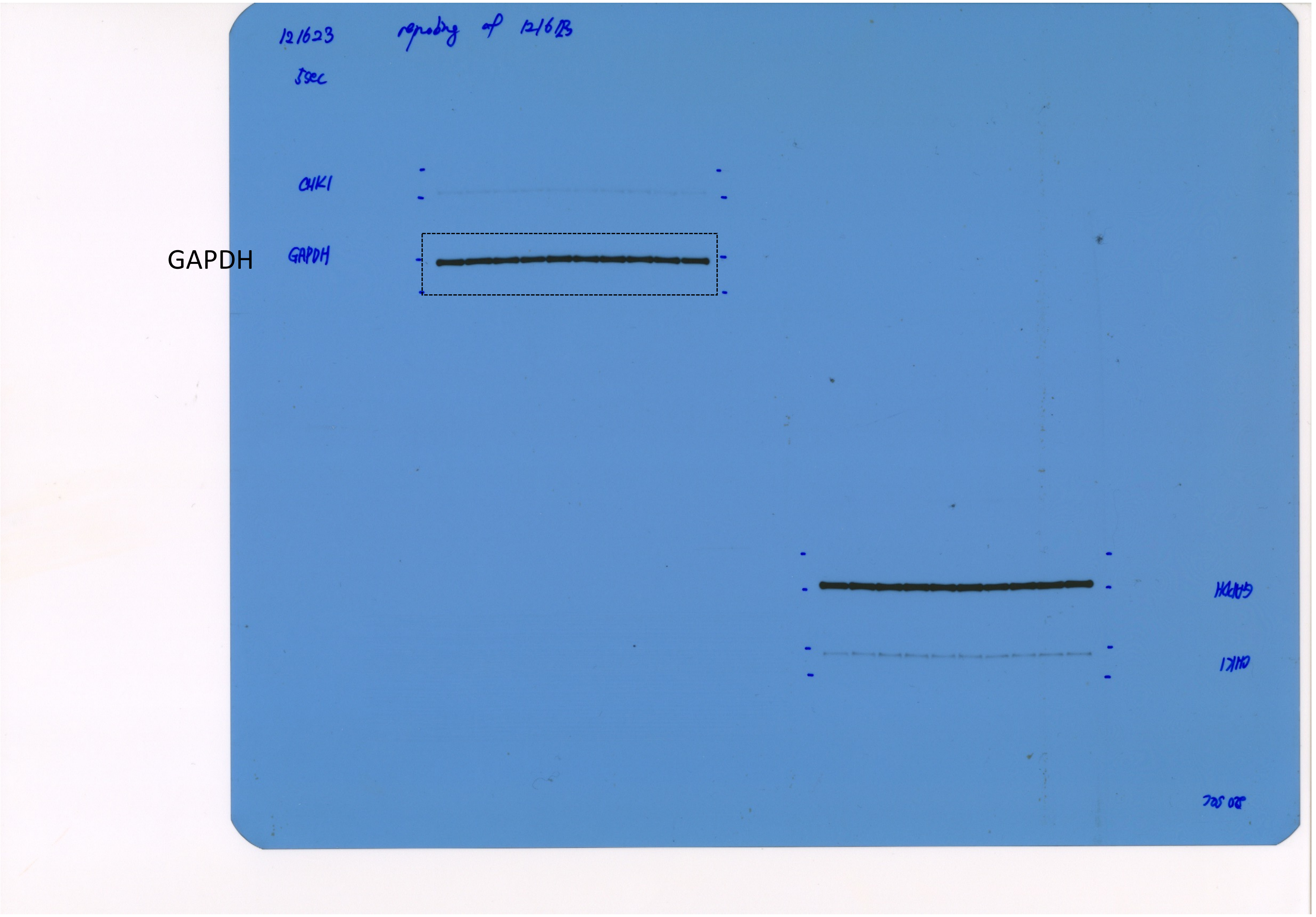

Full unedited bolt for Figure 3C

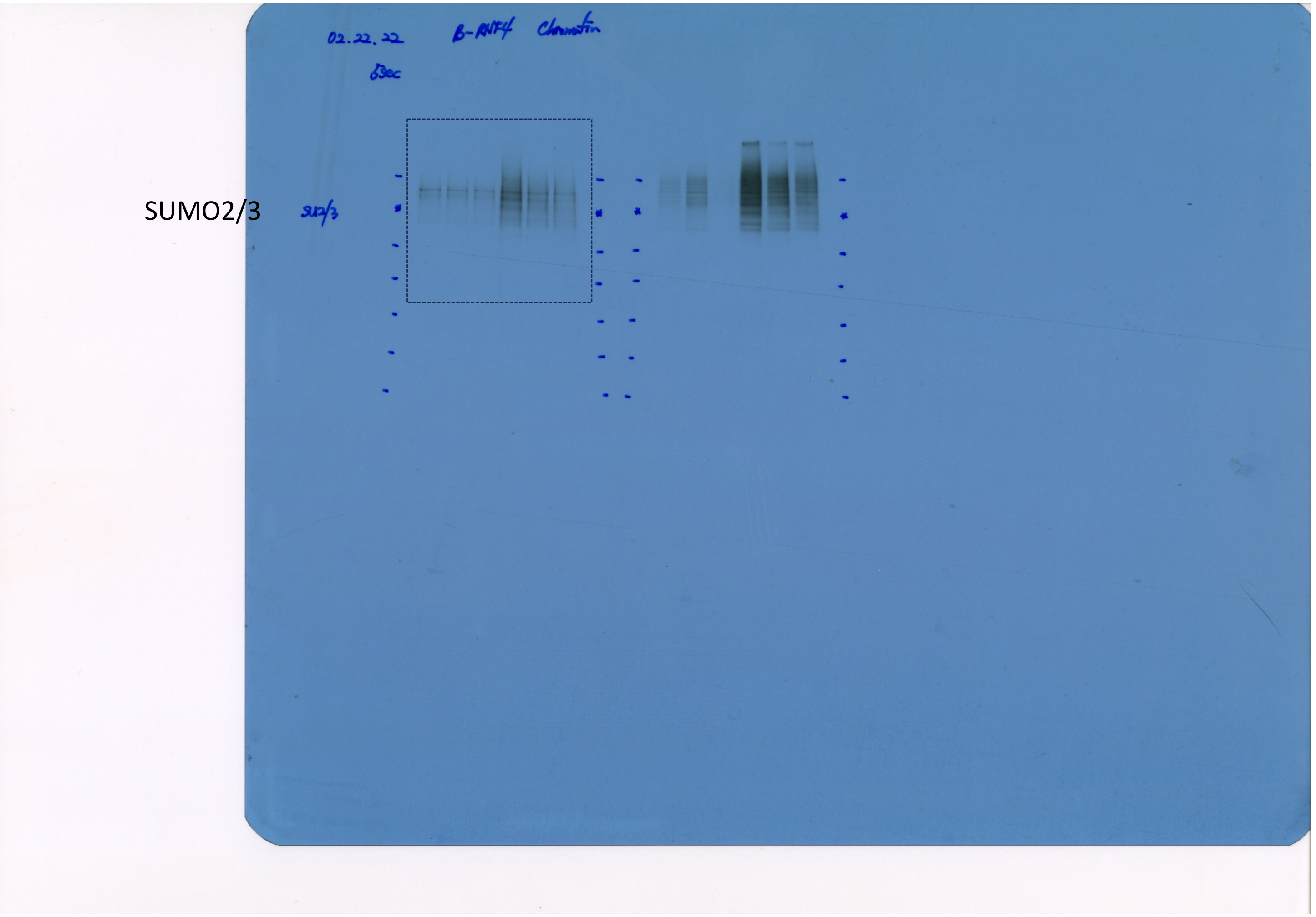

Full unedited bolt for Figure 3C

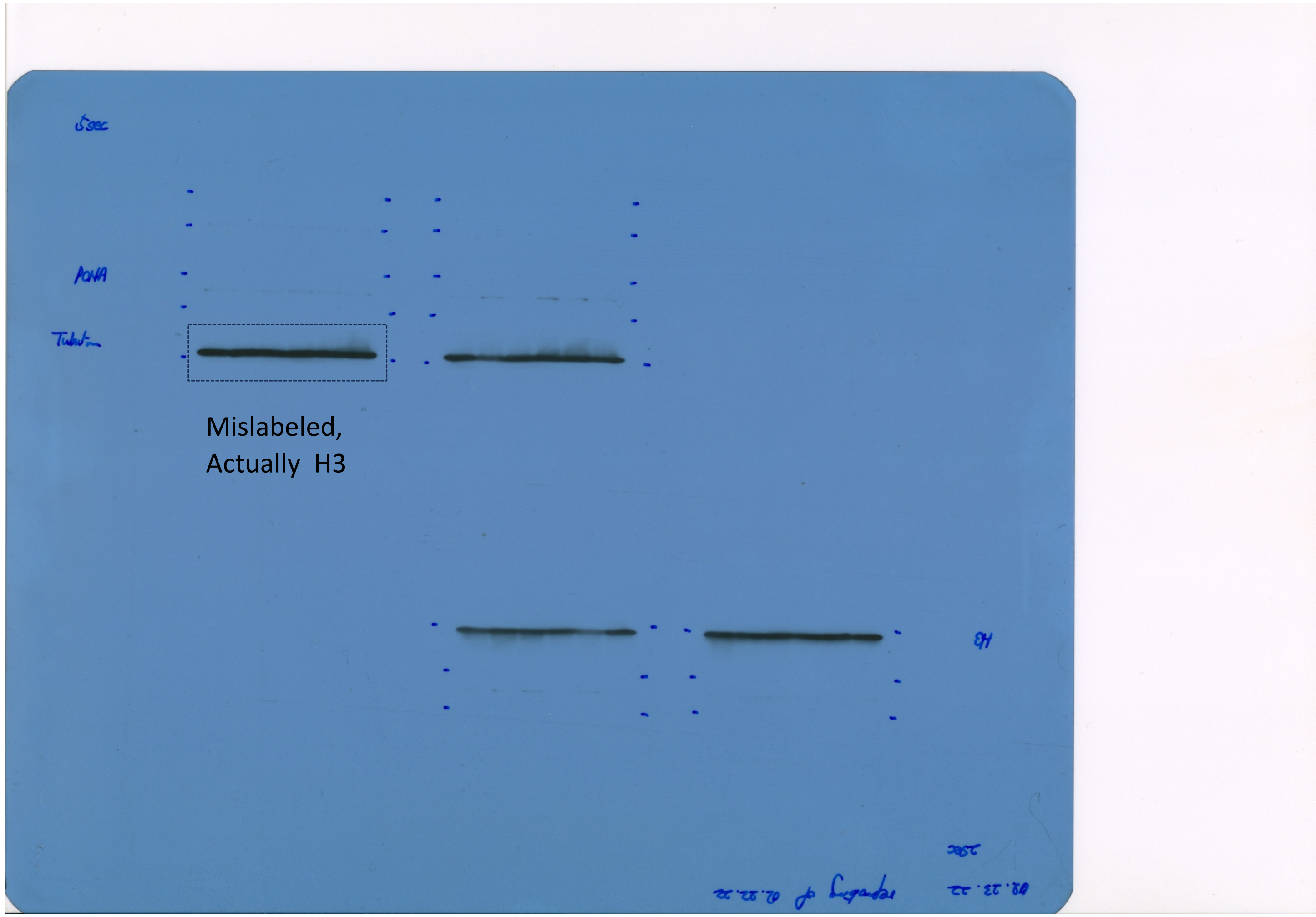

Full unedited bolt for Figure 4C

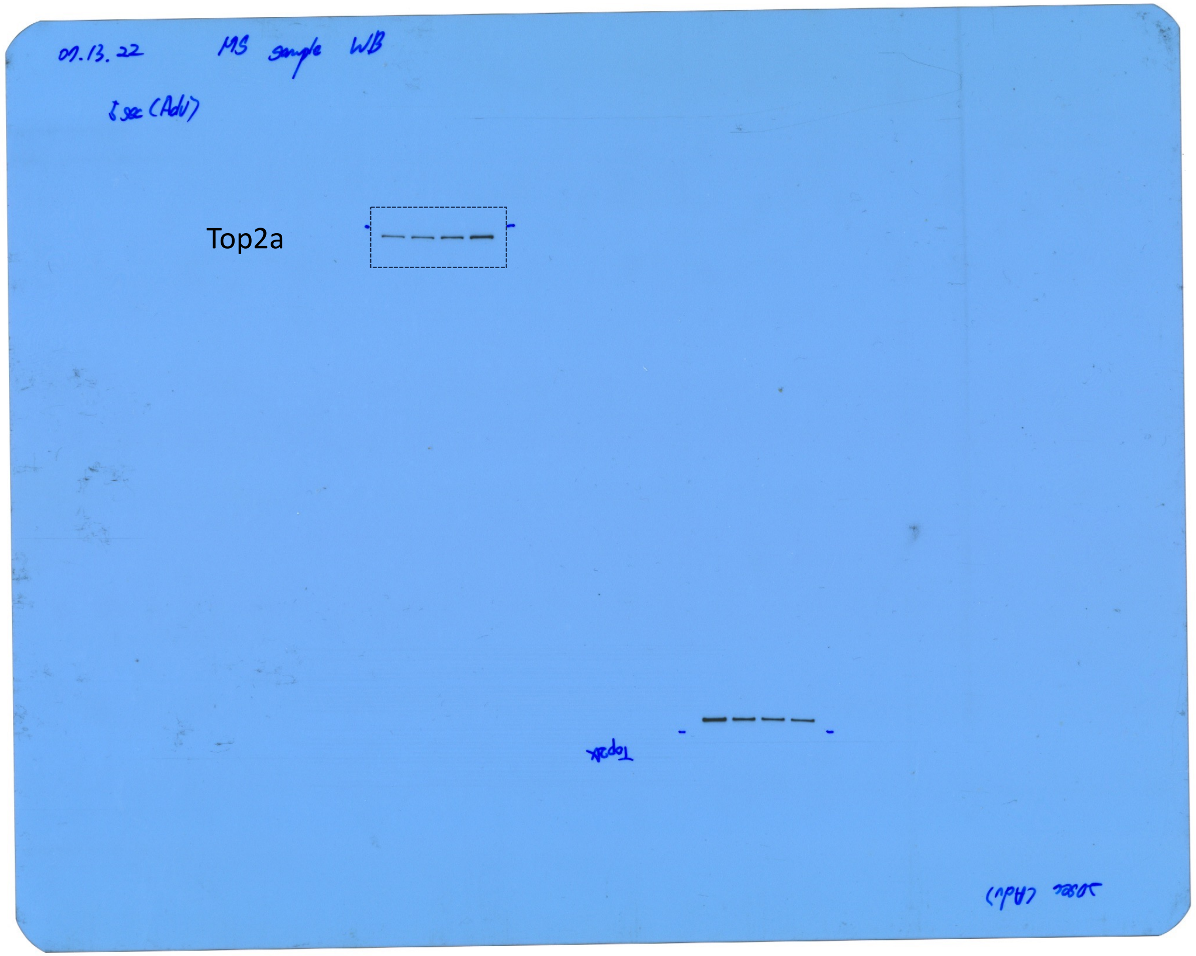

Full unedited bolt for Figure 4C

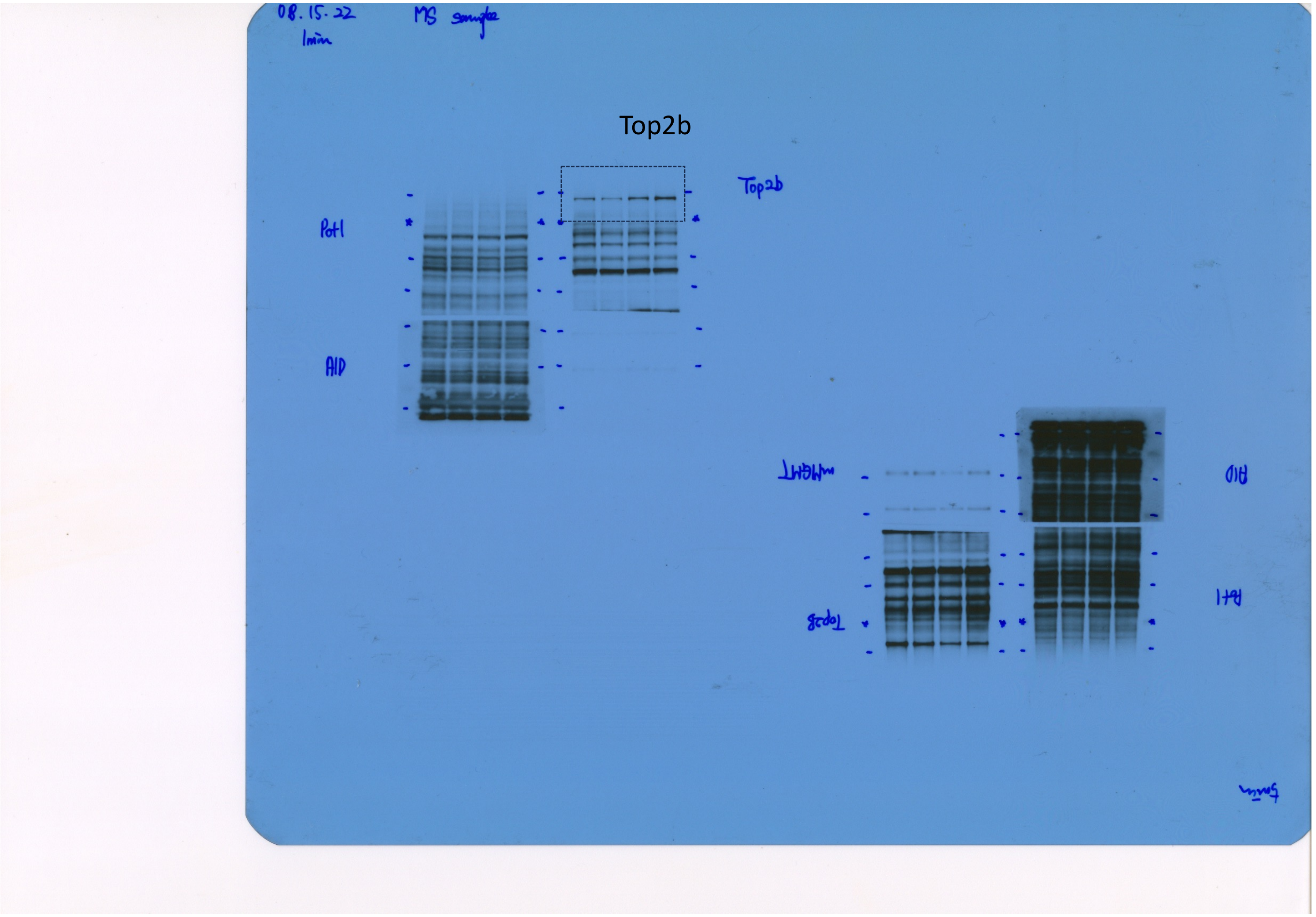

Full unedited bolt for Figure 4C

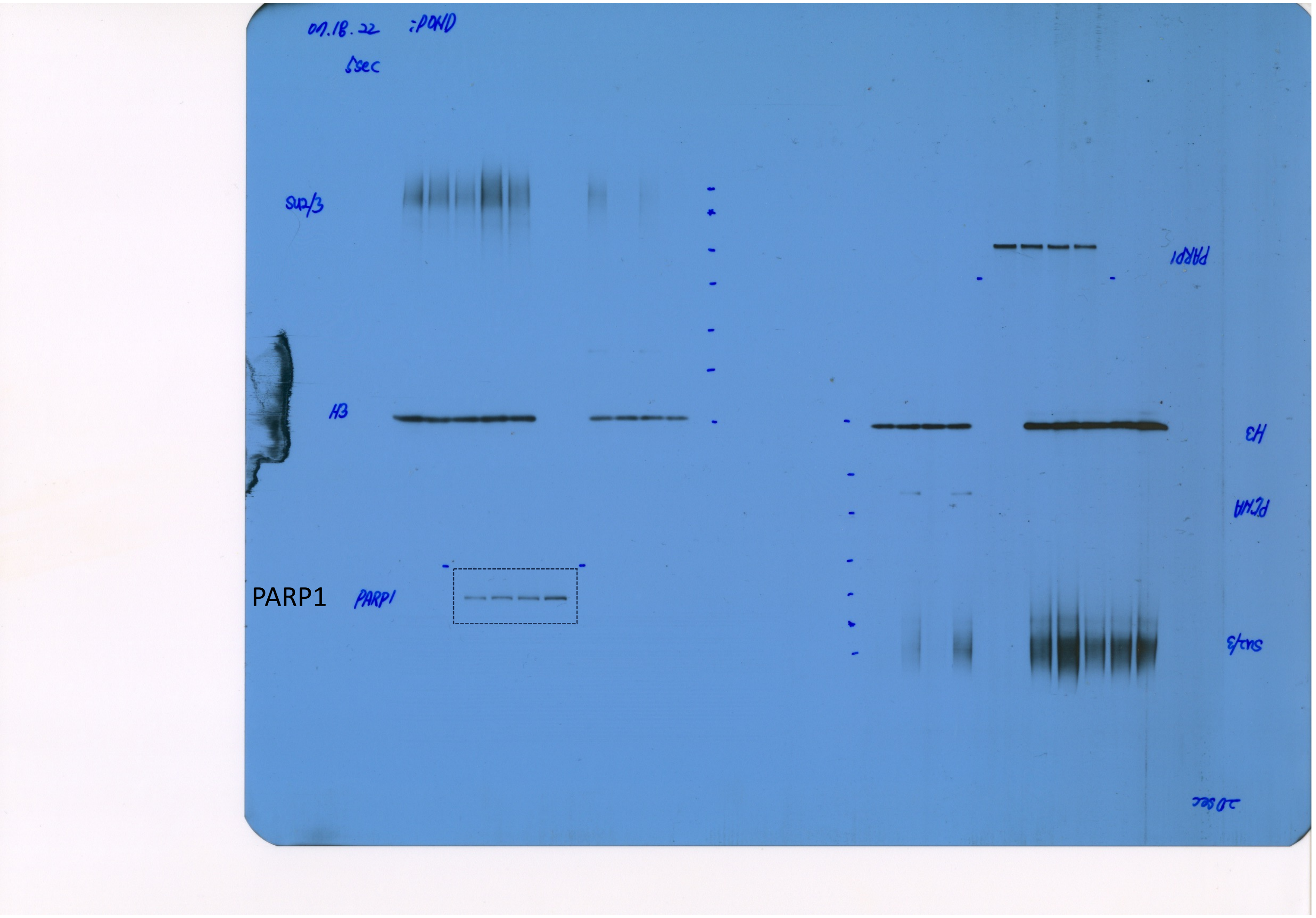

Full unedited bolt for Figure 4C

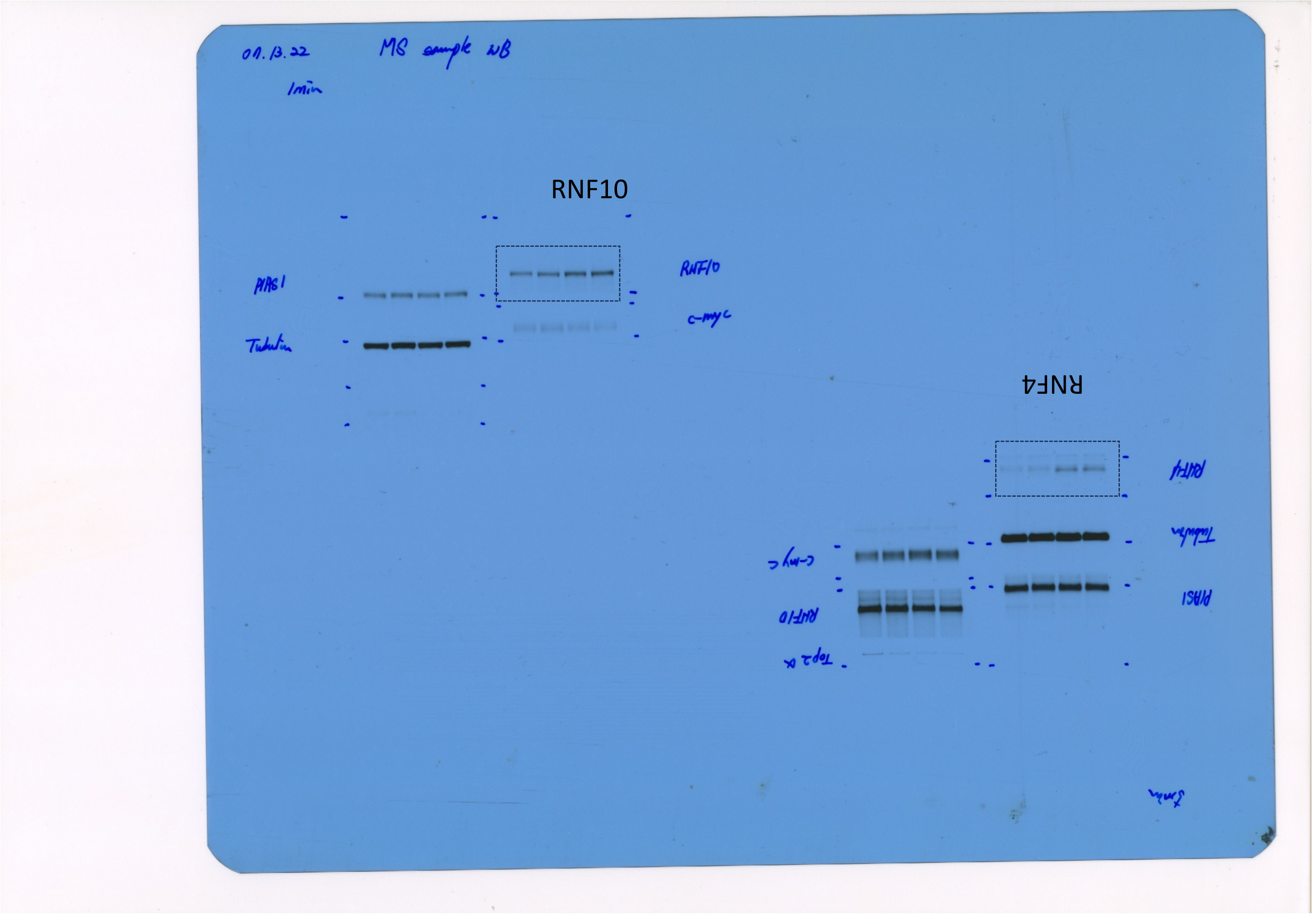

Full unedited bolt for Figure 4C

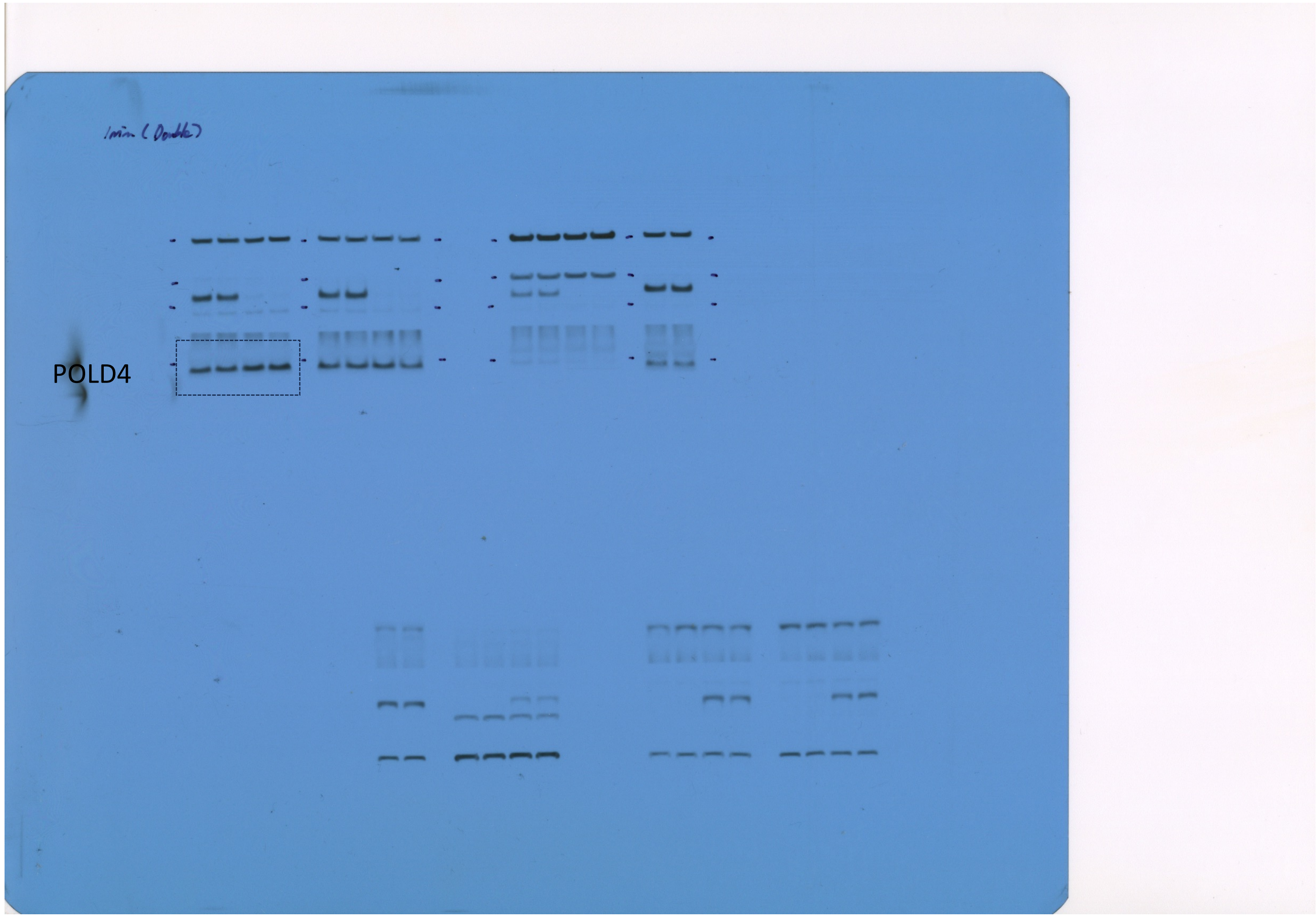

Full unedited bolt for Figure 4C

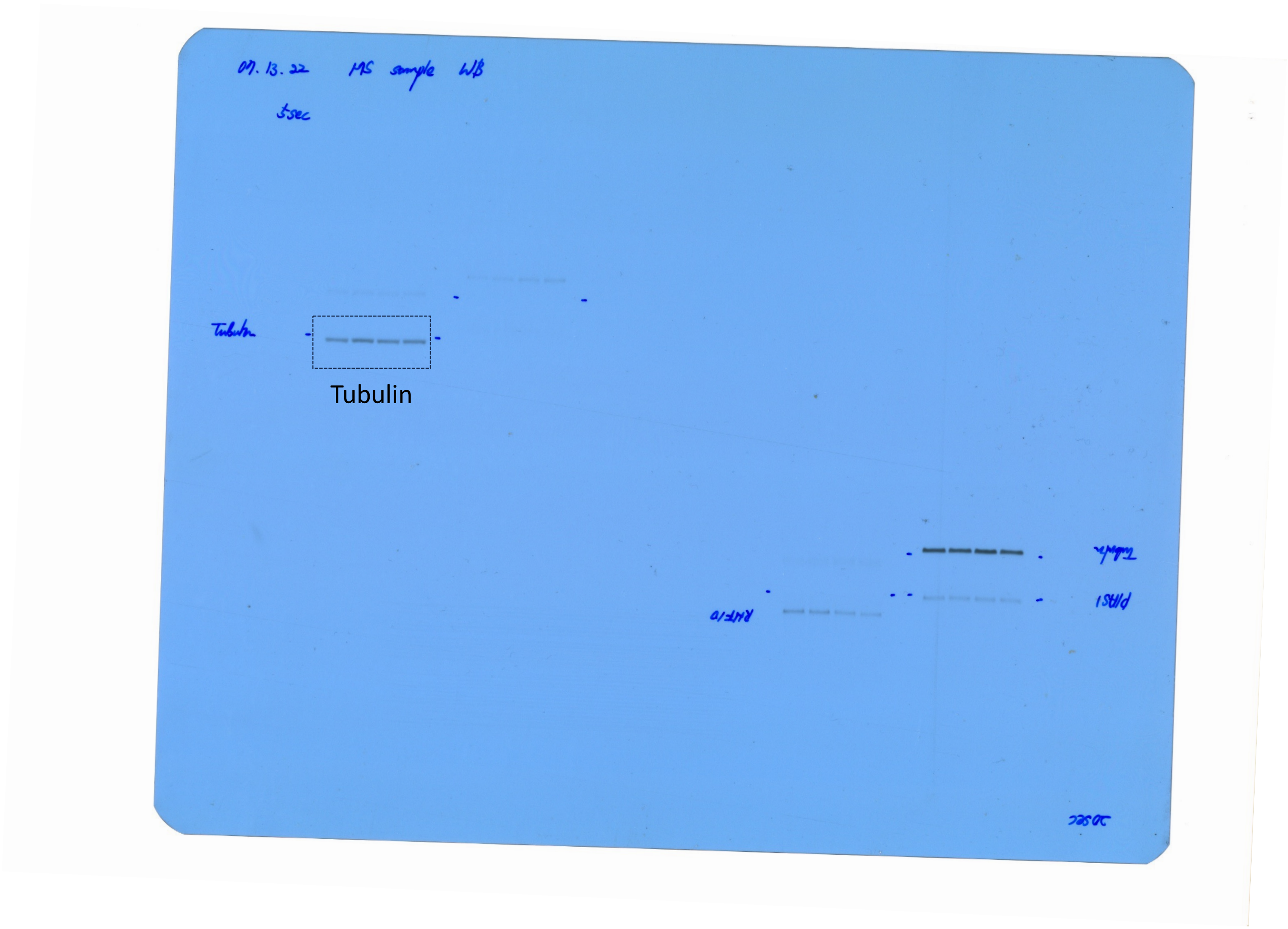

Full unedited bolt for Figure 5A

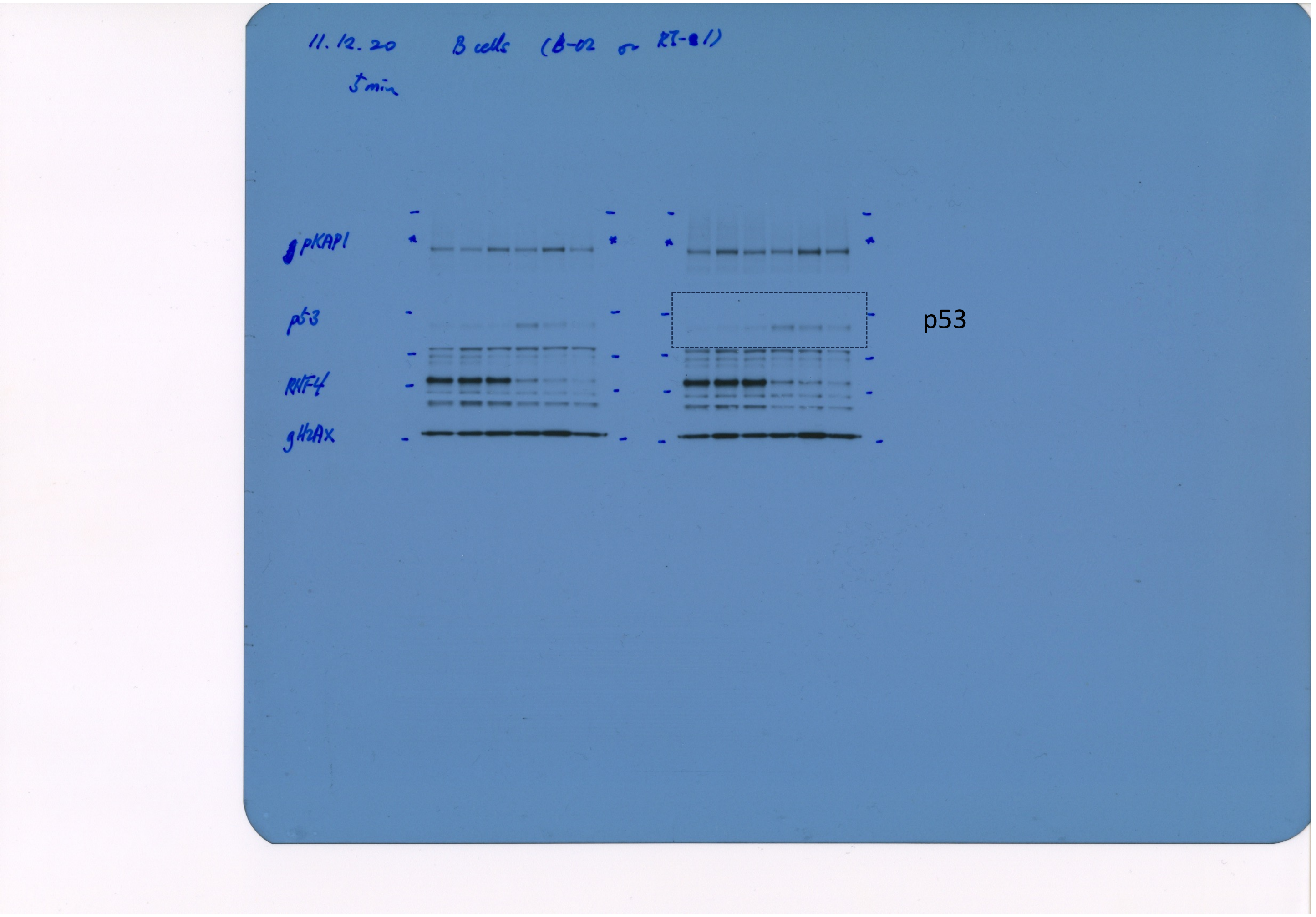

Full unedited bolt for Figure 5A

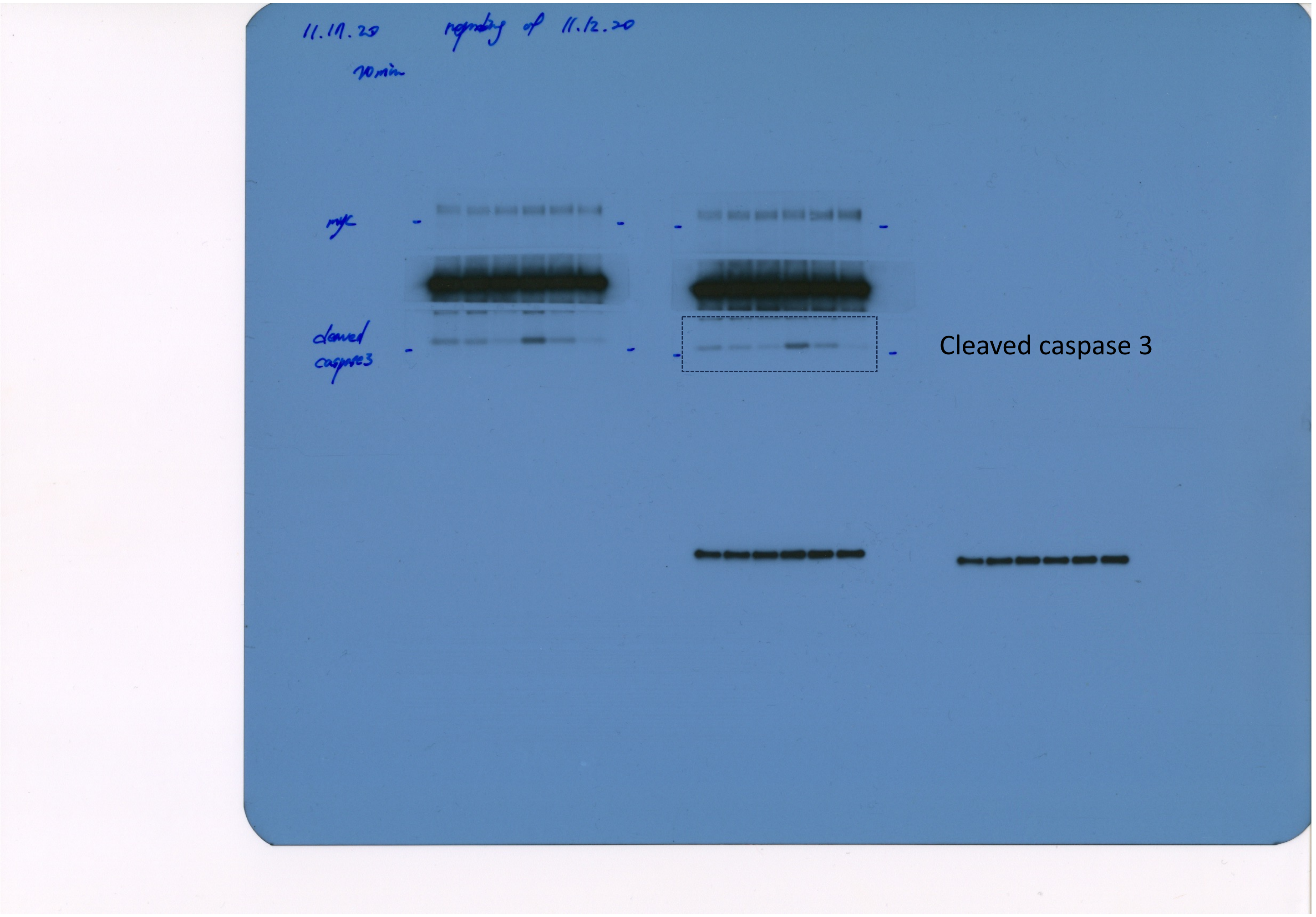

Full unedited bolt for Figure 5A

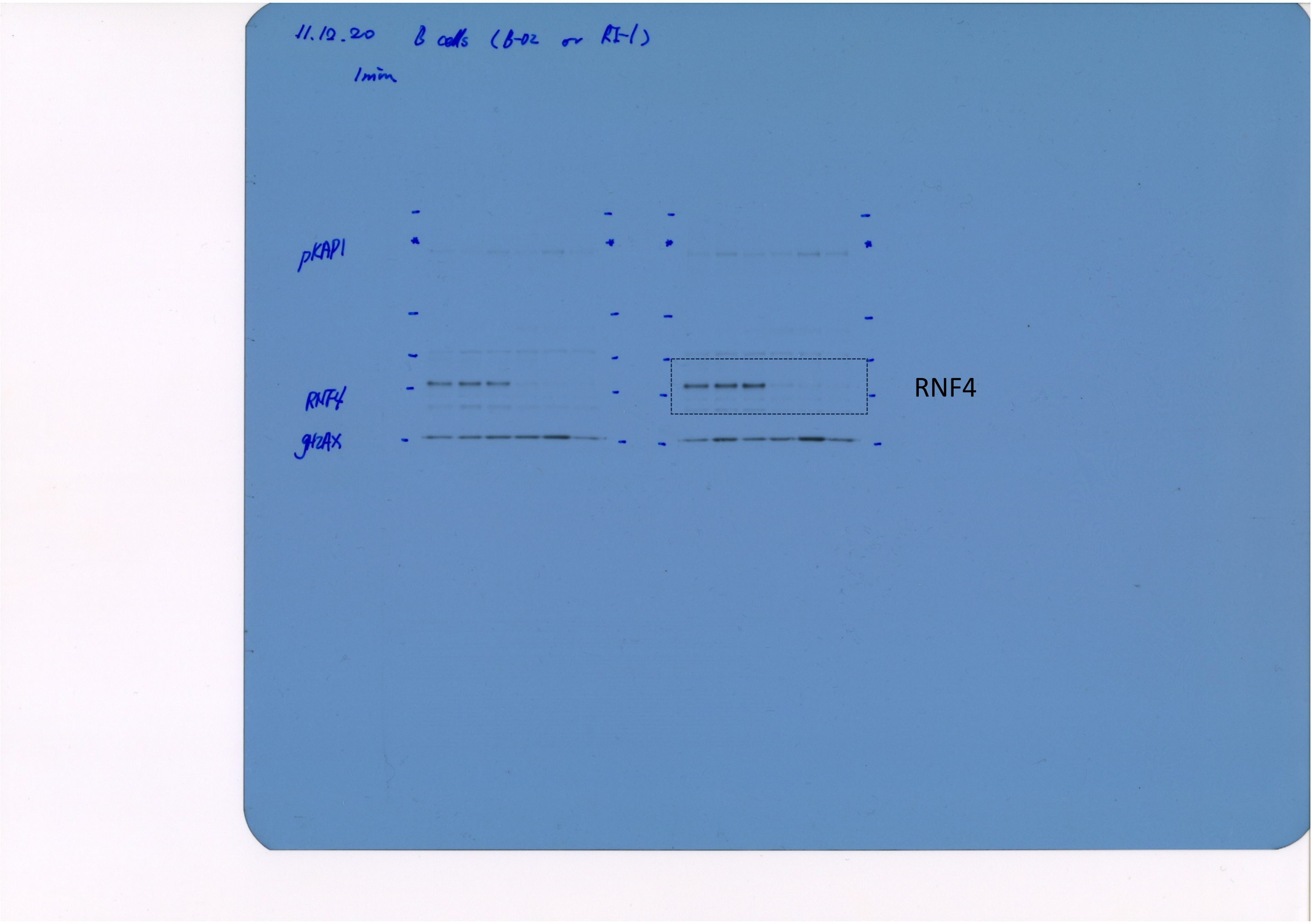

Full unedited bolt for Figure 5A

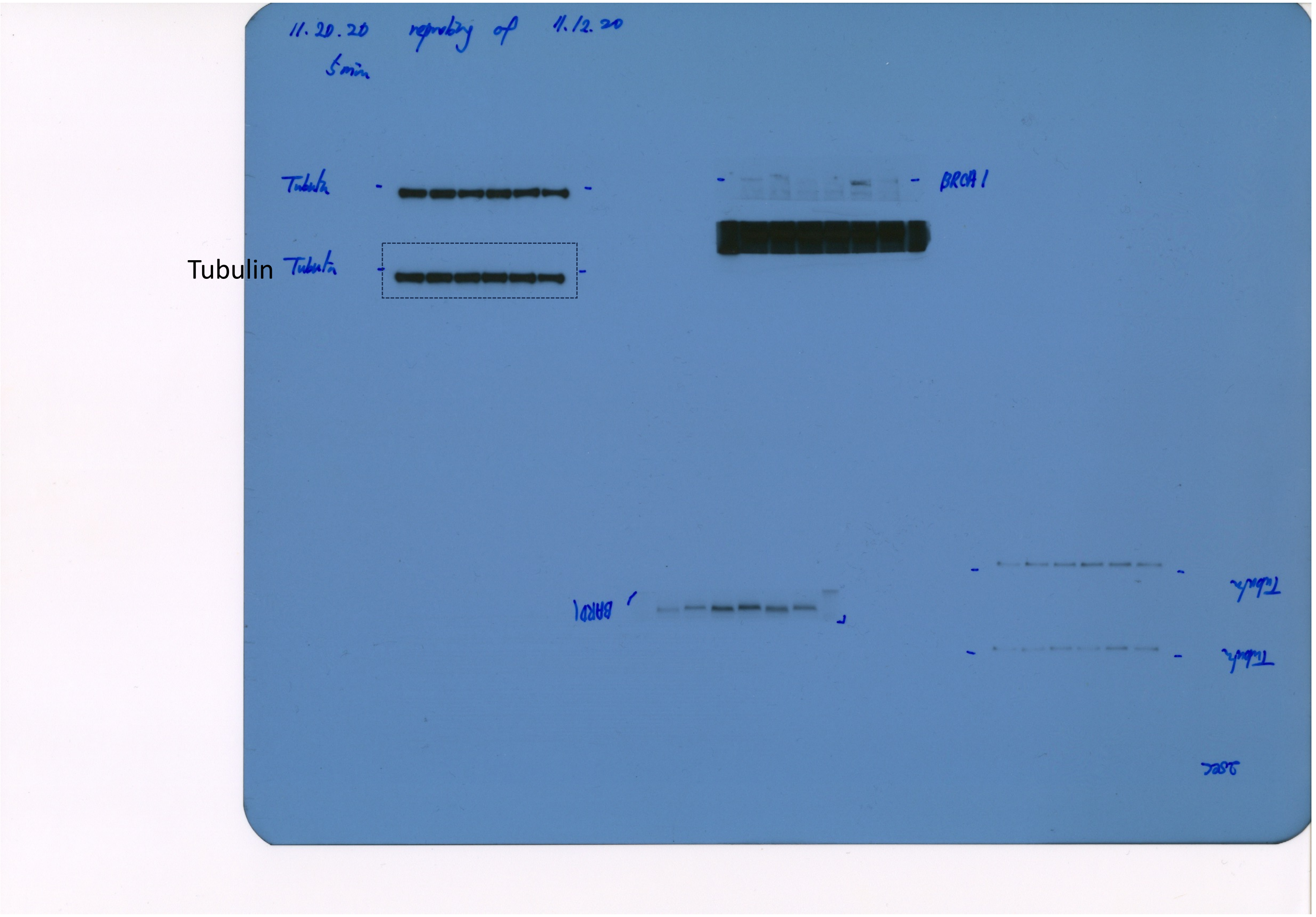

Full unedited bolt for Figure 5D

p53

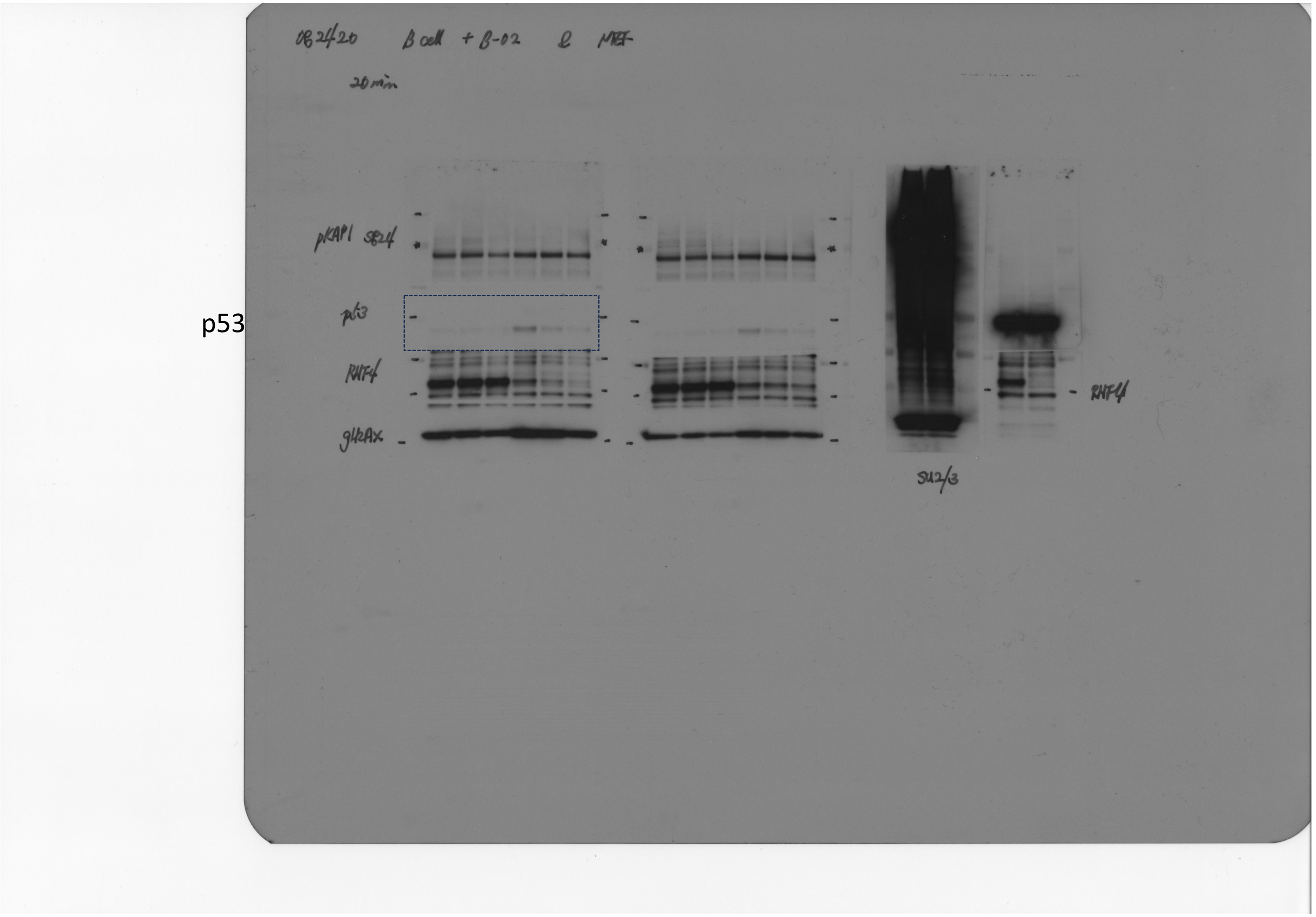

Full unedited bolt for Figure 5D

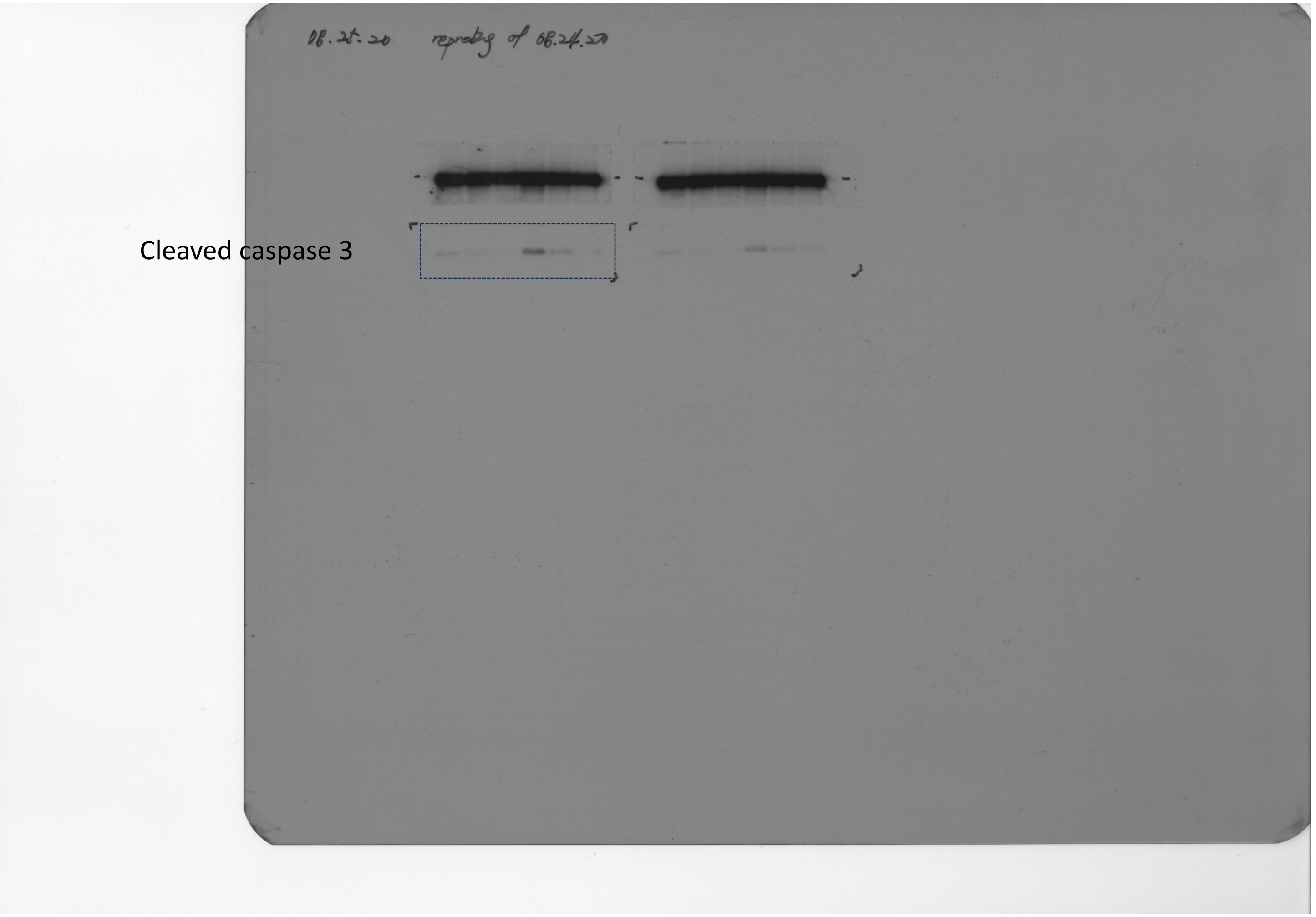

Full unedited bolt for Figure 5D

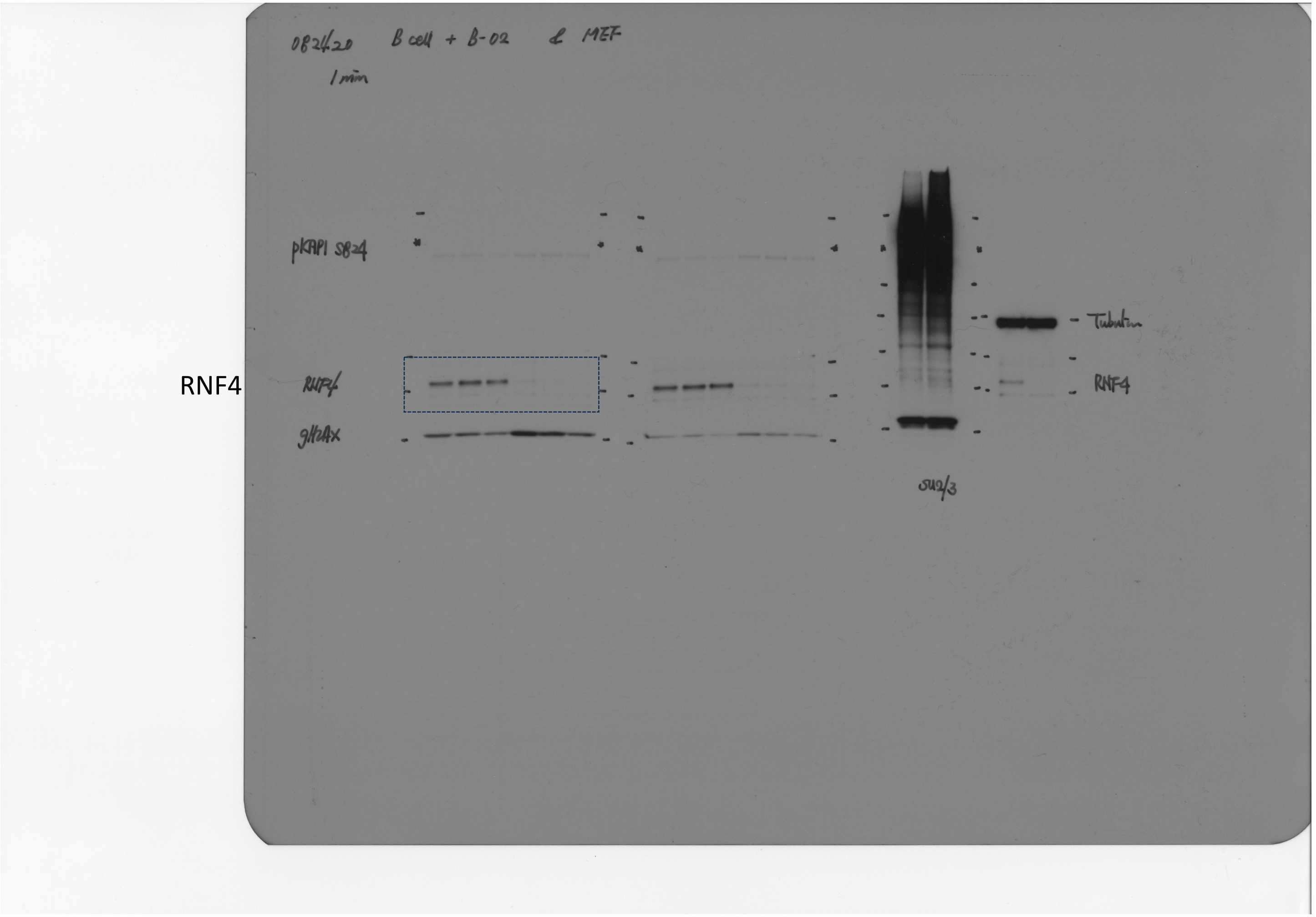

Full unedited bolt for Figure 5D

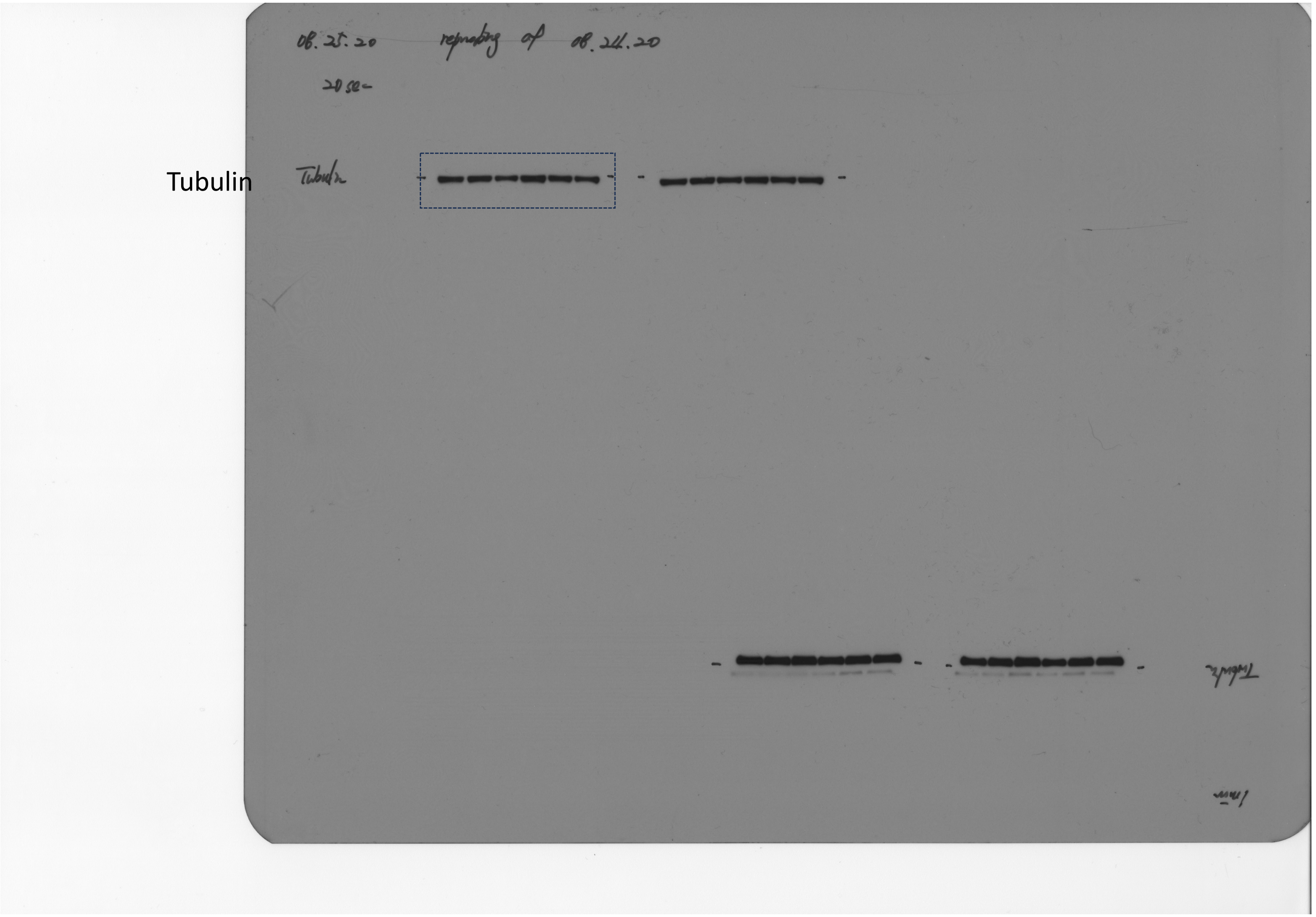

Full unedited bolt for Figure S3

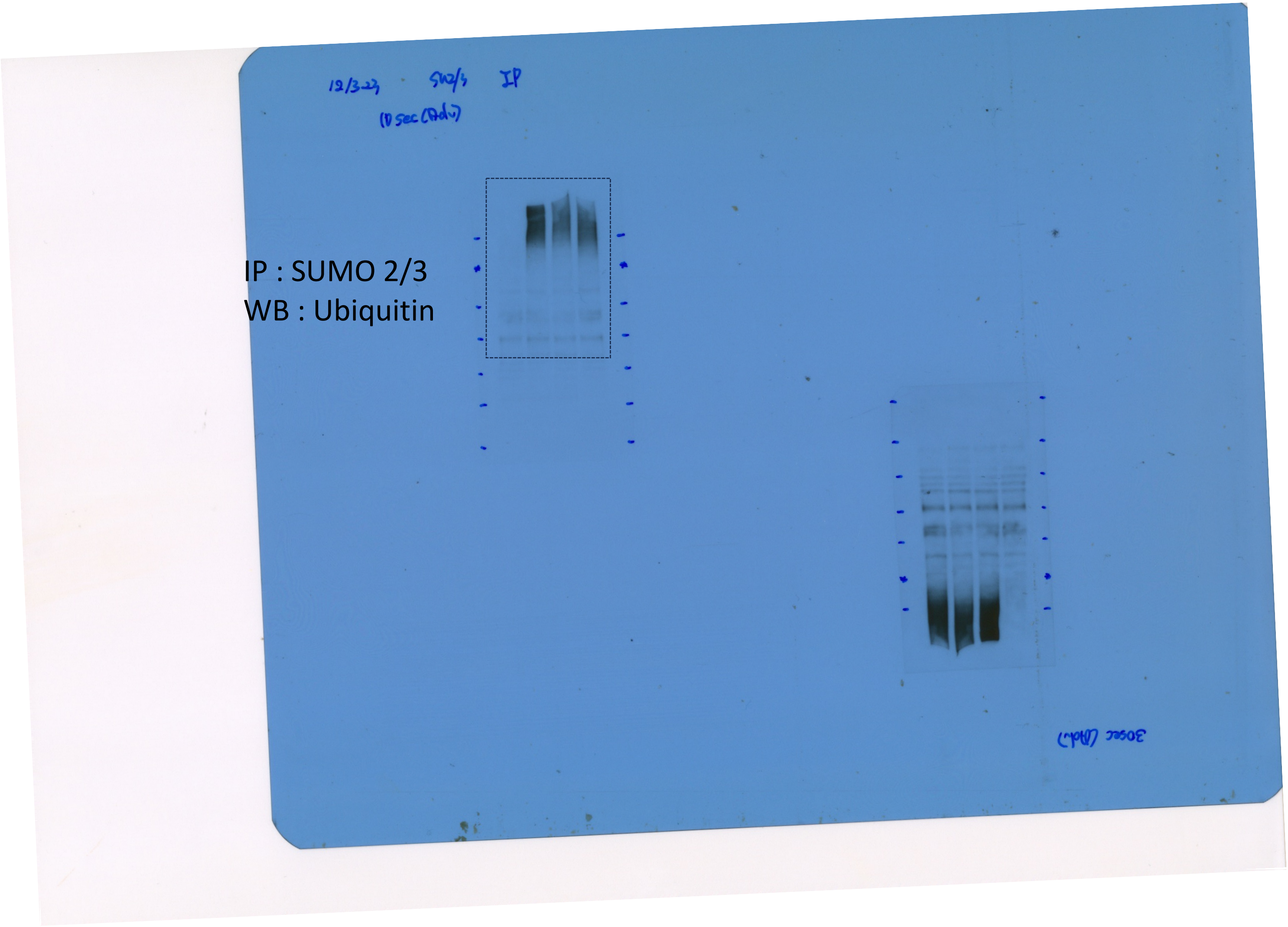

Full unedited bolt for Figure S3

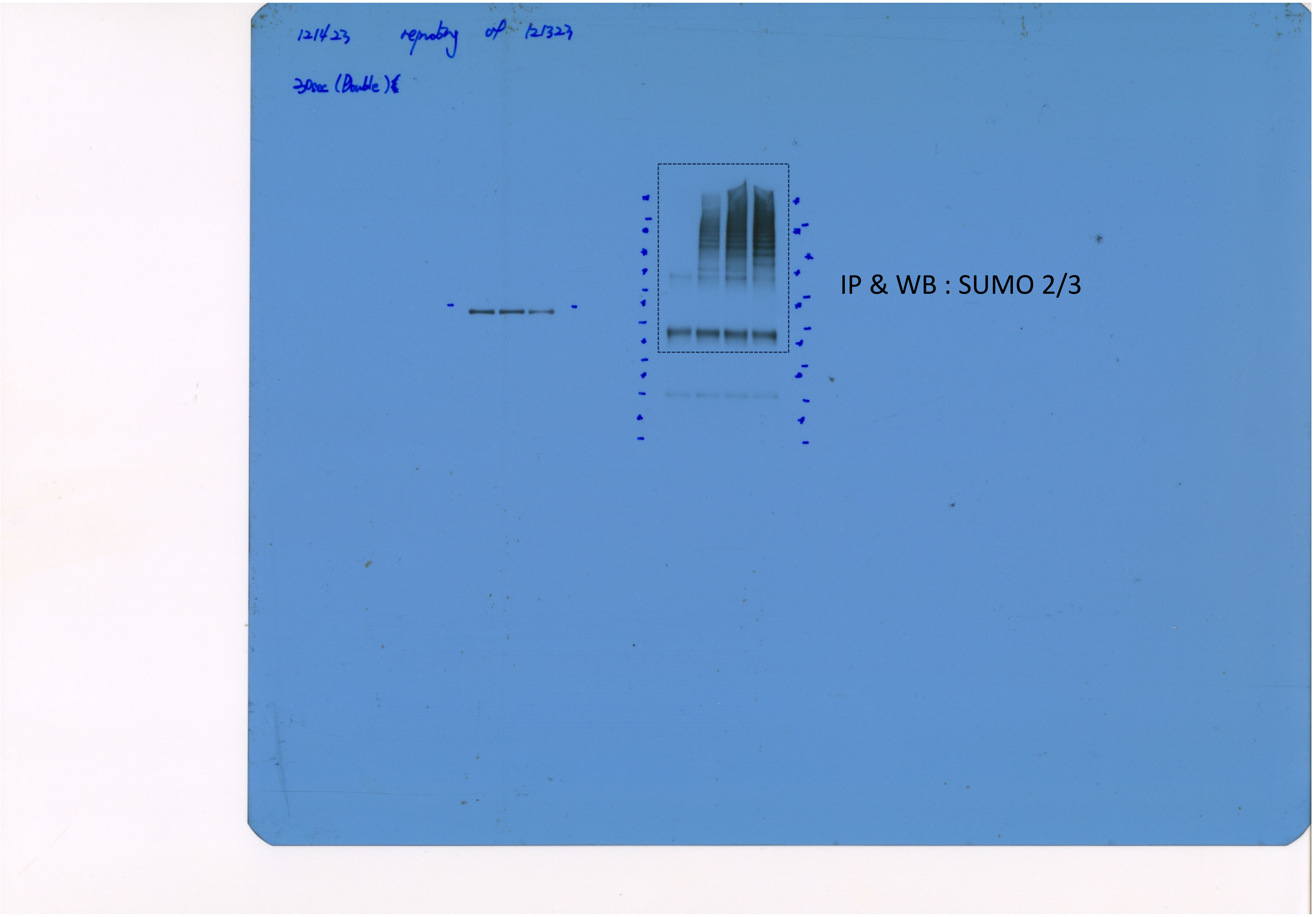

Full unedited bolt for Figure S4D

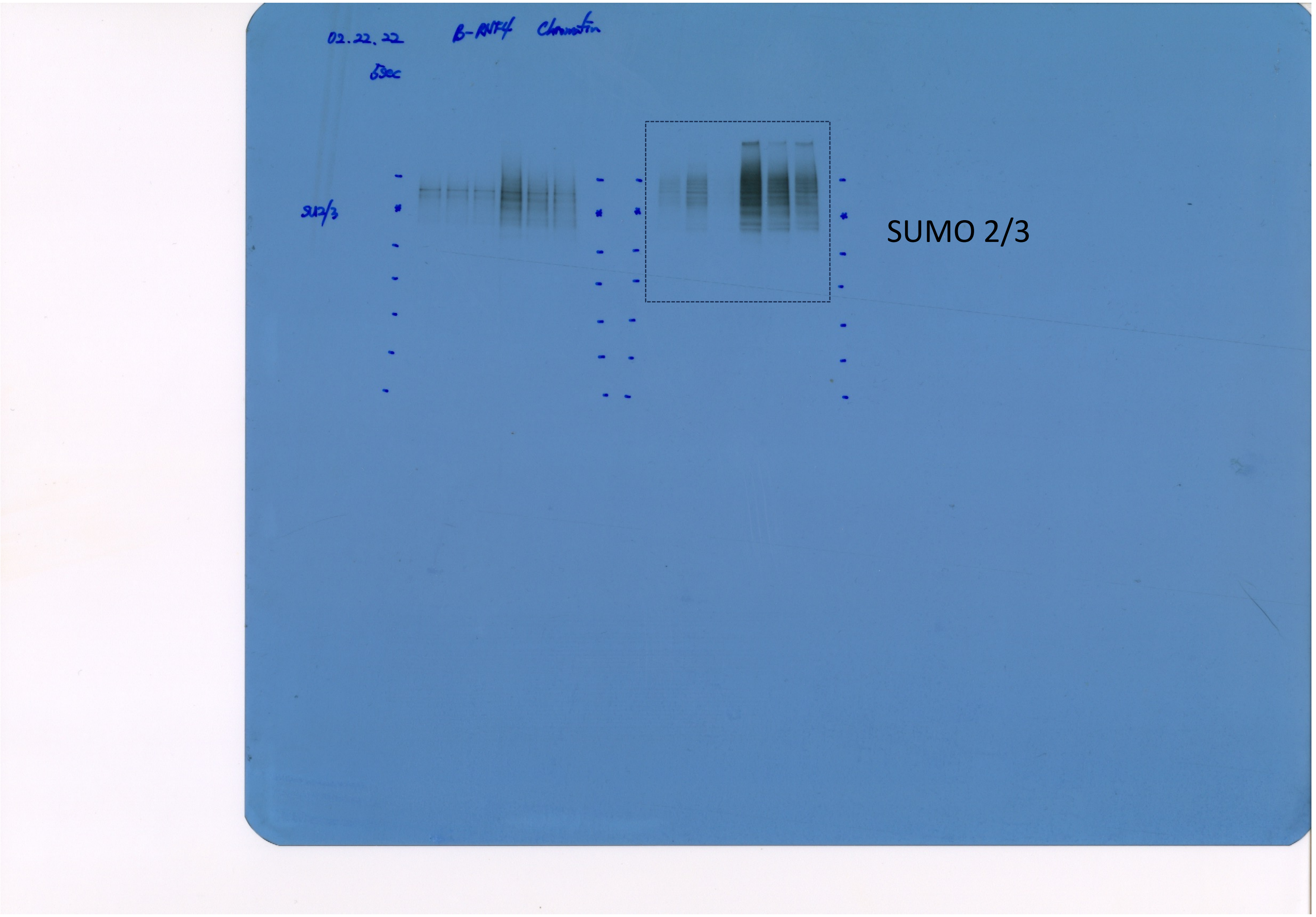

SUMO 2/3

Full unedited bolt for Figure S4D

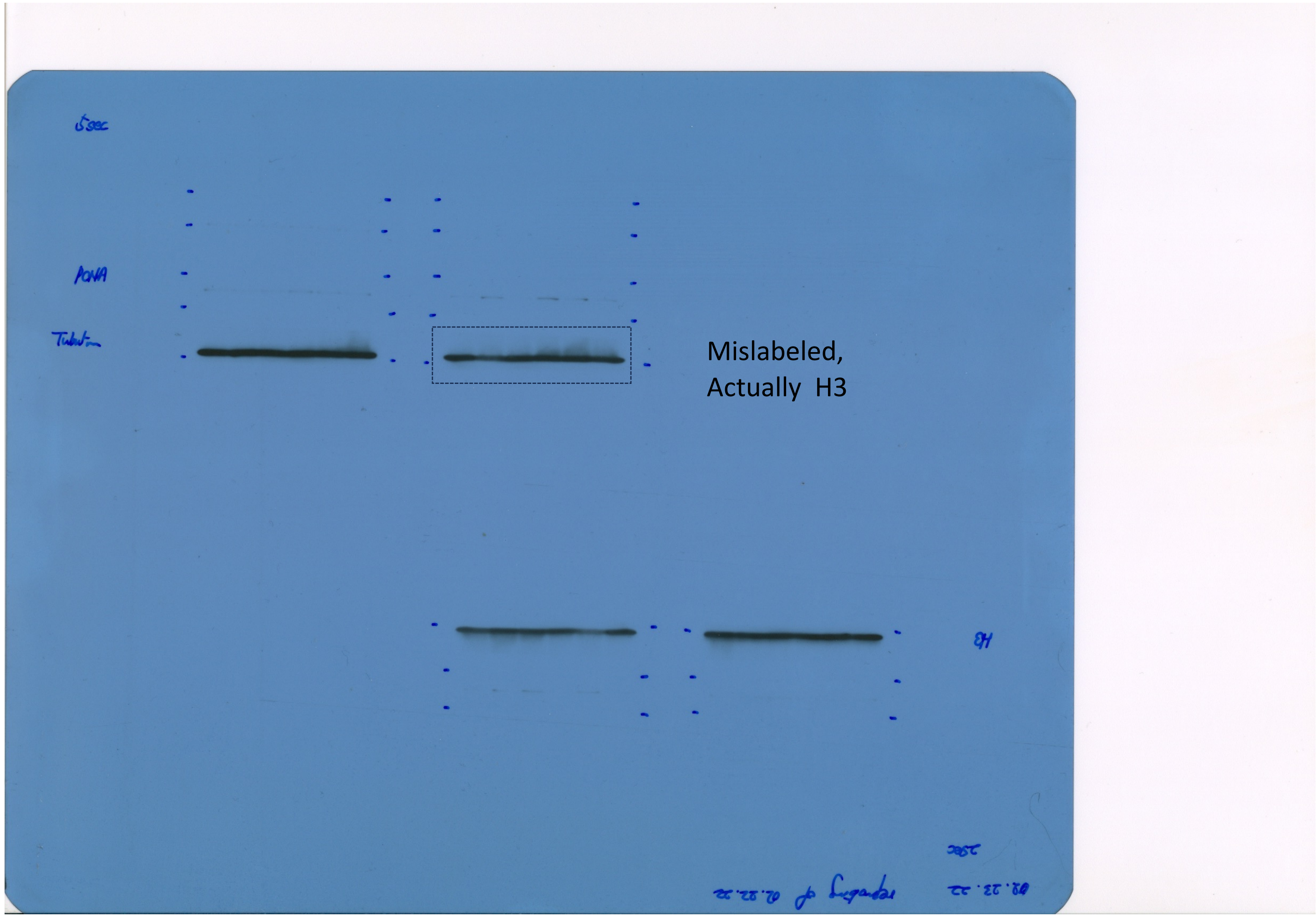

Full unedited bolt for Figure S5A

RNF4

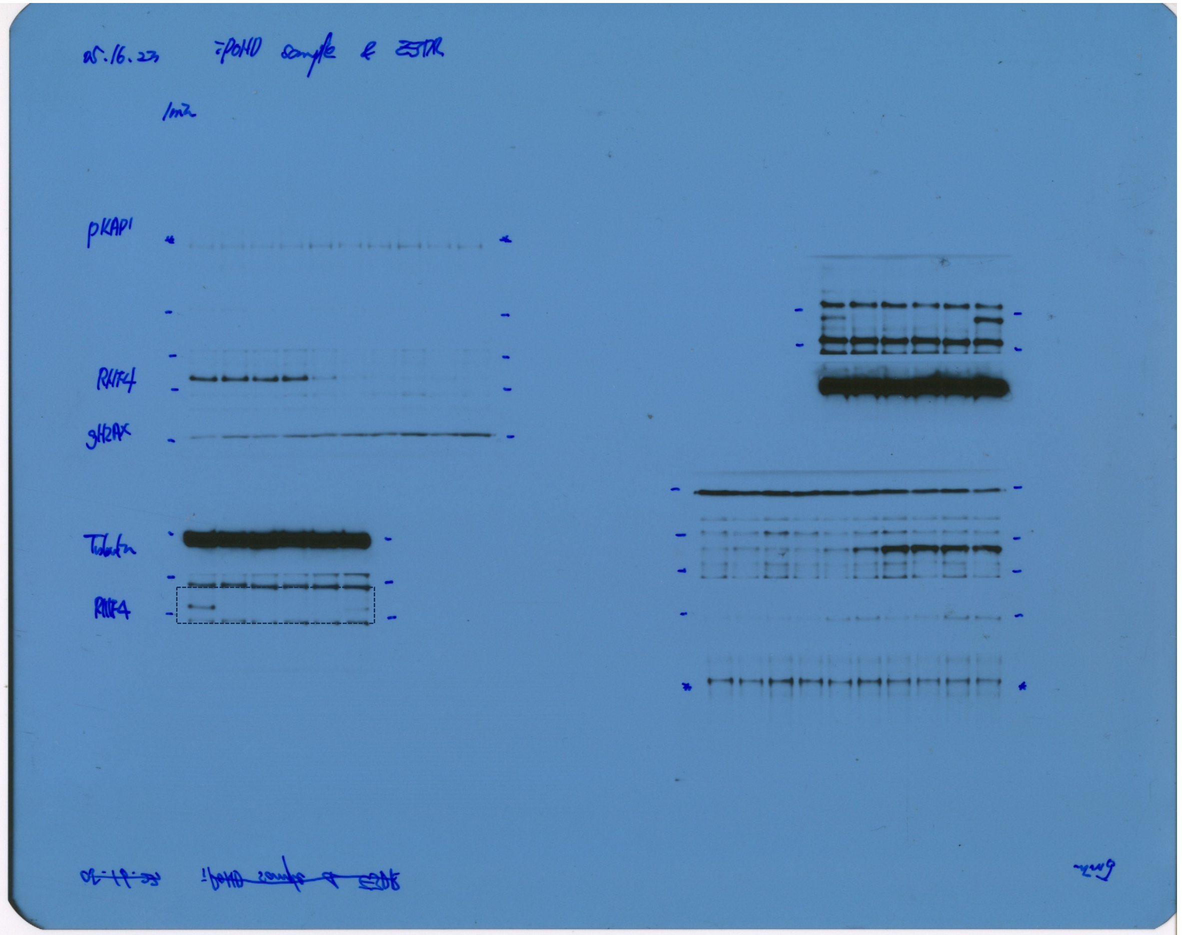

Full unedited bolt for Figure S5A

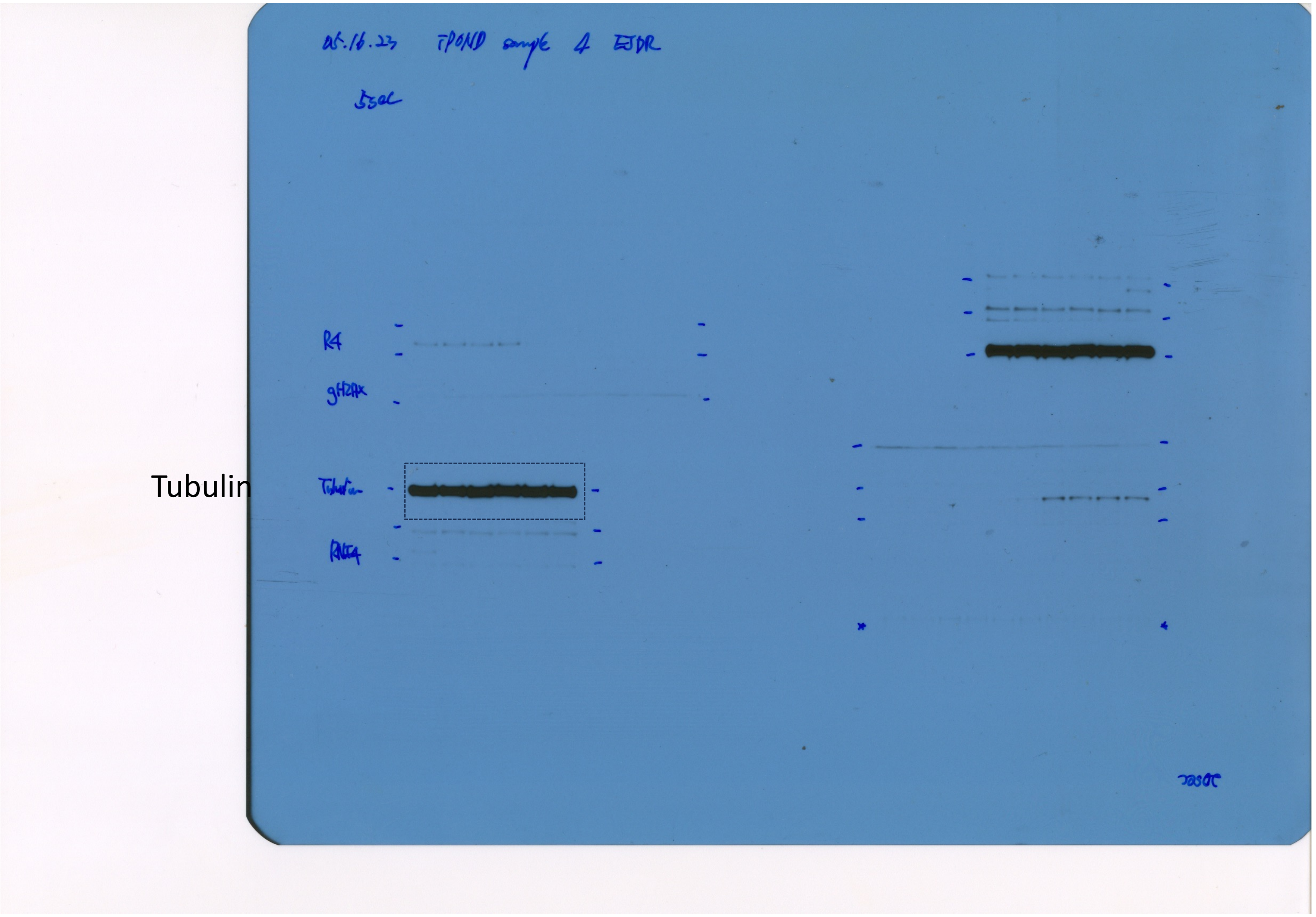

Full unedited bolt for Figure S5G

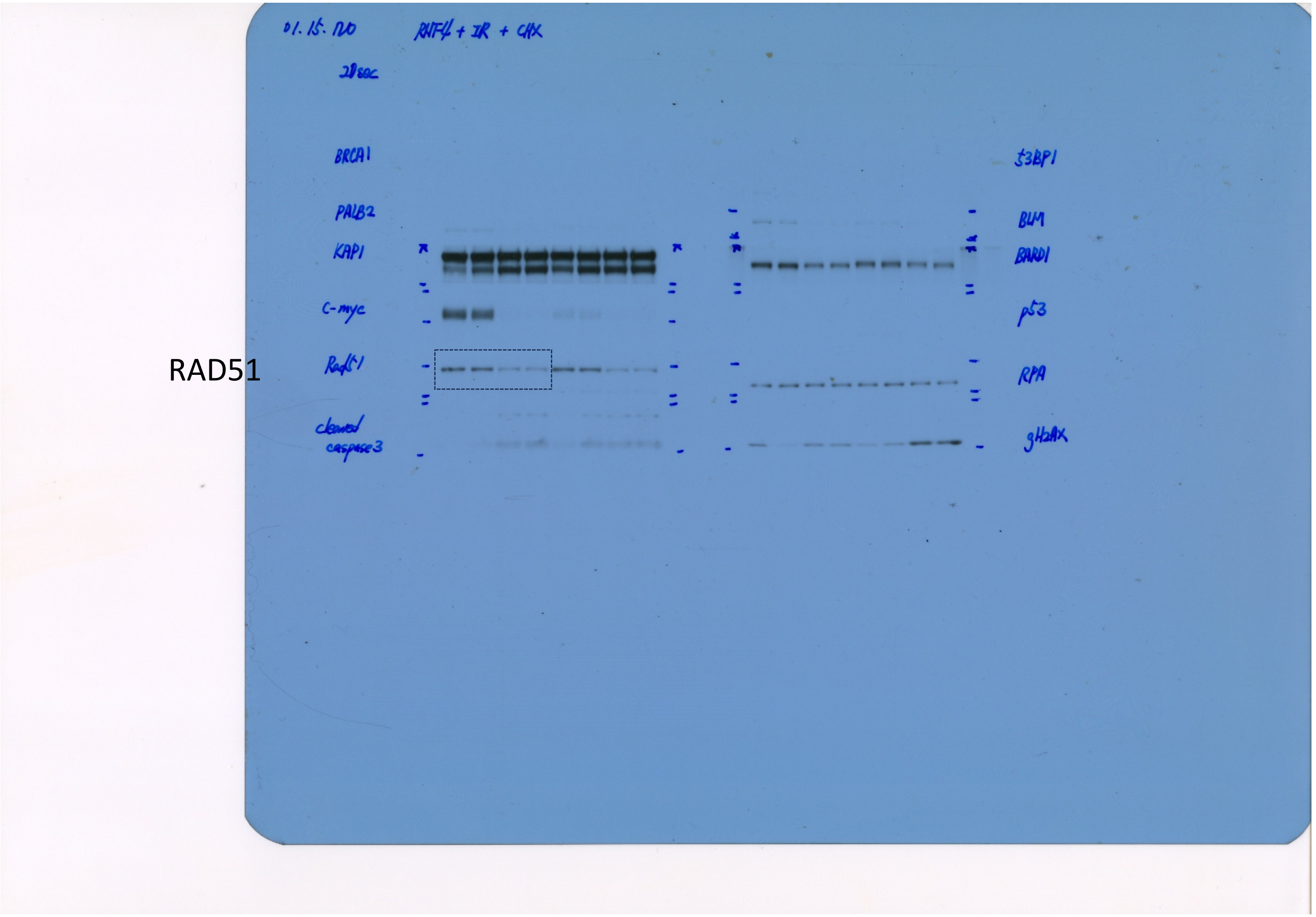

Full unedited bolt for Figure S5G

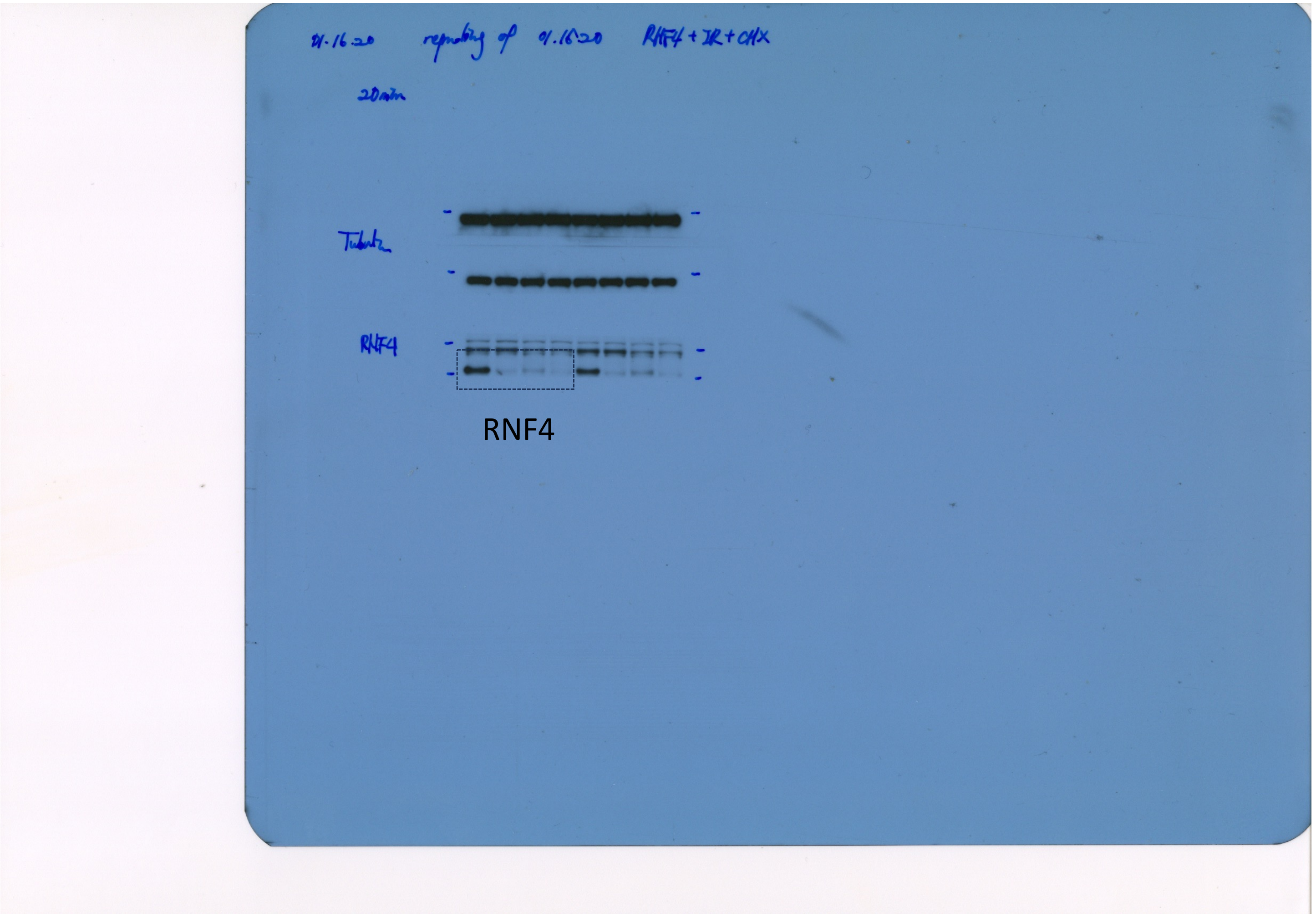

Full unedited bolt for Figure S5G

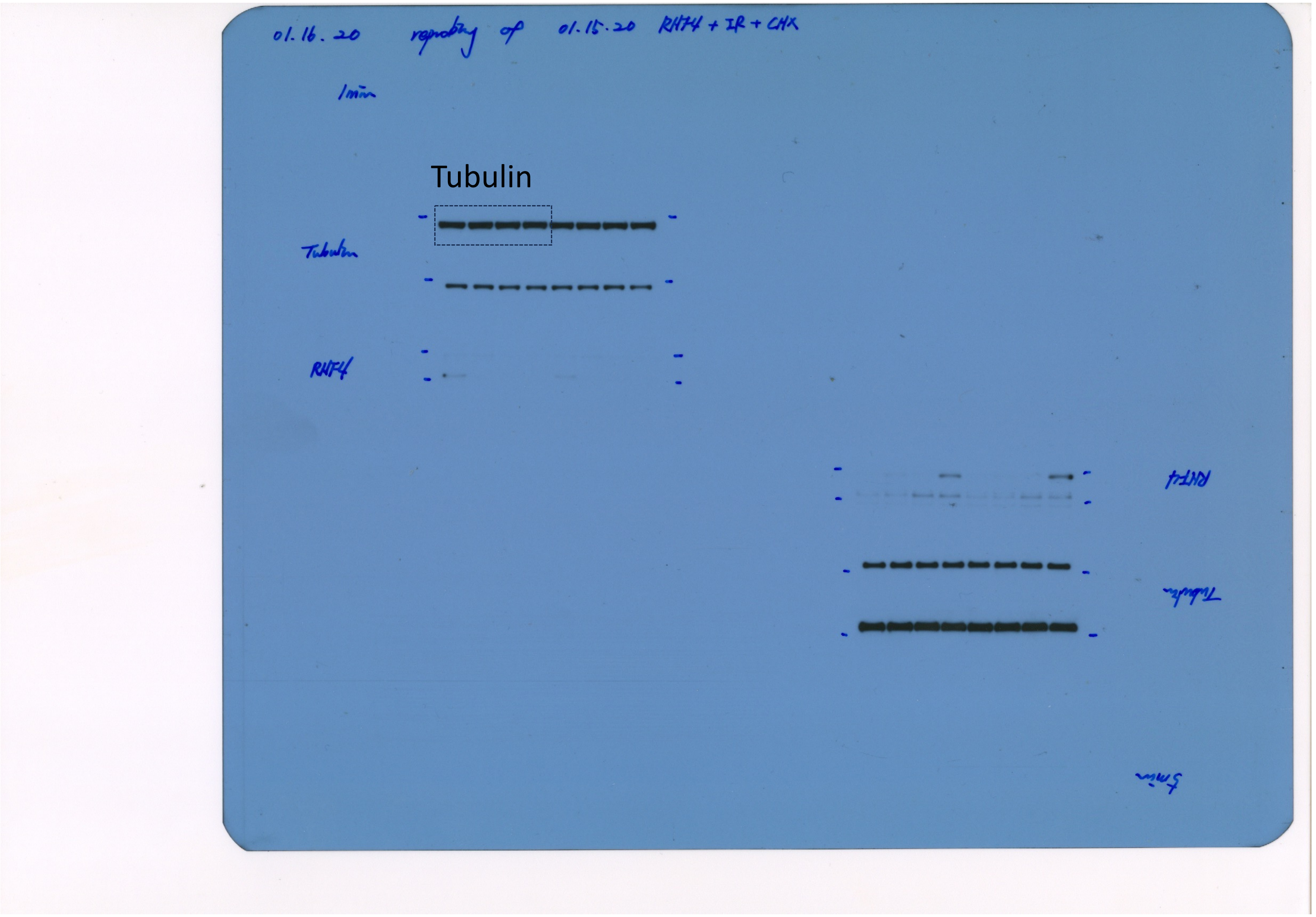

Full unedited bolt for Figure S5H

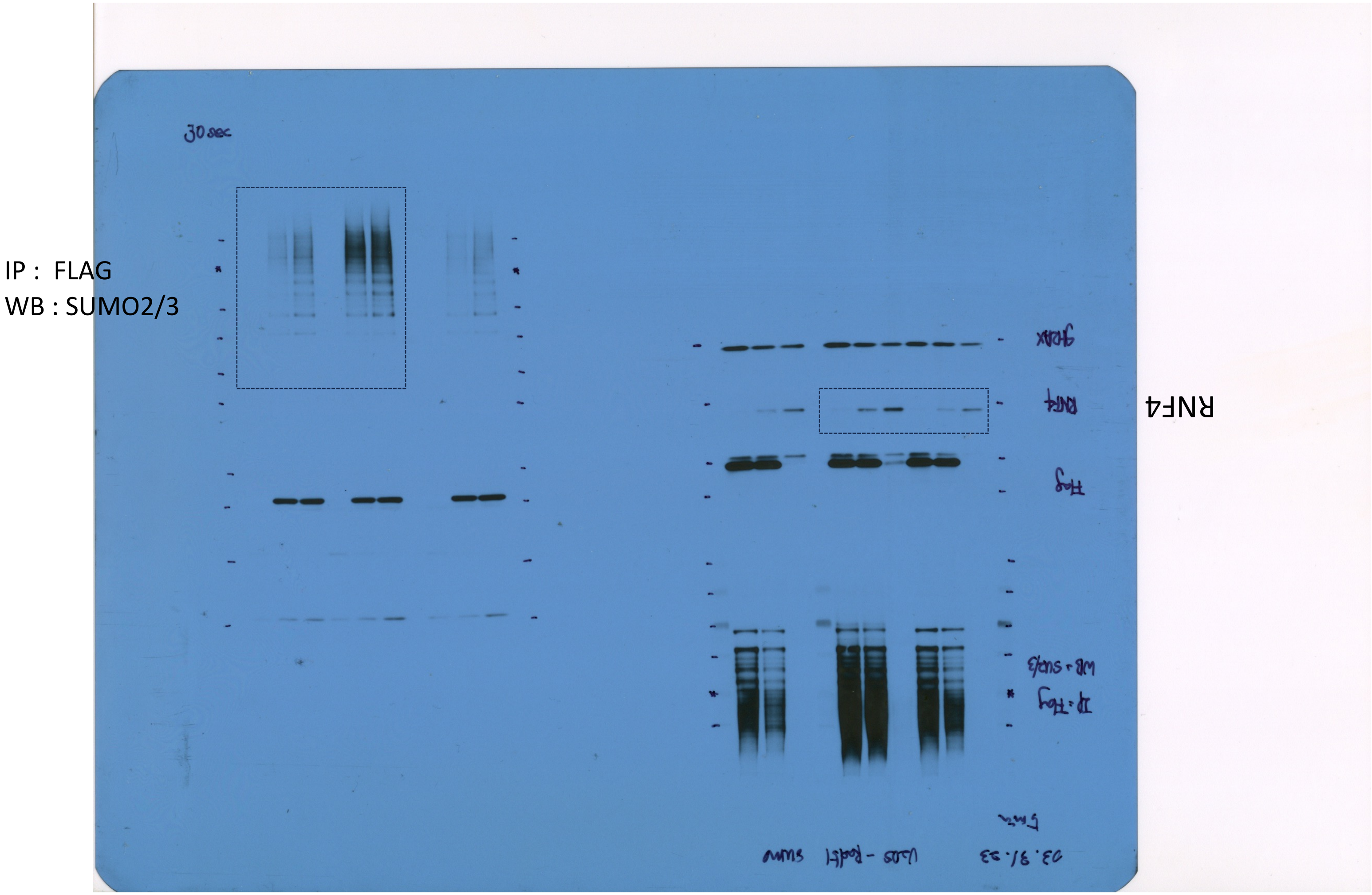

Full unedited bolt for Figure S5H

IP : FLAG
WB : Ubiquitin

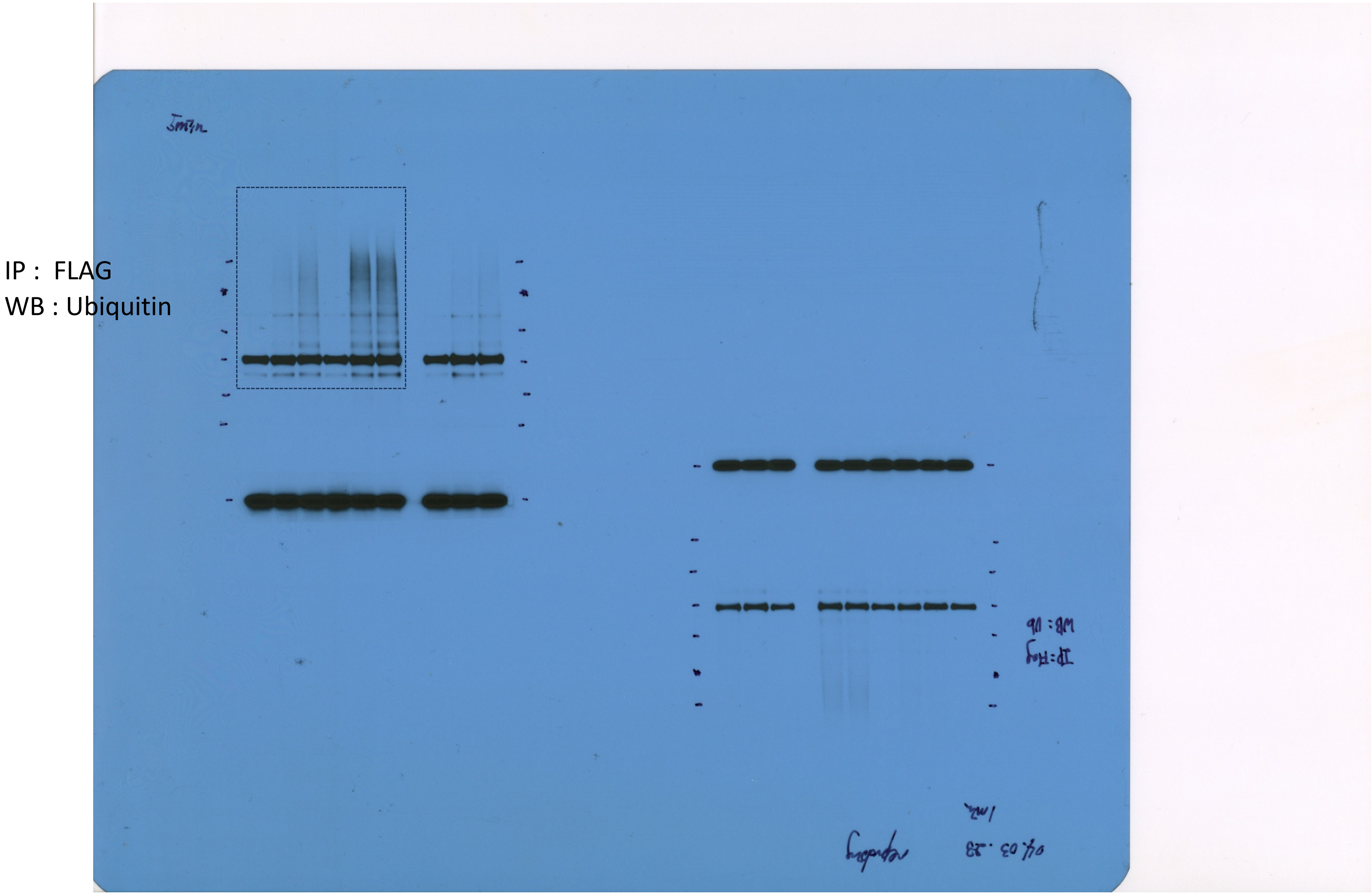

Full unedited bolt for Figure S5H

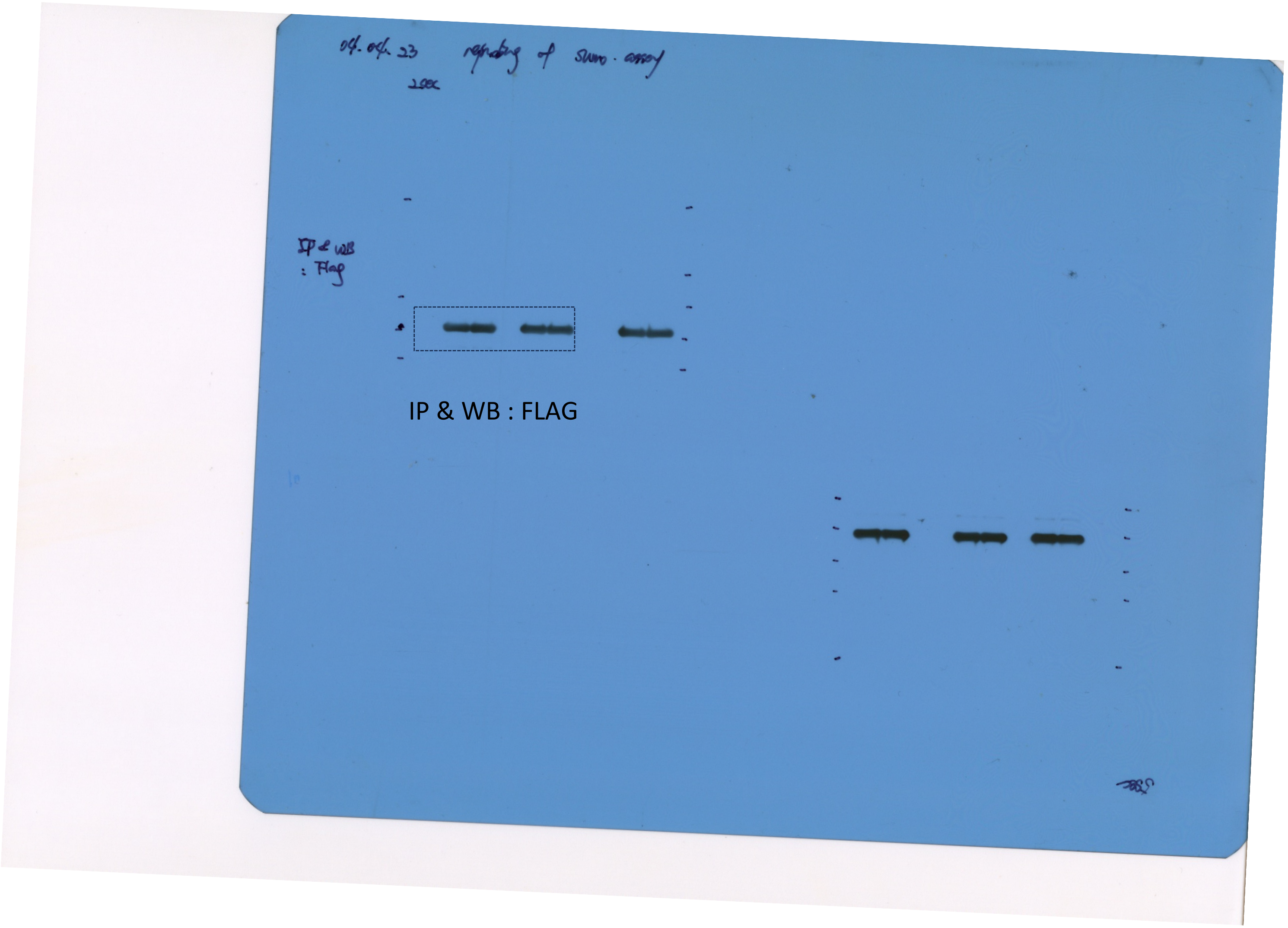

Full unedited bolt for Figure S5H

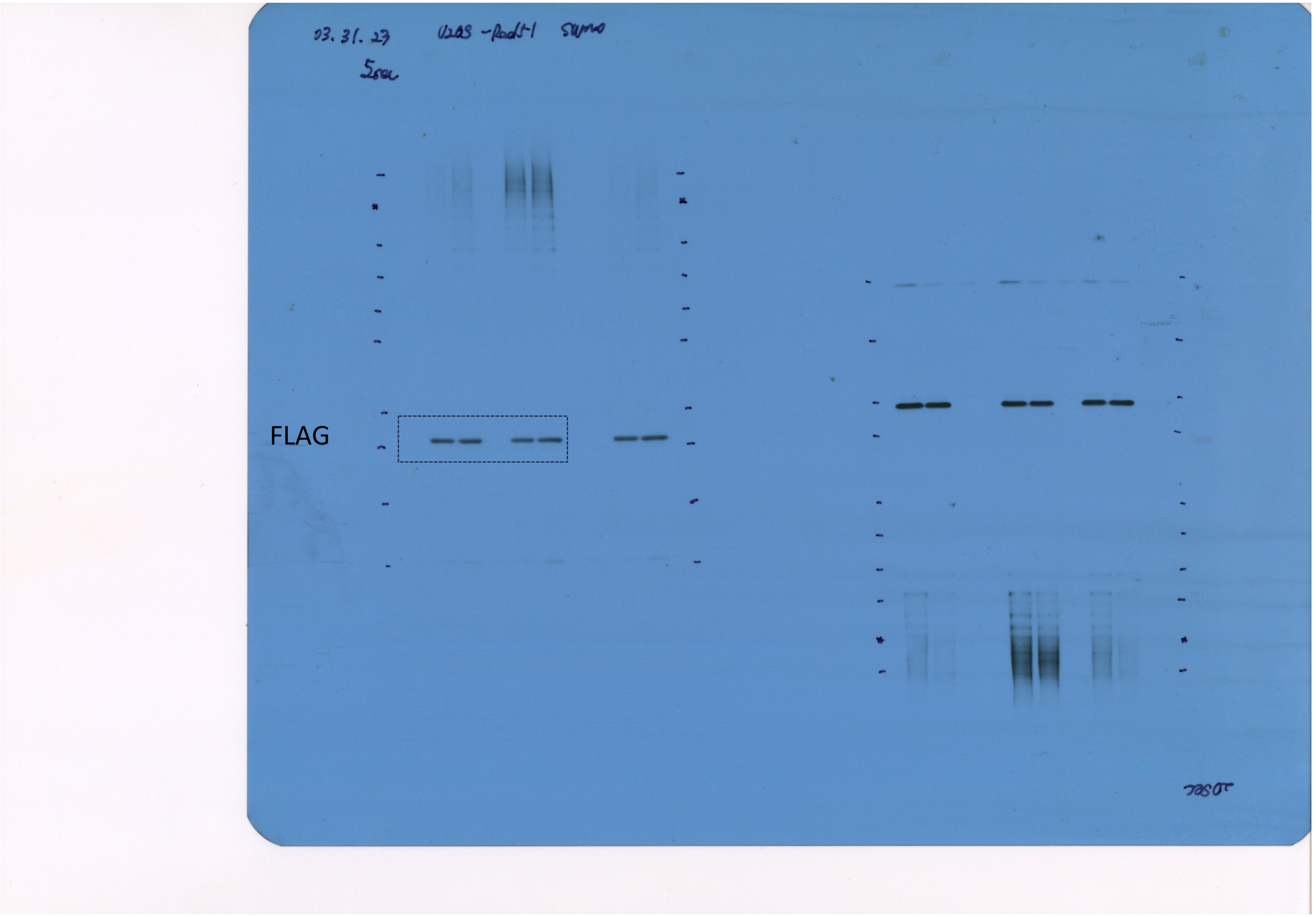

Full unedited bolt for Figure S5H

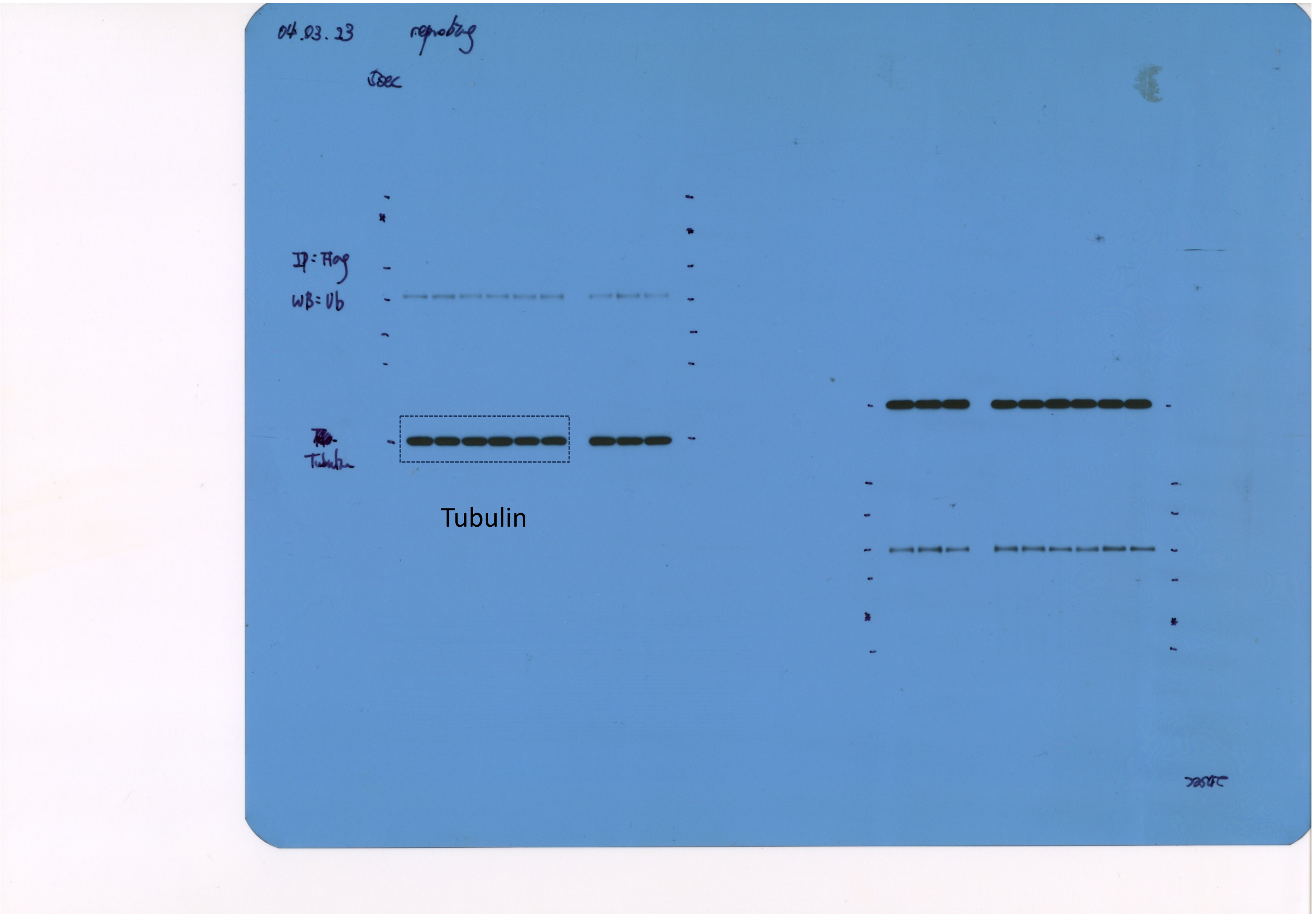

Supplement: Unedited blot and gel images [file jci-134-167419-s134.pdf]
